# *Plasmodium knowlesi* Heat Shock Protein 90s: *In Silico* Analysis Reveals Unique Druggable Structural Features

**DOI:** 10.3390/ijms262412065

**Published:** 2025-12-15

**Authors:** Michael O. Daniyan, Harpreet Singh, Gregory L. Blatch

**Affiliations:** 1 Department of Pharmacology, Faculty of Pharmacy, Obafemi Awolowo University, Ile-Ife 220005, Nigeria; mdaniyan@oauife.edu.ng; 2 Department of Bioinformatics, Hans Raj Mahila Maha Vidyalaya, Jalandhar 144008, India; harpreet@bioclues.org; 3 The Vice Chancellery, The University of Notre Dame Australia, Fremantle, WA 6959, Australia; 4 Biomedical Biotechnology Research Unit, Department of Biochemistry, Microbiology and Bioinformatics, Rhodes University, Makhanda 6140, South Africa

**Keywords:** heat shock proteins, Hsp90, HSPC1, HSPC3, molecular chaperones, *Plasmodium knowlesi*, protein folding, zoonotic malaria

## Abstract

The increasing threat of zoonotic malaria parasites of humans, such as *Plasmodium knowlesi*, make the search for improved pharmacotherapy imperative. Using protein sequence and structural analyses, phylogenetics, protein network mapping, protein–ligand interaction, and small molecule docking studies, we have identified for the first time the predicted structure, function, and druggability of the *P. knowlesi* heat shock protein 90s (PkHsp90s). Four isoforms were identified (in the cytosol, endoplasmic reticulum, mitochondrion, and apicoplast), and key structural differences were elucidated compared to human Hsp90s. In particular, the glycine-rich helix loop (GHL) motif of cytosolic PkHsp90 was predicted to have a straight conformation that forms a plasmodial-specific hydrophobic extension of the lid domain of the ATP-binding site, which was not observed for the cytosolic human Hsp90s, HSPC1 (Hsp90α), and HSPC3 (Hsp90β). Virtual screening identified for the first time a number of compounds from the ZINC database (ZINC22007970, ZINC724661072, and ZINC724661078) that were predicted to bind strongly to the GHL-associated pocket of PkHsp90, with weak or no binding to HSPC1. This study has provided a molecular framework in support of rational drug design, targeting PkHsp90s as a promising route for antimalarial drug development in the fight against zoonotic malaria.

## 1. Introduction

The predominant nature of malaria infection as a leading global health burden remains unabated. This is especially concerning with an estimated 263 million cases and approximately 600,000 deaths reported in 2024, mainly in tropical and subtropical regions [1]. Among the five *Plasmodium* species that infect humans, *Plasmodium knowlesi*, which was originally identified as a simian malaria species, has gained significant attention as an emerging zoonotic parasite, causing major human malaria in Southeast Asia (Malaysia, Indonesia, and Thailand) [2,3]. Its rapid upsurge in parasitaemia and severe disease progression within a 24 h erythrocytic cycle, coupled with multi-organ failure and death in untreated cases, distinguishes *P. knowlesi* from *P. falciparum* or *P. vivax* [2,4,5,6,7]. However, the need for accurate diagnosis remains a challenge that should be addressed as a matter of urgency to avoid misidentification because of the close resemblance of *P. knowlesi* and *P. malariae* when viewed under light microscopy [6,8,9].

*Plasmodium* parasites have evolved robust survival mechanisms in the face of hostile physiological stresses encountered in the human host. A critical component of the parasite’s adaptive response is the chaperones of the heat shock protein (Hsp) family, particularly heat shock protein 90 (Hsp90), a highly conserved molecular chaperone [10,11]. Hsp90 is essential for the proper folding and unfolding, stability, and function of a broad range of client proteins, many of which are involved in signal transduction, cell cycle control, and the stress response [12,13,14]. The functions of plasmodial Hsp90 that are crucial to parasite survival include sporozoite maturation, erythrocyte invasion, and the regulation of gene expression in response to environmental cues [10,15,16]. Structurally, Hsp90 comprises three functional domains: the N-terminal domain (NTD), which possesses ATP-binding and hydrolysis activity; the middle domain (MD), responsible for client protein interaction; and the C-terminal domain (CTD), which facilitates dimerization and interactions with co-chaperones [17]. The ATPase-driven conformational changes enable Hsp90 to cycle through different states required for client protein maturation and stabilization [18]. This dynamic chaperone cycle is modulated in close cooperation with Hsp70 chaperones via Hsp70/Hsp90 organizing protein (Hop) [19,20]. The functional interplay of plasmodial Hsp90 and associated chaperones and co-chaperones has been presented [21,22,23], revealing that these proteins operate in networks by partnering with each other and with the host chaperones.

Several reports have highlighted the potential of plasmodial Hsp90s as novel therapeutic targets by exploiting the unique structural differences between *P. falciparum* Hsp90s (PfHsp90s) and their human counterparts for selective drug targeting [24,25,26,27,28]. For instance, the cytosolic PfHsp90 ATP-binding site has a unique hydrophobic extension (encompassing the glycine-rich hinge loop or GHL motif) that could potentially be exploited for the design of novel inhibitors [29,30]. Several Hsp90 inhibitors, including those originally developed for cancer therapy, have shown potent antimalarial activity, including geldanamycin (GDM) and its derivative 17-AAG (17-allylamino-17-demethoxygeldanamycin), as well as radicicol and its analogues [31,32,33]. Efforts to develop more selective inhibitors have yielded promising candidates such as PU-H71, a purine-scaffold Hsp90 inhibitor that exhibits nanomolar potency against *P. falciparum*, with reduced cytotoxicity in mammalian cells, and shows potential for improved activity when combined with chloroquine [34]. The challenges of anticancer drug discovery studies have indicated that CTD inhibitors [35] and disruptors of protein–protein interactions (particularly those inhibiting Hsp90 association with co-chaperones and client proteins) [36] have the potential to address the drug resistance associated with activation of the heat shock response (HSR) by NTD pan-inhibitors. Furthermore, isoform-specific NTD inhibitors have been identified, particularly those selectively targeting cytosolic human HSPC3 (Hsp90β) over cytosolic human HSPC1 (Hsp90α), which appear to be both safe and effective, boding well for future clinical trials assessment as anticancer drugs [37]. Indeed, it is becoming increasingly evident that the historical clinical issues associated with NTD pan-inhibitors were primarily due to the inhibition of HSPC1 [37]. In summary, future drug repurposing for the development of novel PfHsp90-based antimalarial drugs should focus on isoform selectivity, CTD inhibitors, and modulators of protein–protein interactions.

However, despite these advances in PfHsp90 research, little is known about the Hsp90 orthologs in *P. knowlesi*, apart from the recent identification of four members: cytosolic *P. knowlesi* Hsp90, PkHsp90, PKNH_0107000; endoplasmic reticulum (ER) *P. knowlesi* glucose-regulated protein 94 kDa, PkGRP94, PKNH_1441400; mitochondrial *P. knowlesi* TNF receptor-associated protein 1, PkTRAP1, PKNH_0915900; and apicoplast *P. knowlesi* Hsp90_A, PkHsp90_A, PKNH_1238400 [38]. Although the *P. knowlesi* genome has been sequenced and annotated [39,40,41], comprehensive structural and functional characterization of its Hsp90 proteins is lacking. Understanding the molecular features of *P. knowlesi* Hsp90s is essential for assessing their functional roles and potential as drug targets. Therefore, this study aims to elucidate the structural and functional landscape of *P. knowlesi* Hsp90s through bioinformatics analyses, including sequence and structural alignments, domain annotation, homology modelling, phylogenetics, and protein network mapping. Also, using protein–ligand interaction analyses, small molecule docking studies, and virtual screening, we aim to predict potential small molecule inhibitors of cytosolic PkHsp90 from known or predicted inhibitors of cytosolic PfHsp90. This study will fill a critical knowledge gap and support rational drug design targeting these essential chaperones in the fight against zoonotic malaria.

## 2. Results

### 2.1. Identification and Characterization of Plasmodium knowlesi Hsp90 Isoforms

**Figure 1:**
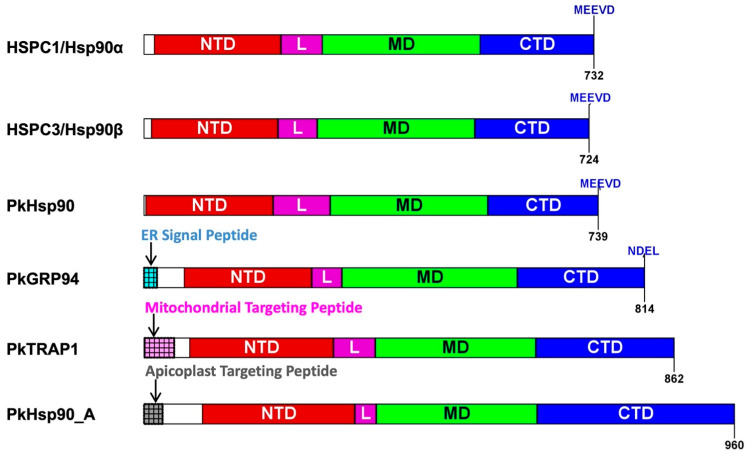
Domain organization of human (HSPC1/Hsp90α and HSPC3/Hsp90β) and *P. knowlesi* (PkHSP90; PkGRP94; PkTRAP1; and PkHsp90_A) Hsp90s. Abbreviations: NTD: N-terminal domain; L: charged linker region; MD: middle domain; and CTD: C-terminal domain. Textured boxes represent: ER signal peptide (light blue); mitochondrial targeting peptide (magenta); and apicoplast targeting peptide (grey). The name, localization and accession numbers of the protein sequences used to construct the linear domain organization layout are HSPC1/Hsp90α, cytosolic, NP_001017963.2; HSPC3/Hsp90β, cytosolic, NP_031381.2; PkHsp90, cytosolic, PKNH_0107000; PkGRP94, ER, PKNH_1441400; PkTRAP1, mitochondrion, PKNH_0915900; and PkHsp90_A, apicoplast, PKNH_1238400. The numbers at the end of each domain schematic represent the total number of amino acids in each protein. This linear domain layout was created using the standalone version of the IBS 1.0 (IBS: an illustrator for the presentation and visualization of biological sequence; available online: http://ibs.biocuckoo.org/; accessed on 28 September 2025).

**Figure 2:**
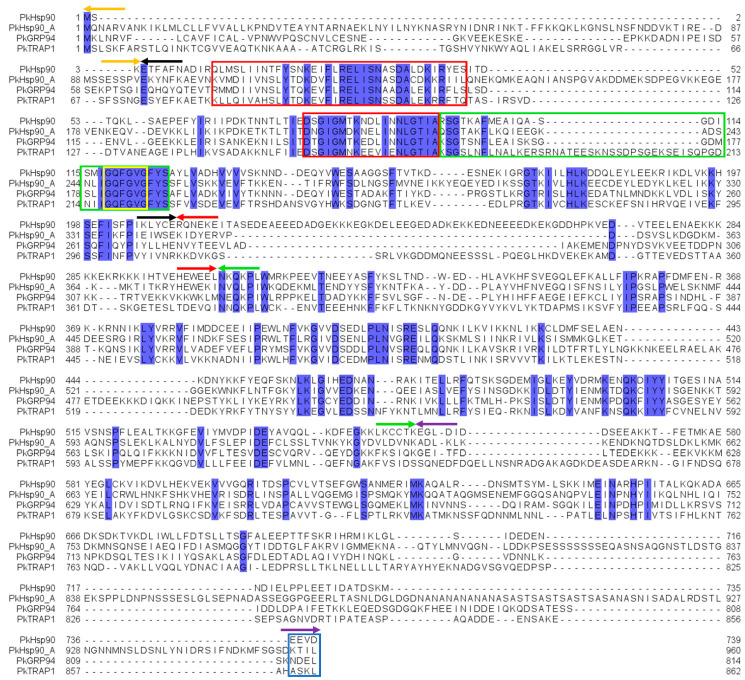
Multiple sequence alignment of *P. knowlesi* Hsp90 isoforms. Blue annotation indicates degree of conservation and sequence identity with a threshold of not less than 100%. Identified with arrows indicating the range of sequences for the protein domains are orange for the signal peptide/pre-NTD region; black for the NTD; red for the charged linker region; green for the MD; and purple for the CTD. In the yellow and blue boxes are the GHL motif “GxxGxG” and the C-terminal motifs, respectively. The red and green boxes identified critical sequences that make up the ATP-binding pocket and lid domains in PkHsp90, respectively, and their corresponding sequences in other isoforms. In between the red boxes is the region of very low sequence similarity. The *P. knowlesi* Hsp90 isoforms used are as follows: PkHsp90, PKNH_0107000; PkGRP94, PKNH_1441400; PkHsp90_A, PKNH_1238400; and PkTRAP1, PKNH_0915900. Alignment and annotation of sequence identity and conservation were generated using Jalview v2.11.5.0 [48]. The other annotation was conducted using Microsoft PowerPoint, and the image was processed with GIMP v2.10.14 software [49].

**Figure 3:**
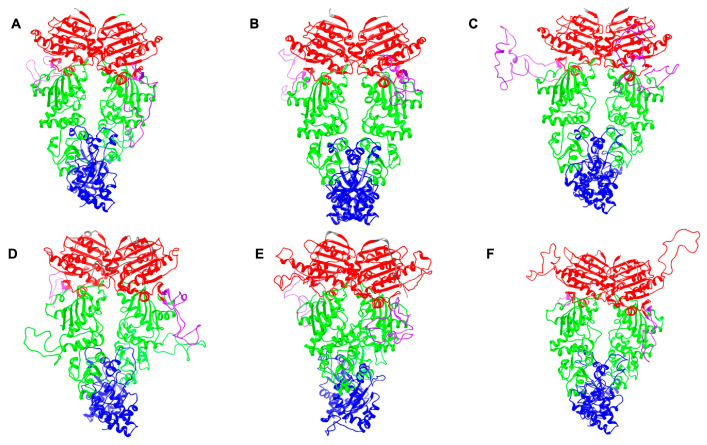
Predicted 3D structures of human ((**A**): HSPC1; (**B**): HSPC3) and *P. knowlesi* ((**C**): cytosolic PkHsp90; (**D**): ER PkGRP94; (**E**): mitochondrial PkTRAP1; and (**F**): apicoplast PkHsp90_A) Hsp90s as full-length dimers. The modelling was conducted with SWISS-MODEL (SWISS-MODEL: homology modelling of protein structures and complexes; available online: https://swissmodel.expasy.org/; accessed on 7 October 2025, homodimer mode), taking 5FWK, 8FFV, 7Y04 (electron microscopic structures), 5ULS, 4YIN, and 5ULS (X-ray crystallographic structures), respectively, as templates. The NTD, charged linker region, MD, and CTD are depicted in red, purple, green, and blue colours for both the monomers, respectively. The boundaries of the NTD and MD of the representative HSPC3 were derived from the X-ray crystallographic structures solved for these domains (NTD: PDB ID, 5UCJ and MD: PDB ID, 3PRY). Images for the predicted structures were rendered using UCSF Chimera 1.18 (UCSF Chimera: a visualization system for exploratory research and analysis; available online: https://www.cgl.ucsf.edu/chimera/; accessed on 7 October 2025).

The BLASTp search analysis using *P. falciparum* Hsp90 orthologs as queries identified four putative Hsp90 isoforms in the genome of all *Plasmodium* species used in this study (*P. knowlesi*, *P. vivax*, *P. berghei*, and *P. yoeli*). In *P. knowlesi*, these isoforms are (i) cytosolic PkHsp90 (PKNH_0107000); (ii) ER-localized PkGRP94 (PKNH_1441400); (iii) mitochondrial-localized PkTRAP1 (PKNH_0915900); and (iv) apicoplast-localized PkHsp90_A (PKNH_1238400) (Table S1). All isoforms exhibited the conserved signature Hsp90 domain organization, having an NTD (ATPase, co-chaperone binding), charged linker region (interdomain communication), MD (client binding, co-chaperone binding, ATPase activation), and CTD (dimerization, co-chaperone binding) (Figure 1 and Figure 2). The GHL motif “GxxGxG” was present in all Hsp90 isoforms of all *Plasmodium* species, forming part of the conserved sequence “IGQFGVGFYS”, similar to human HSPC1 (Figure S1), thereby confirming the identity of these proteins as belonging to the Hsp90 family. The MEEVD C-terminal motif required for co-chaperone interaction [13,14,21] was found in cytosolic PkHsp90 (Figure 1 and Figure 2), similar to cytosolic PfHsp90, suggesting that PkHsp90 has canonical co-chaperone–chaperone interactions [22,23]. In addition, the mitochondrial, ER and apicoplast signal sequences were identified in the respective isoforms for targeting to their sub-cellular organelles, as well as the C-terminal ER retention sequence (NDEL) in PkGRP94 (Figure 1 and Figure 2). The InterPro [42,43] analysis confirmed the presence of signature Hsp90 domains in all isoforms (Figure 2). Notably, the cytosolic PkHsp90 contained potential co-chaperone binding motifs and nuclear localization signals, suggesting that it may shuttle between the cytosolic and nuclear compartments under stress conditions. The predicted structures of all the *P. knowlesi* Hsp90 isoforms shared similar overall 3D domain architectures as the human cytosolic Hsp90s (HSPC1 and HSPC3) (Figure 3). However, noticeable differences included the longer charged linker region of cytosolic PkHsp90 (Figure 3C) and the presence of a major insertion in the NTD of apicoplast PkHsp90_A (Figure 3F), which both appeared as disordered coils on the dimeric structures.

### 2.2. Sequence Alignment and Phylogenetic Analysis

Multiple sequence alignments of full-length protein revealed that *P. knowlesi* Hsp90 isoforms shared >62% identity with their predicted orthologs in other *Plasmodium* species used in this study (*P. falciparum*, *P. knowlesi*, *P. vivax*, *P. berghei*, and *P. yoeli*) and less than 40% sequence identity with their paralogs (Table S1 and Figure S1). The cytosolic orthologs shared very high sequence identities (>93%) with one another and were the only plasmodial Hsp90 proteins with >67% sequence identities with cytosolic human HSPC1 (Figure S2 and Table S1). The next highest sequence identities were found with the apicoplast and ER orthologs, with >79% sequence identities (Figures S3 and S4, Table S1). The lowest sequence identities were found among the mitochondria orthologs (with as low as 62.56%) (Figure S5 and Table S1). As expected, the NTD encompassing the highly conserved ATPase domain exhibited the greatest level of sequence identity (Figure 1 and Table S1). The conservation of the overall Hsp90 domain architecture and signature motifs, especially the ATPase domain and co-chaperone binding sites, suggests that the *P. knowlesi* Hsp90 isoforms are functionally similar to their homologues and, like the *P. falciparum* Hsp90 isoforms, may perform essential roles in parasite stress management and life cycle progression [44,45]. It is also possible that known small molecule inhibitors of the *P. falciparum* Hsp90 isoforms may similarly modulate *P. knowlesi* Hsp90 isoforms.

**Figure 4:**
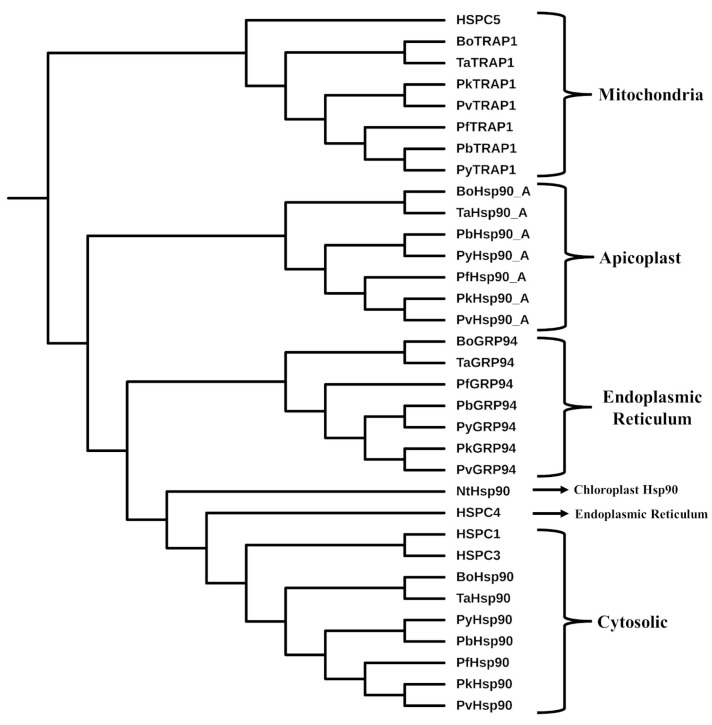
Phylogenetic analysis of plasmodial and human Hsp90 isoforms. Pb, Pf, Pk, Pv, and Py are *P. berghei*, *P. falciparum*, *P. knowlesi*, *P. vivax*, and *P. yoeli*. Hsp90, Hsp90_A, TRAP1, and GRP94 are the cytosolic, apicoplast, mitochondrial, and ER Hsp90 isoforms, respectively. HSPC1 (cytosolic), HSPC3 (cytosolic), HSPC4 (ER), and HSPC5 (mitochondrial) are the human Hsp90 isoforms. Control proteins included in the phylogenetic analysis were additional apicomplexan Hsp90s (Bo and Ta are *Babesia ovata* and *Theileria anniculata*), namely, TaHsp90 (TA12105), TaTRAP1 (TA06845), TaGRP94 (TA06470), TaHsp90_A (TA10720), BoHsp90 (BOVATA_007200), BoTRAP1 (BOVATA_017520), BoGRP94 (BOVATA_020650), and BoHsp90_A (BOVATA_003160), as well as NtHsp90 (LOC107797756), the chloroplast Hsp90 from *Nicotiana tabacum*. Phylogenetic analyses were performed using the NGPhylogeny.fr online resource as detailed in the Materials and Methods Section 4.3. Final image processing was conducted with GIMP v2.10.14 [49].

The phylogenetic tree clustered each isoform distinctly, with *P. knowlesi* Hsp90s grouping closely with *P. vivax* Hsp90s in all isoforms, and *P. berghei* proteins grouping with *P. yoeli* in ER and mitochondria isoforms, consistent with known evolutionary proximity [46] (Figure 4). Arising off the clusters or in-between are the *P. falciparum* Hsp90 isoforms, suggesting a slightly greater evolutionary distance [46,47]. An even more distant relationship with human Hsp90 isoforms correlated with the lower level of sequence similarity and structural differences that could be exploited for drug intervention (Figure 4). It should be noted that human ER HSPC4 clustered closer to the cytosolic Hsp90 isoforms instead of the ER Hsp90 isoforms but arose from the same root. To probe this further, two additional apicomplexan Hsp90 proteins (from *Theileria anniculata* and *Babesia ovata*), as well as a chloroplast Hsp90 from *Nicotiana tabacum*, were included in the phylogenetic analysis. The results showed that the two additional apicomplexan Hsp90s shared similar clustering with the plasmodial isoforms (Figure 4). Interestingly, HSPC4 appears to be evolutionarily closer to chloroplast Hsp90 and may serve as an intermediate between cytosolic and ER Hsp90 isoforms.

### 2.3. Functional Annotation and Protein–Protein Interaction (PPI) Analysis

Network analysis was conducted with the four identified *P. knowlesi* Hsp90 isoforms in comparison with *P. falciparum* Hsp90 isoforms using STRING v11 [50,51,52] and ShinyGO v0.82 [53] online resources. While all the four *P. falciparum* Hsp90 isoforms were successfully mapped by STRING v11, only three (PKNH_0915900/PkTRAP1, PKNH_1238400/PkHsp90_A, and PKNH_1441400/PkGRP94) were successfully mapped in *P. knowlesi* (Tables S2 and S3). The Gene Ontology (GO) enrichment analysis revealed similar biological processes and molecular functions associated with all the Hsp90 isoforms in *P. falciparum* and *P. knowlesi* (Table 1). Of the top 20 GO-enriched terms associated with *P. falciparum* Hsp90 proteins, 12 can be identified with *P. knowlesi* Hsp90 isoforms. With all the GO functional terms associated with the three mapped *P. knowlesi* Hsp90s also found in the four mapped *P. falciparum* Hsp90s, it can be inferred that the unmapped *P. knowlesi* protein (PKNH_0107000/PkHsp90) will possess similar functional terms with its corresponding *P. falciparum* ortholog. Meanwhile, GO annotation indicated very strong involvement in biological processes, including protein folding (GO:0006457), response to stress (GO:0006950), and ATPase activities (GO:0016887 and GO:0140657), as well as molecular functions, such as, unfolded protein binding (GO:0051082), ATP binding (GO:0005524), adenyl ribonucleotide binding (GO:0032559), and adenyl nucleotide binding (GO:0030554) (Table 1 and Figure S6). These functions are critical during parasite development in the febrile host environment, particularly during schizogony and erythrocyte invasion. The prominent GO cellular components identified were sarcoplasmic reticulum lumen (GO:0033018), melanosome (GO:0042470), and the perinuclear region of the cytoplasm (GO:0048471). These are associated with Hsp90 orthologs in apicoplast (PKNH_1238400/PkHsp90_A and PF3D7_1443900/PfHsp90_A) and ER (PKNH_1441400/PkGRP94 and PF3D7_1222300/PfGRP94) in *P. knowlesi* and *P. falciparum*, suggesting similar roles for *P. knowlesi* Hsp90s.

**Figure 5:**
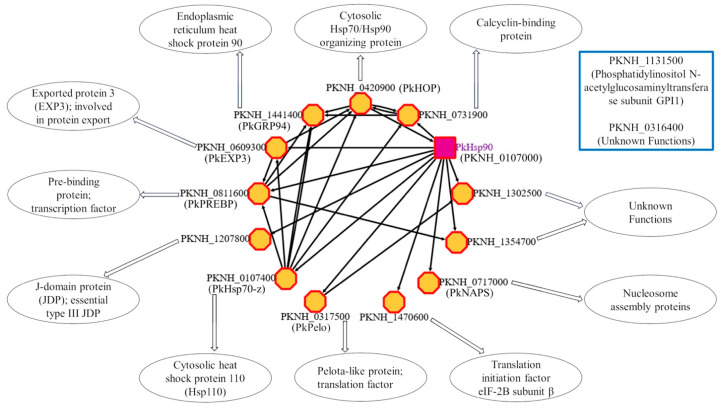
Protein network interaction scheme of cytosolic PkHsp90. Target nodes are represented as octagons bordered in red and filled in yellow for all predicted interacting proteins. The source node, cytosolic PkHsp90, is shown as a rectangle bordered in red and filled in purple. Arrows (black) point from the source to the target nodes and serve as indicators of predicted functional interactions. In brackets are other known common names. Shown in a blue-bordered rectangular box are the BioGrid-identified interacting partners that are not found in the STRING v11 database, where the network was generated. The network was rendered using Cytoscape v3.10.4 [57,58]. Known putative functions are shown in oval shapes pointed by block arrows [59,60,61,62,63,64,65,66,67,68]. Images were prepared using PowerPoint and GIMP v2.10.14 software [49].

In addition, we predicted a STRING-based PPI network for cytosolic PkHsp90 (Figure 5) using the interacting partners of cytosolic PfHsp90 (Figure S7) and their orthologs in *P. knowlesi* as templates (retrieved from PlasmoDB [54] and confirmed with BLASTp searches [55]). The PkHsp90 interaction network revealed expected predicted interactions with certain key parasite-resident co-chaperone proteins previously reported for PfHsp90 [56], including PkHOP (PKNH_0420900) and a type III J-domain protein (JDP; also called Hsp40) (PKNH_1207800). The predicted interactions with a number of other parasite-resident proteins aligned well with known Hsp90 chaperone functions associated with important intracellular processes, such as the regulation of transcription (e.g., the transcription factor pre-binding protein, PkPREBP/PKNH_0811600), translation (e.g., the translation factor pelota-like protein, PkPelo/PKNH_0317500), and protein folding and the stress response (e.g., the cytosolic Hsp110, PkHsp70-z/PKNH_0107400) (Figure 5). Furthermore, the PkHsp90 network appears to extend beyond the boundaries of the parasite, suggesting an association with components of the protein export machinery (e.g., exported protein 3, PkEXP3/PKNH_0609300) (Figure 5).

### 2.4. Sequence and Structural Analyses of the ATP Binding Pockets and Lid Domains

**Figure 6:**
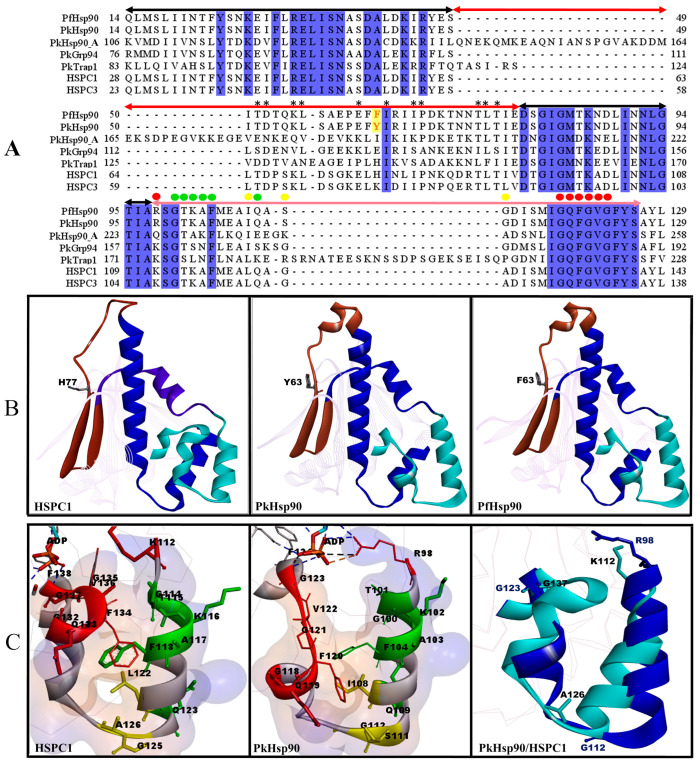
Comparative analyses of the ATP-binding pockets and lid domains of human and plasmodial Hsp90s, highlighting that the GHL has a straight conformation in PkHsp90 forming a hydrophobic extension, which was not observed for human HSPC1 where the GHL was curved. (**A**) Sequence analysis: Highlighted with double-faced arrows are ATP/ADP binding regions in black, regions of low sequence similarity in red, and lid domains of plasmodial (PfHsp90 and *P. knowlesi* Hsp90 isoforms) as compared with human Hsp90s (HSPC1 and HSPC3) in rose. Sequences within the low similarity region that are identical in PfHsp90, PkHsp90, HSPC1, and HSPC3 are identified with asterisks. A key amino acid residue difference between PfHsp90 (F63) and PkHsp90 (Y63) is highlighted in red text on a yellow background. Blue background colour indicates sequence identity and conservation with a threshold of not less than 100%. Amino acids identified with coloured balls on the alignment represent glycine-rich hinge loop (GHL, red), N-terminal α-helix of the lid domain (green), and the characteristic IXXSG and LXXGA motifs of cytosolic plasmodial and human Hsp90s, respectively (yellow). (**B**) Functional loops analysis: Loops are rendered as solid ribbons coloured cyan for the lid domains, brown for the regions of low sequence similarity, and blue for the ATP/ADP interacting loop. A key amino acid residue difference between PfHsp90 (F63) and PkHsp90 (Y63) and the corresponding HSPC1 residue (H77) are rendered as sticks coloured by element. (**C**) Structural analysis: For HSPC1 and PkHsp90, the lid domains are rendered as solid grey ribbons, with the GHL motifs in red, the N-terminal α-helix of the lid domains in green, and IXXSG and LXXGA motifs in yellow, as previously reported [30]. Some corresponding amino acids are rendered as sticks with parent colour. Other regions are rendered as Ca wire in light grey. Hydrophobicity is rendered as transparent surfaces, with a colour range from blue (most hydrophilic) to orange (most hydrophobic). PkHsp90/HSPC1 represents the superimposition of the lid domains of PkHsp90 (blue) and HSPC1 (cyan), with some corresponding amino acids rendered as sticks with parent colour. HSPC1 (1BYQ) and PfHsp90 (3K60) are the experimentally determined structures of the NTDs co-crystallized with ADP. PkHsp90 is the modelled NTD with ADP (PKNH_0107000). Images were prepared using Discovery Studio Visualizer v25.1.0.24264 [72], arranged on PowerPoint, and processed with GIMP v2.10.14 software [49].

A structural analysis of the ATP binding pocket and lid domain of PkHsp90 in comparison with those of PfHsp90 and human Hsp90 (HSPC1) was conducted, with the aim of identifying structural and functional similarities and differences that may be applicable in drug targeting. The lid domain consists of the GHL motif, a N-terminal α-helix, and the characteristic IXXSG (plasmodial) and LXXGA (human) motifs located in the cap region. To this end, a model of the NTD of PkHsp90 was generated using the SWISS MODEL server [69,70]. The model showed good quality, with a GMQE score of 0.82, MolProbity score of 0.83, Clash score of 0.30, a high Ramachandran favoured percentage (96.62%), no Ramachandran and Rotamer outliers (0.00%), and a good QMEANDisCo Global score (0.80 ± 0.06). Also, to assess the degree of sequence conservation, a multiple sequence alignment of the ATP binding pockets and lid domains was conducted for all *P. knowlesi* Hsp90 isoforms, cytosolic PfHsp90, and the human cytosolic isoforms (HSPC1 and HSPC3) using Jalview v2.11.5.0 [48] (Figure 6A). The cytosolic PkHsp90 and PfHsp90 showed >97% sequence similarity between Q14 and S49 and between D79 and L129 with the human Hsp90 isoforms. These are the regions containing residues that interact with known NTD ligands of cytosolic Hsp90s. From I50 to E78, there are increasing substitutions with different amino acids in the human Hsp90 isoforms (Figure 6A). Further structural analysis of the NTDs revealed three loops (Figure 6B). The lid domains (R98–L129; Figure 6B, cyan) and ATP interacting pockets (Q14–S49 and D79–A97; Figure 6B, blue) are known to be functionally relevant as many known inhibitors interact with residues within these loops [24,30,71]. The third loop (Figure 6B, brown) is the joining loop of the ATP interacting pocket and has very low levels of sequence similarity. Specifically, L64/L59 to V92/V88 of HSPC1/HSPC3 are essentially different from the plasmodial cytosolic Hsp90s (I50 to E78), showing about 34% sequence similarity (Figure 6A,B). Also, F63 in PfHsp90 is replaced with Y63 in PkHsp90 (Figure 6A), providing a potential target for selective inhibition of PkHsp90. When compared with other *P. knowlesi* Hsp90 isoforms, this region in PkHsp90 also showed low sequence similarity. Therefore, this region may constitute a novel loop that could be exploited for isoform-selective drug targeting. Interestingly, both ADP and GDM do not show any interaction with residues of this loop in both human and plasmodial cytosolic Hsp90s (Figure 6B).

Comparative structural analysis of the lid domain of cytosolic PkHsp90 revealed that the GHL motif had a straight conformation that formed a hydrophobic extension or pocket, which was not observed for human HSPC1 where the GHL was curved (Figure 6C). This key difference was highlighted when the lid domains of PkHsp90 and HSPC1 were superimposed on one another (Figure 6C). Furthermore, in HSPC1 (1BYQ), ADP did not show any interaction with K112 or the closely linked G136 and G137, despite interacting with F138. On the other hand, in PkHsp90, ADP did show an interaction with R98 (equivalent to K112 in HSPC1). Cytosolic PfHsp90 has previously been shown to have a hydrophobic extension of the lid domain [30]; hence, the findings for PkHsp90 reinforce the existence of a unique hydrophobic pocket in the lid domains of cytosolic plasmodial Hsp90s. Furthermore, while most of the residues in the lid domains are conserved in the *P. knowlesi* Hsp90 isoforms, there are certain key differences between PkHsp90 and the other isoforms (e.g., R98 in PkHsp90 was replaced with K160, Q226 and K174 in PkGRP94, PkHsp90_A and PkTRAP1, respectively) that are likely to cause the plasmodial-specific hydrophobic pocket within the lid domains to have different structural features. To further interrogate the observed differences in the lid domains of *P. knowlesi* Hsp90 isoforms, a comparative structural analysis of the lid domains in all isoforms was conducted using their full-length predicted structures (Figure S8). Although they show similar structural features, PkHsp90 appears to be in a more open conformation then the other isoforms. Also, with a longer insertion (S188–Q210), the lid domain of PkTRAP1 was more hydrophilic compared to the other isoforms. PkHsp90 is the most hydrophobic, followed by PkGRP94 and PkHsp90_A. The increasing change toward hydrophilicity from PkHsp90 to PkGRP94, followed by PkHsp90_A to PkTRAP1, may be an indication of potential functional differences.

### 2.5. Comparative Docking Analysis of the ATP Binding Pocket and Lid Domains

**Figure 7:**
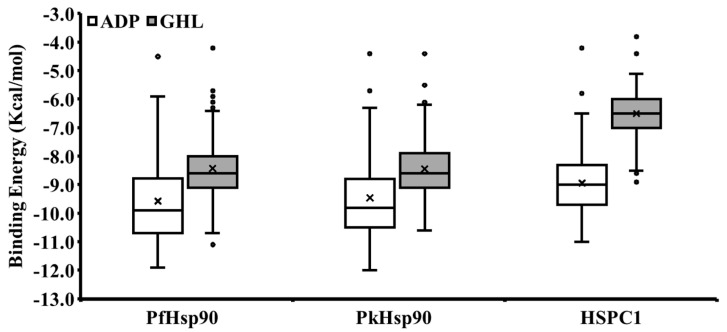
Box and whisker plot of virtual screening of small molecule inhibitors against the ADP-binding pockets and lid domains of plasmodial and human Hsp90s. Plots with filled and unfilled boxes indicate ligand interaction with lid domains (GHL) or ADP-binding pockets (ADP), respectively. The black dots represent data points that fall outside the main data distribution range, and the crosses represent the average of the data. Human HSPC1 (1BYQ) and PfHsp90 (3K60) are the experimentally determined NTDs co-crystallized with ADP. PkHsp90 is the modelled NTD with ADP (PKNH_0107000). Image was prepared on Microsoft Excel and processed with GIMP v2.10.14 software [49].

The modelled NTD in complex with ADP for PkHsp90 and the experimentally determined NTDs co-crystallized with ADP of PfHsp90 (PDB: 3K60) and human HSPC1 (PDB: 1BYQ) were used to assess the binding potentials of our local database of PfHsp90 small molecule inhibitors. The DoGSiteScorer v2.0.0 predicted multiple druggable pockets on the PkHsp90 model, with a high druggability score (0.81) comparable to PfHsp90 (3K60: 0.83) and HSPC1 (1BYQ: 0.83). The ADP-binding site is a highly druggable site, with ligand coverage of greater than 90% in all protein complexes. A comparative protein–ligand interaction analysis was conducted on the three proteins with ADP and GDM (Figure S9). The interaction analysis revealed that PkHsp90 and PfHsp90 shared some amino acids in common, such as A41, K44, D79, M84, and R98. It also revealed hydrogen bonding with critical ADP binding cleft residues, like D79, M84, and R98. Importantly, while human HSPC1 shared hydrogen bonding with two equivalent residues of the plasmodial Hsp90s (D93 and M98 equivalent to D79 and M84), one critical contact was missing (K112 equivalent to R98), as alluded to in the previous section. The lid domain has been reported to possess the potential for highly selective targeting of PfHsp90 in antimalarial drug discovery [29,30], and structural analyses in this report suggest that the lid domain of PkHsp90 could also be selectively targeted. In this report, we conducted virtual screening of our local database of small molecule inhibitors against the ADP-binding pocket and lid domains of PkHsp90, PfHsp90, and HSPC1 (Table S4). The ranked scores of the interactions of each ligand with the proteins revealed their potential for possible preferential functional binding with the plasmodial Hsp90 proteins. Specifically, the ranking showed that all the small molecule compounds considered in this study had very weak or no binding affinity with the lid domain of HSPC1, suggesting selective targeting of the plasmodial Hsp90s. Further analysis revealed that of the top-rated consensus ADP-binding site interacting molecules, only two out of ten bound to HSPC1 (Table 2). Similarly, of the top-rated lid domain interacting molecules, only four compounds bound to HSPC1 (Table 2). To further evaluate this potential, we constructed a box and whisker plot of the screening scores (Figure 7). The plot confirmed the preferential binding to the plasmodial Hsp90s over HSPC1, suggesting that these small molecule compounds might be useful leads in drug discovery efforts against *P. knowlesi* malaria. It should be noted, however, that although some level of selectivity might be achieved with the ADP-binding site, interaction with the lid domains showed a distinctive selective interaction with PkHsp90 and PfHsp90, indicating a higher potential for selective targeting.

**Figure 8:**
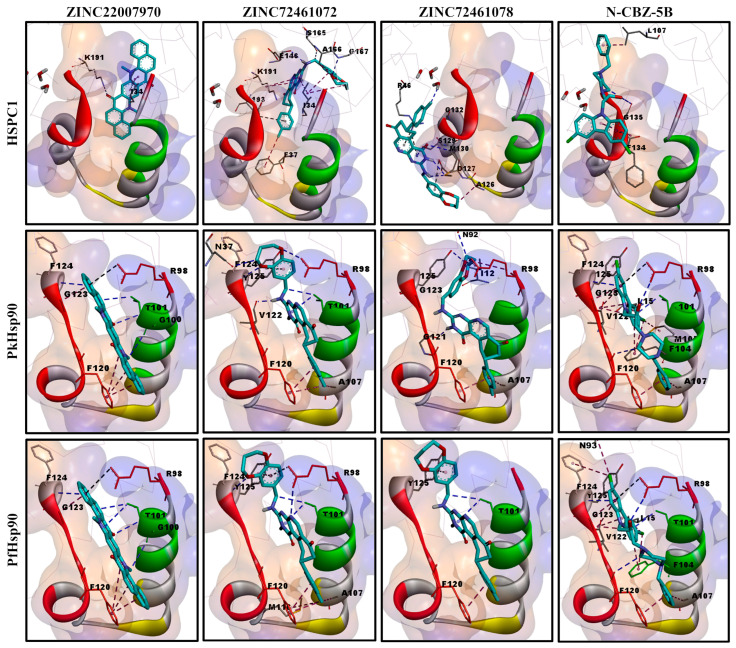
Docking pose and interaction analysis of top-rated compounds with the lid domains of HSPC1, PkHsp90, and PfHsp90. The lid domains are shown as solid grey ribbons, with the amino acids typifying critical motifs coloured red (GHL), green (N-terminal α-helix), and yellow (IXXSG in PkHsp90/PfHsp90 and LXXGA in HSPC1), as previously reported [30]. All other regions are rendered as Ca wire in light grey to allow for visibility of the interacting residues. The docked compounds are rendered as sticks and coloured by atoms with carbon as cyan, chloride as green, nitrogen as blue, and oxygen as red. Interacting residues are represented as sticks with parent colours. Broken lines represent hydrogen bond interactions (blue), hydrophobic interactions (purple), and electrostatic interactions (black). Hydrophobicity is rendered as transparent surfaces with a colour range from blue (most hydrophilic) to orange (most hydrophobic). Human HSPC1 (1BYQ) and PfHsp90 (3K60) are the experimentally determined NTDs co-crystallized with ADP. PkHsp90 is the modelled NTD with ADP (PKNH_0107000). Images were prepared using Discovery Studio Visualizer v25.1.0.24284 [72], arranged on PowerPoint, and processed with GIMP v2.10.14 software [49].

Furthermore, we performed a comparative analysis of the docking pose and ligand interactions of the top-rated ZINC compounds (ZINC22007970, ZINC724661072, and ZINC724661078) in comparison with N-CBZ-5B, an amino-alcohol carbazoles compound, which was previously identified as a unique binder of the lid domain of PfHsp90 [29,30] (Figure 8). The compounds showed little (ZINC724661078 and N-CBZ-5B) or no (ZINC22007970 and ZINC724661072) interactions within the lid domain of HSPC1. Those that showed low affinity interaction with HSPC1 did not interact with the critical residues (e.g., K112). However, for PkHsp90 and PfHsp90, all the compounds docked within the lid domain and showed interaction with the critical residues, sharing many residues with N-CBZ-5B, including R98, G100, T101, A107, F120, V122, and G123. The interaction analysis also showed that PkHsp90 and PfHsp90 shared similar interacting residues within the lid domains. Therefore, it can be inferred that PkHsp90 is structurally and functionally amenable to inhibition by small molecule compounds known to bind to plasmodial Hsp90s, with the potential for lead optimization based on its unique structural features.

## 3. Discussion

This study is the first comprehensive bioinformatic analysis to provide structural and functional insight into the Hsp90 proteins of *P. knowlesi*, with a view to assessing their suitability for selective drug targeting. While the cytosolic PkHsp90 interaction network revealed the expected predicted interactions with certain key parasite-resident co-chaperones (PkHOP and a type III JDP) [56], other predicted interactions were less expected, but nevertheless consistent with the role of the major molecular chaperones in proteostasis, such as the regulation of transcription (e.g., PkPREBP) [65], translation (e.g., PkPelo) [67], and protein folding and the stress response (e.g., PkHsp70-z) [59,60,61]. Interestingly, the PkHsp90 network appears to include proteins beyond the boundaries of the parasite (e.g., PkEXP3) [63].

While the predicted structures of all the *P. knowlesi* Hsp90 isoforms shared similar overall 3D domain architectures as the human cytosolic Hsp90s (HSPC1 and HSPC3), key differences were identified (e.g., the longer charged linker region of cytosolic PkHsp90 and a large insertion in the NTD of apicoplast PkHsp90_A). Protein sequence and structural analyses, protein–ligand interaction, and small molecule docking studies revealed that cytosolic PkHsp90 harbours distinct druggable features that differ from human HSPC1, suggesting the feasibility for species-selective inhibitor development. In particular, we have confirmed the presence of a second plasmodial-specific GHL-associated hydrophobic pocket extending from the ATP-binding pocket, which was not observed for human HSPC1. Virtual screening against this pocket, using a database of small molecules known or predicted to inhibit PfHp90, revealed preferential small molecule binding to PkHsp90 over human HSPC1. This virtual screening identified for the first time a number of compounds from the ZINC database (ZINC22007970, ZINC724661072, and ZINC724661078) that bound to the GHL-associated pocket of PkHsp90, with greater apparent affinity than previously identified amino-alcohol carbazoles [30]. While targeting the ATP pocket remains a valid option, our virtual screening suggests that greater selectivity for plasmodial Hsp90s over human Hsp90s can be achieved by targeting the GHL-associated pocket. In addition, we have highlighted a key loop in PkHsp90 and PfHsp90 (I50 to E78) that has very low levels of sequence similarity with HSPC1. While the GHL-associated pocket lies downstream of the ATP pocket, this loop connects the two coils that make up the residues interacting with ATP. This loop also harbours the replacement of F63 in PfHsp90 with Y63 in PkHsp90, suggesting its potential for selective targeting of PkHsp90. However, further analysis will be needed to ascertain the functional and drug targeting potential of this loop.

## 4. Materials and Methods

### 4.1. Sequence Retrieval and Identification of Hsp90 Isoforms

The protein sequences of *P. falciparum* Hsp90 family members were retrieved from the PlasmoDB [54,73,74] and NCBI protein databases [75]. The BLASTp searches [55] in the PlasmoDB platform were conducted using the retrieved known *P. falciparum* Hsp90 sequences as queries to identify orthologs in *P. knowlesi*, *P. vivax*, *P. berghei*, and *P. yoeli*. Identified sequences were validated based on conserved Hsp90 domain architecture. Domain annotation was conducted using Interpro online resource [42,43]. With respect to the human Hsp90 sequences, the HSPC nomenclature proposed by Kampinga et al. (2009) [76] was adopted, where HSPC1 (gene ID 3320) refers to the inducible cytosolic isoform (also called Hsp90α), HSPC3 (gene ID 3326) refers to the constitutively expressed cytosolic isoform (also called Hsp90β), HSPC4 (gene ID 7184) refers to the ER isoform (also called GRP94), and HSPC5 (gene ID 10131) refers to the mitochondrial isoform (also called TRAP1). Also retrieved, were the control proteins included in the phylogenetic analysis, namely, TaHsp90 (TA12105), TaTRAP1 (TA06845), TaGRP94 (TA06470), and TaHsp90_A (TA10720) from *Theileria anniculata*; BoHsp90 (BOVATA_007200), BoTRAP1 (BOVATA_017520), BoGRP94 (BOVATA_020650), and BoHsp90_A (BOVATA_003160) from *Babesia ovata*; as well as NtHsp90 (LOC107797756), the chloroplast Hsp90 from *Nicotiana tabacum*. The complete list of all proteins used in this study and their Uniprot and NCBI identifiers are contained in Table S5.

### 4.2. Three-Dimensional Protein Structure Retrieval and Modelling

Where indicated, the three-dimensional (3D) structures of proteins for alignment and docking were retrieved from the RCSB PDB or AlphaFold databases [77,78]. The following proteins were retrieved: NTD of human Hsp90 bound to GDM (PDB: 1YET) and ADP (PDB: 1BYQ); full-length human Hsp90 bound to ATP (PDB: 5FWK); NTD of PfHsp90 bound to ADP (PDB: 3K60); cytosolic PkHsp90 (PKNH_0107000; AlphaFold model: A0A679KRE8), ER PkGRP94 (PKNH_1441400; AlphaFold model: A0A384LP92), apicoplast PkHsp90_A (PKNH_1238400; AlphaFold model: A0A384K893), and mitochondrial PkTRAP1 (PKNH_0915900; AlphaFold model: A0A384K8H3). The modelling for the full-length Hsp90 dimers was conducted with SWISS-MODEL [69,70] (SWISS-MODEL: homology modelling of protein structures and complexes; available online: https://swissmodel.expasy.org/; accessed on 7 October 2025; homodimer mode), taking 5FWK, 8FFV, 7Y04 (electron microscopic structures), 5ULS, 4YIN, and 5ULS (X-ray crystallographic structures), respectively, as templates. For the detailed structural analysis and docking studies of the ATP-binding site and associated lid domain, a model of the NTD of PkHsp90 was generated using SWISS-MODEL server, with chain A of the NTD of PfHsp90 bound to ADP (PDB: 3K60) as template (97.16% sequence coverage). The NTD PkHsp90–ADP complex model was generated via superimposition with the template, as earlier described [24].

### 4.3. Multiple Sequence and Structural Alignments and Phylogenetic Analysis

The retrieved Hsp90 sequences from 4.1 above were aligned using the mcoffee mode of TCoffee v13.46.2.7c9e712d [79] and Clustal Omega v1.2.4 [80,81,82] as implemented in UniProt online resource [83]. The mcoffee mode of TCoffee computes a consensus sequence alignment from separate alignments obtained from ClustalW v2.0 [84], TCoffee v13.46.2.7c9e712d [79], abPOA v1.5.5 [85], Muscle v5 [86], Mafft v7.526 [87], DIALIGN-T v1.0.2-15 [88], PCMA v2.0 [89], and ProbCons v1.12 [90]. The alignments were conducted on the full-length sequences, checked manually, and annotated using Jalview v2.11.5.0 [48]. The Clustal Omega v1.2.4 from the Uniprot resource was used to compute pairwise percentage identity using the full-length sequences. Protein structure alignments were performed using TM-Align as implemented in the RCSB PDB online resource [77,91]. Structural alignment of each AlphaFold downloaded structure was performed with multiple control PDB structures (1YET, 5FWK and 3K60). The aligned structures were viewed using Discovery Studio Visualizer v25.1.0.24284 [72]. Phylogenetic trees were constructed using the NGPhylogeny.fr online resource [92] with its PhyML/OneClick default settings, involving sequence alignment using Muscle v5 [86], curation to eliminate poorly aligned positions and divergent regions using Gblocks 0.91b, phylogeny using PhyML v3.1/3.0 aLRT, and tree rendering using TreeDyn 198.3 [92]. Phylogenetic tree annotated was conducted using the iTOL v6 online resource [93]. Final image processing was conducted with GIMP v2.10.14 [49,94].

### 4.4. Protein–Protein Interaction (PPI) Network and Functional Analysis

Protein interaction networks were constructed for *P. knowlesi* and *P. falciparum* Hsp90 protein families using the STRING v11 database [50,52]. High-confidence (0.70) interactions within the query proteins and not more than 10 additional proteins as determined by the STRING v11 database were selected based on all active interaction sources (text-mining, experiments, databases, co-expression, neighbourhood, gene fusion, and co-occurrence) for the predictions. Protein interaction networks for known interacting partners of PfHsp90 were retrieved from the BioGRID database [95,96]. The protein sequence of each of the retrieved PfHsp90 partners was used as a query sequence for BLASTp searches [55] in the PlasmoDB platform [54] to identify orthologs in *P. knowlesi*. Functional networks of PfHsp90 and associated partners, as well as those of the identified orthologs of *P. knowlesi*, were constructed using the STRING v11 database [50,52]. The two networks were used as templates for the construction of a predicted network of PkHsp90 with identified orthologs. Functional annotations were inferred using Gene Ontology (GO) terms and KEGG pathway enrichment analysis through ShinyGO v0.82 online resource [53].

### 4.5. Sequence and Structural Analyses of the ATP-Binding Pocket and Lid Domains

The structural analysis of the ATP-binding pocket and lid domain of PkHsp90 was performed in comparison with those of PfHsp90 and human Hsp90 (HSPC1/Hsp90α) as previously reported [30]. Multiple sequence alignment of the NTD sequence was conducted for all *P. knowlesi* Hsp90 isoforms, HSPC1 (Hsp90α), HSPC3 (Hsp90β), and PfHsp90 using the mcoffee mode of TCoffee v13.46.2.7c9e712d [79], as detailed in Section 4.3. The alignment was checked manually and annotated using Jalview v2.11.5.0 [48]. The structural features of the ATP-binding pockets and lid domains (consisting of the GHL motif, the N-terminal α-helix of the lid domains, and the characteristic IXXSG and LXXGA motifs located in the cap of the lid domains of plasmodial and human Hsp90s, respectively) were generated for analysis using Discovery Studio Visualizer v25.1.0.24284 [72].

### 4.6. Drug Target Analysis and Ligand Docking

Ligands reported to have potential for interaction with the plasmodial Hsp90 were sourced from the literature [27,31,97,98,99] to create a local database. The 3D structures of ligands were either downloaded from the ZINC database [100,101] or PubChem [75]. Druggability of the modelled complex was evaluated using DoGSiteScorer v2.0.0 [102,103]. Preparation of protein structures and ligands for docking using the VEGA ZZ v3.2.4.29 platform [104] and the validation of the docking procedure were performed as previously reported [24]. Two controls—the experimentally determined NTDs co-crystallized with ADP of HSPC1 (PDB: 1BYQ) and PfHsp90 (PDB: 3K60) and the test, the modelled NTD of PkHsp90 with ADP (PKNH_0107000)—were used for screening against the database of sourced Hsp90 inhibitors. Molecular docking was conducted, targeting the ATP-binding pocket and lid domain binding pocket, using AutoDock Vina v1.2.7 [105]. For each docking interaction, exhaustiveness and binding modes were set at 8 and 10, respectively, leading to the generation of ten poses per ligand. Docking interaction scores were analyzed for the identification of the top-rated interacting compounds of PkHsp90 in comparison with PfHsp90 and HSPC1. The coordinates (PDB files) for the protein structures used in the docking (PkHsp90, PfHsp90, and HSPC1) and the coordinates (SDF files) for the top-rated poses of the compounds (ZINC22007970, ZINC724661072, ZINC724661078, and N-CBZ-5B) are lodged in the Supplementary Materials. Also, a comparative analysis of the scores for the identification of selective inhibition was performed using box and whisker plots. Binding affinities and interaction profiles were analyzed to assess the potential for selective inhibition using Discovery Studio Visualizer v25.1.0.24284 [72].

## 5. Conclusions

In conclusion, this study represents the first comprehensive bioinformatics analysis of the Hsp90 isoforms of *P. knowlesi*, a zoonotic malaria parasite of growing clinical concern. Through sequence analysis, domain annotation, phylogenetic analysis, network analysis, protein–ligand interaction, and docking simulations, the predicted structure, function, and druggability of the PkHsp90s were clearly revealed. These findings will inform the development of hypotheses for future experimental testing of the role of this important family of proteins in knowlesi malaria. All isoforms demonstrated high conservation with other *Plasmodium* species, supporting their fundamental role in parasite survival and stress adaptation, while the network analysis revealed that these chaperones played a role in key cellular processes within and beyond the parasite, making them attractive multi-pathway targets. The findings from the molecular docking and virtual screening are very promising; however, further experiments are required to validate the novel potential PkHsp90 inhibitor compounds, through biochemical assays (using recombinant protein, ATPase, and chaperone activity inhibition assays), parasite growth inhibition assays, and cytotoxicity profiling using human cell lines. Given the adaptability of *P. knowlesi* and its potential for severe human infections, characterizing and targeting essential stress response proteins like Hsp90 offers a promising route for antimalarial drug development.

## Figures and Tables

**Table 1:** Comparative assessment of the Gene Ontology enrichments associated with mapped *P. falciparum* and *P. knowlesi* Hsp90 proteins.

*P. falciparum* Enrichment		GO Terms	Description	*P. knowlesi* Enrichment		Functional Terms
FDR	Fold			FDR	Fold	
2.04 × 10^−7^	83.72	GO:0051082	unfolded protein binding	1.65 × 10^−6^	255.10	MF
1.06 × 10^−6^	52.53	GO:0006457	protein folding	1.03 × 10^−5^	113.38	BP
2.39 × 10^−6^	41.86	GO:0016887	ATP hydrolysis activity	2.97 × 10^−5^	63.78	BP
1.31 × 10^−5^	26.26	GO:0017111	nucleoside-triphosphatase activity	9.59 × 10^−5^	36.71	BP
1.48 × 10^−5^	24.69	GO:0006950	response to stress	2.31 × 10^−5^	76.15	BP
1.48 × 10^−5^	24.24	GO:0016462	pyrophosphatase activity	9.59 × 10^−5^	34.24	BP
1.48 × 10^−5^	23.81	GO:0016818	hydrolase activity acting on acid anhydrides in phosphorus-containing anhydrides	9.59 × 10^−5^	33.35	BP
1.48 × 10^−5^	23.71	GO:0016817	hydrolase activity acting on acid anhydrides	9.59 × 10^−5^	33.13	BP
3.71 × 10^−5^	18.6	GO:0140657	ATP-dependent activity	0.00022	24.30	BP
0.00018	12.32	GO:0005524	ATP binding	0.00103	13.43	MF
0.00021	11.67	GO:0032559	adenyl ribonucleotide binding	0.00103	13.36	MF
0.00021	11.62	GO:0030554	adenyl nucleotide binding	0.00103	13.32	MF
0.00029	10.26	GO:0035639	purine ribonucleoside triphosphate binding	0.00122	11.19	MF
0.00032	9.8	GO:0032555	purine ribonucleotide binding	0.00122	11.14	MF
0.00032	9.74	GO:0017076	purine nucleotide binding	0.00122	11.12	MF
0.00032	9.71	GO:0032553	ribonucleotide binding	0.00122	11.04	MF
0.00032	9.64	GO:0050896	response to stimulus	7.92 × 10^−5^	42.87	BP
0.00033	9.43	GO:0097367	carbohydrate derivative binding	0.00122	10.93	MF
0.00033	9.4	GO:0043168	anion binding	0.00131	10.50	MF
0.00039	8.8	GO:1901265	nucleoside phosphate binding	0.00206	8.77	MF
0.00039	8.8	GO:0000166	nucleotide binding	0.00206	8.77	MF
0.00043	8.52	GO:0036094	small molecule binding	0.00213	8.55	MF
0.00062	7.71	GO:0016787	hydrolase activity	0.00122	11.52	BP
0.00107	6.63	GO:0005515	protein binding	0.00118	12.44	MF
0.00153	6.02	GO:0043167	ion binding	0.00396	6.86	MF
0.00519	4.34	GO:1901363	heterocyclic compound binding	0.00671	5.61	MF
0.00519	4.33	GO:0097159	organic cyclic compound binding	0.00671	5.61	MF
0.01267	3.43	GO:0003824	catalytic activity	0.01484	4.25	BP
0.03449	2.55	GO:0005488	binding	0.02542	3.49	MF
0.07697	2.01	GO:0009987	cellular process	0.02542	3.47	BP
0.10566	1.82	GO:0003674	molecular function	0.08176	2.30	MF
0.10637	1.81	GO:0008150	biological process	0.03437	3.11	BP

Mapped genes: PfHsp90 (PF3D7_0708400), PfTRAP1 (PF3D7_1118200), PfGRP94 (PF3D7_1222300), PfHsp90_A (PF3D7_1443900), PkTRAP1 (PKNH_0915900), PkGRP94 (PKNH_1441400), and PkHsp90_A (PKNH_1238400); BP, biological process; MF, molecular function; GO, Gene Ontology; FDR, False Discovery Rate.

**Table 2:** Top consensus interactors of the ADP-binding pocket and lid domain after virtual screening.

Compounds	ADP Binding Pocket			Lid Domain		
	PfHsp90	PkHsp90	HSPC1	PfHsp90	PkHsp90	HSPC1
N-CBZ-3A	−8.9	−9.3		−8.4	−8.5	−5.2
N-CBZ-3B	−8.7	−9.5		−9	−9.1	
N-CBZ-3C	−9	−8.9		−8.4	−8.2	
N-CBZ-3E	−9.2	−9.3		−8.5	−8.4	
N-CBZ-3G	−9.1	−9.1		−8.5	−8.5	
N-CBZ-5B	−8.9	−9		−8.8	−8.7	−6.2
SNX-2112	−9.7	−9.7	−9.2	−8.6	−8.2	−6.2
ZINC22007970	−11.4	−11.3		−10.5	−10.4	
ZINC72461072	−11.6	−11.4		−10.7	−10.4	−7.4
ZINC72461078	−11.6	−11.4	−11	−10.2	−10.3	

N-CBZ-* are amino-alcohol carbazoles compounds. Binding energies are in kcal/mol.

## Data Availability

All the data associated with this article can be found in the body of the article and in the Supplementary Materials.

## Supplementary Materials

The following supporting information can be downloaded at https://www.mdpi.com/article/10.3390/ijms262412065/s1. References [30,48,49,53,78] are cited in the Supplementary Materials.
