# Supplementary material for: Plasmodium knowlesi Heat Shock Protein 90s: In Silico Analysis Reveals Unique Druggable Structural Features
Source: Int J Mol Sci. 2025 Dec 15;26(24):12065. doi: 10.3390/ijms262412065 (PMC12732701; doi:10.3390/ijms262412065)
Supplement: Supplementary file 1 [file ijms-26-12065-s001.zip › ijms-4000428 supplementary final pdf.pdf]

International Journal of *Molecular Sciences*

Article

Plasmodium knowlesi heat shock protein 90s: *In silico* analysis reveals unique druggable structural features

Michael O. Daniyan[1], Harpreet Singh[2], and Gregory L. Blatch[3,4]*

[1] Department of Pharmacology, Faculty of Pharmacy, Obafemi Awolowo University, Ile-Ife 220005, Nigeria; mdaniyan@oauife.edu.ng; toyinpharm@gmail.com
[2] Department of Bioinformatics, Hans Raj Mahila Maha Vidyalaya, Jalandhar 144008, India; harpreet@bio-clues.org
[3] The Vice Chancellery, The University of Notre Dame Australia, Fremantle, WA 6959, Australia; greg.blatch@nd.edu.au
[4] Biomedical Biotechnology Research Unit, Department of Biochemistry, Microbiology and Bioinformatics, Rhodes University, Makhanda 6140, South Africa

* Correspondence: greg.blatch@nd.edu.au

SUPPLEMENTARY MATERIALS

Academic Editors: Nikolas Nikolaidis and Satish Raina

Received: 6 November 2025
Revised: 9 December 2025
Accepted: 11 December 2025
Published: 15 December 2025

Citation: Daniyan, M.O.; Singh, H.; Blatch, G.L. *Plasmodium knowlesi* Heat Shock Protein 90s: *In Silico* Analysis Reveals Unique Druggable Structural Features. *Int. J. Mol. Sci.* **2025**, *26*, 12065. https://doi.org/10.3390/ijms262412065

Copyright: © 2025 by the authors. Submitted for possible open access publication under the terms and conditions of the Creative Commons Attribution (CC BY) license (https://creativecommons.org/licenses/by/4.0/).

Figures S1-S9 and Tables S1-S5 are lodged as a single PDF file of the Supplementary Materials (legends/titles listed below)

Figure S1: Protein sequence alignment of selected plasmodial and human Hsp90s. Blue annotation indicates degree of conservation and sequence identity with a threshold of not less than 100%. Annotation was generated using Jalview (Waterhouse et al. 2009. Bioinformatics. 25: 1189–1191) and processed with GIMP software (Wolf. 2010. Galileo Press. 2010. ISBN 9783836216104).

Figures S2-S5: Protein sequence alignment of the cytosolic, apicoplast, endoplasmic reticulum and mitochondria isoforms of the selected Hsp90s. Blue annotation indicates degree of conservation and sequence identity with a threshold of not less than 100%. Annotation was generated using Jalview (Waterhouse et al. 2009. Bioinformatics. 25: 1189–1191) and processed with GIMP software (Wolf. 2010. Galileo Press. 2010. ISBN 9783836216104).

Figure S6: Comparative analysis of Gene Ontology enrichment analysis of (A) *P. knowlesi* and (B) *P. falciparum* Hsp90s. Annotation was generated using ShinyGO (Ge et al. 2019. Bioinformatics. 36: 2628-2629) and processed with GIMP software (Wolf. 2010. Galileo Press. 2010. ISBN 9783836216104).

Figure S7: Protein network interaction scheme of cytosolic PfHsp90. (A) The target nodes are represented as octagons bordered in red and filled in yellow for all interacting proteins. The source protein (PfHsp90) node is shown as a rectangle bordered in red and filled in purple. Arrows (black) point from source to the target nodes. In brackets are other known identifiers. Shown in a blue bordered rectangular box are the BioGrid identified interacting partners not found in the STRING database where the network was generated. (B) Known putative functions are shown in a table. The network was rendered using Cytoscape. Images were prepared using PowerPoint and GIMP software (Wolf. 2010. Galileo Press. 2010. ISBN 9783836216104).

Figure S8: Comparative analysis of the lid domain (associated with the ATP-binding site) of *Plasmodium knowlesi* Hsp90 isoforms. (A) Multiple sequence alignment of the lid domains of *Plasmodium knowlesi* Hsp90 isoforms. Blue background colour indicates sequence identity and conservation with a threshold of not less than 100%. Amino acids identified with coloured balls on the alignment represent the glycine-rich hinge loop (GHL, red), the N-terminal α-helix of the lid domain (green), and the characteristic IXXSG motif of the cytosolic plasmodial Hsp90s (yellow), respectively. (B) Structural analysis of the lid domains of *Plasmodium knowlesi* Hsp90 isoforms. Amino acids typifying the GHL motif, N-terminal α-helix and the IXXSG motif are represented as solid ribbons or loops in red, green and yellow, respectively, in line with a previous report (Wang et al. 2014. J. Med. Chem. 57: 2524–2535), and selected ones are further shown as sticks and labelled. Other protein regions are rendered as Ca wire with light grey colour to allow for visibility of the lid domains. Hydrophobicity is shown with a transparent surface with the colour ranging from blue (most hydrophilic) to orange (most hydrophobic). All the isoform structures used for this rendering were downloaded from the AlphaFold database (Jumper et al. 2021. Nature. 596: 583–589) as full-length proteins. Image was processed with GIMP software (Wolf. 2010. Galileo Press. 2010. ISBN 9783836216104).

Figure S9: Comparative protein-ligand interactions analysis at the ADP-binding site of plasmodial and human Hsp90s. The NTD of PfHsp90 (PDB: 3K60) with ADP (A) and Geldanamycin (B); the modelled NTD of PkHsp90 (PKNH_0107000; AlphaFold model: A0A679KRE8) with ADP (C) and Geldanamycin (D); and the NTD of HSPC1 (PDB: 1BYQ) with ADP (E) and Geldanamycin (F). Protein interacting residues and ligands are shown as sticks, coloured by element. Rendered as dash lines are hydrogen bonds (green), hydrophobic interactions (purple) and electrostatic interactions (purple).

Table S1: Pairwise Percentage Identity Matrix.
Table S2: The Gene Ontology Enrichment of *P. falciparum* Hsp90 isoforms.
Table S3: The Gene Ontology Enrichment of *P. knowlesi* Hsp90 isoforms.
Table S4: Virtual screening of small molecule inhibitors against the ADP-binding sites and associated lid domains in plasmodial and human Hsp90s.
Table S5: Plasmodial and human Hsp90s used in this study.

The coordinates (PDB and SDF Files) are lodged as a separate zip folder of the Supplementary Materials (descriptor below)
Coordinates (PDB and SDF Files): The zip folder contains the coordinates (PDB files) for the protein structures used in the docking (PkHsp90, PfHsp90, and HSPC1), and the coordinates (SDF files) for the top-rated poses of the top-rated compounds (ZINC22007970, ZINC724661072, ZINC724661078) and control compound (N-CBZ-5B).

Disclaimer/Publisher's Note: The statements, opinions and data contained in all publications are solely those of the individual author(s) and contributor(s) and not of MDPI and/or the editor(s). MDPI and/or the editor(s) disclaim responsibility for any injury to people or property resulting from any ideas, methods, instructions or products referred to in the content.

HUMAN AND PLASMODIAL HSP90 SEQUENCE ALIGNMENT

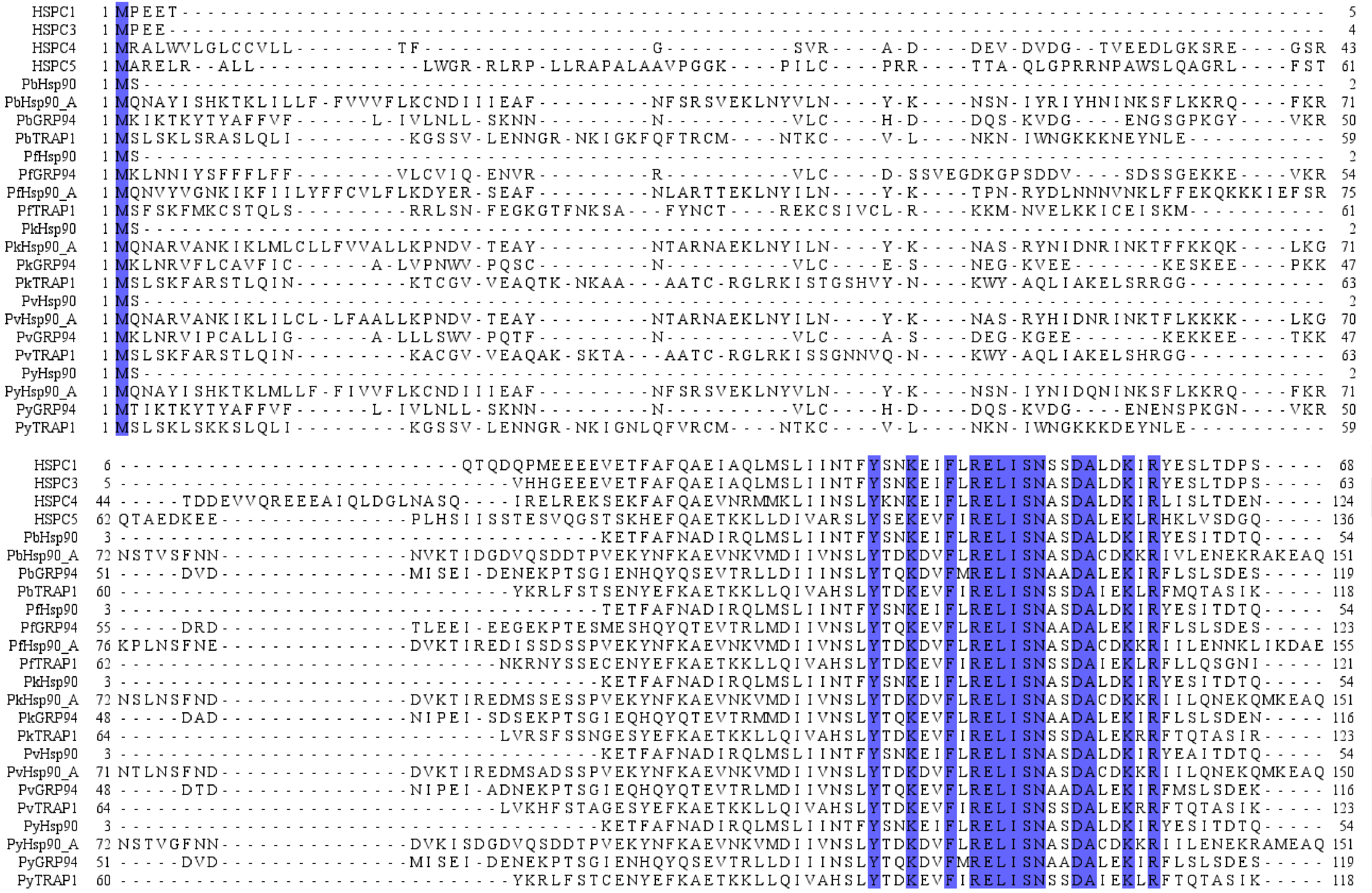

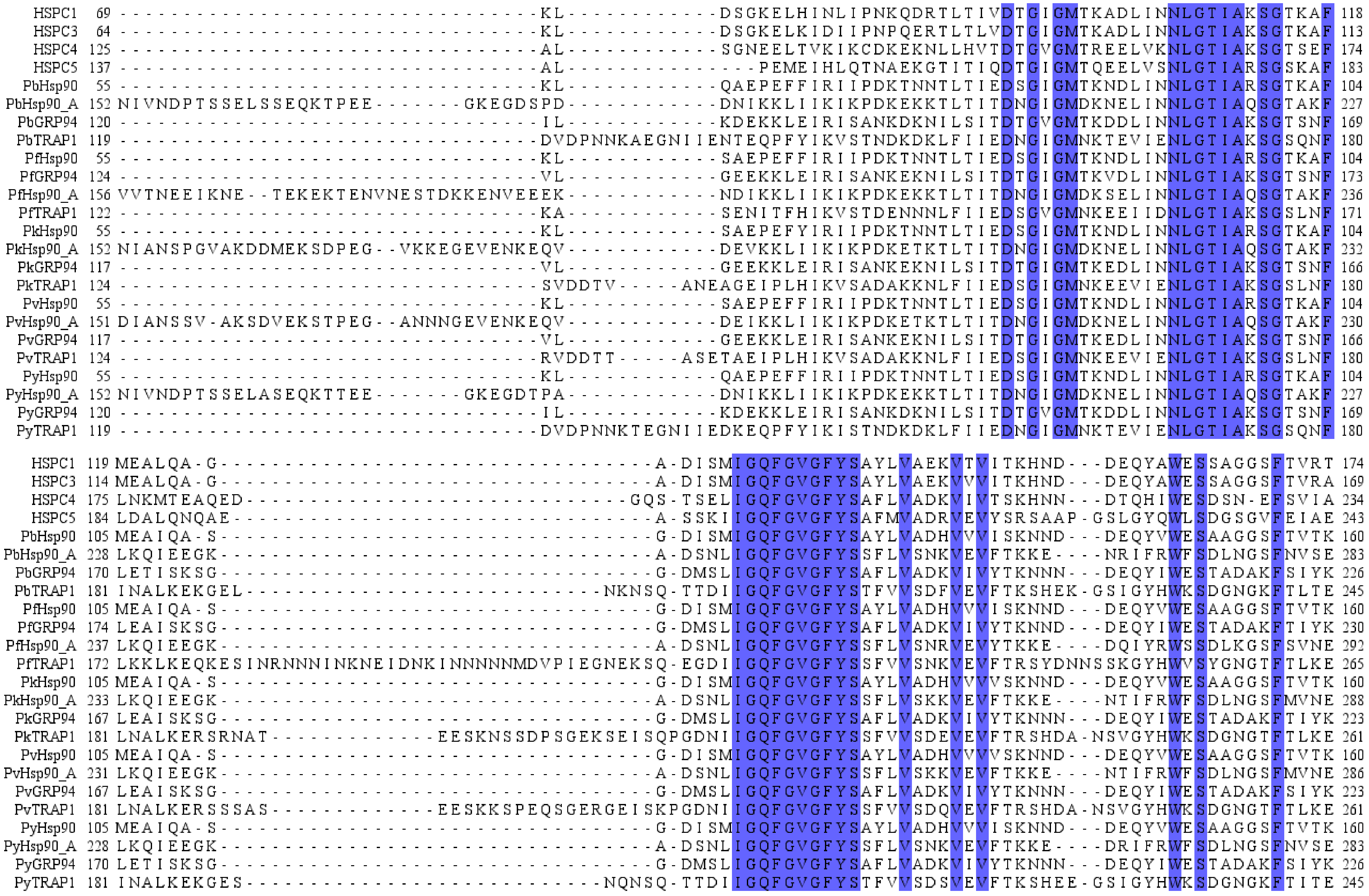
HSPC1 69 --------KL--------DSGKELHINLIPNKQDRTLTIVDTGIGMTKADLINNLGTIAKSGTKAF 118
HSPC3 64 --------KL--------DSGKELKIDIIPNPQERTLTLVDTGIGMTKADLINNLGTIAKSGTKAF 113
HSPC4 125 --------AL--------SGNEELTVKIKCDKEKNLLHVTDTGVGMTREELVKNLGTIAKSGTSEF 174
HSPC5 137 --------AL--------PEMEIHLQTNAEKGTITIQDTGIGMTQEELVSNLGTIARSGSKAF 183
PbHsp90 55 --------KL--------QAEPEFFIRIIPDKTNNTLTIEDSGIGMTKNDLINNLGTIARSGTKAF 104
PbHsp90_A 152 NIVNDPTSSELSSEQKTPEE--------GKEGDSPD--------DNIKKLIIKIKPDKEKKTLTITDNGIGMDKNELINNLGTIAQSGTAKF 227
PbGRP94 120 --------IL--------KDEKKLEIRISANKDKNILSITDTGVGMTKDDLINNLGTIAKSGTSNF 169
PbTRAP1 119 --------DVDPNNKAEGNIIENTEQPFYIKVSTNDKDKLFIIEDNGIGMNKTEVIENLGTIAKSGSQNF 180
PfHsp90 55 --------KL--------SAEPEFFIRIIPDKTNNTLTIEDSGIGMTKNDLINNLGTIARSGTKAF 104
PfGRP94 124 --------VL--------GEEKKLEIRISANKEKNILSITDTGIGMTKVDLINNLGTIAKSGTSNF 173
PfHsp90_A 156 VVTNEEIKNE--TEKEKTENVNESTDKKENVEEEK--------NDIKKLIIKIKPDKEKKTLTITDNGIGMDKSELINNLGTIAQSGTAKF 236
PfTRAP1 122 --------KA--------SENITFHIKVSTDENNNLFIIEDSGVGMNKEEIIDNLGTIAKSGSLNF 171
PkHsp90 55 --------KL--------SAEPEFYIRIIPDKTNNTLTIEDSGIGMTKNDLINNLGTIARSGTKAF 104
PkHsp90_A 152 NIANSPGVAKDDMEKSDPEG--VKKEGEVENKEQV--------DEVKKLIIKIKPDKETKTLTITDNGIGMDKNELINNLGTIAQSGTAKF 232
PkGRP94 117 --------VL--------GEEKKLEIRISANKEKNILSITDTGIGMTKEDLINNLGTIAKSGTSNF 166
PkTRAP1 124 --------SVDDTV----ANEAGEIPLHIKVSADAKKNLFIIEDSGIGMNKEEVIENLGTIAKSGSLNF 180
PvHsp90 55 --------KL--------SAEPEFFIRIIPDKTNNTLTIEDSGIGMTKNDLINNLGTIARSGTKAF 104
PvHsp90_A 151 DIANSSV-AKSDVEKSTPEG--ANNNGEVENKEQV--------DEIKKLIIKIKPDKETKTLTITDNGIGMDKNELINNLGTIAQSGTAKF 230
PvGRP94 117 --------VL--------GEEKKLEIRISANKEKNILSITDTGIGMTKEDLINNLGTIAKSGTSNF 166
PvTRAP1 124 --------RVDDTT----ASETAEIPLHIKVSADAKKNLFIIEDSGIGMNKEEVIENLGTIAKSGSLNF 180
PyHsp90 55 --------KL--------QAEPEFFIRIIPDKTNNTLTIEDSGIGMTKNDLINNLGTIARSGTKAF 104
PyHsp90_A 152 NIVNDPTSSELASEQKTTEE--------GKEGDTPA--------DNIKKLIIKIKPDKEKKTLTITDNGIGMDKNELINNLGTIAQSGTAKF 227
PyGRP94 120 --------IL--------KDEKKLEIRISANKDKNILSITDTGVGMTKDDLINNLGTIAKSGTSNF 169
PyTRAP1 119 --------DVDPNNKTEGNIIEDKEQPFYIKISTNDKDKLFIIEDNGIGMNKTEVIENLGTIAKSGSQNF 180
HSPC1 119 MEALQA-G--------A-DISMIGQFGVGFYSAYLVAEKVTVITKHND---DEQYAWESSAGGSFTVRT 174
HSPC3 114 MEALQA-G--------A-DISMIGQFGVGFYSAYLVAEKVVVITKHND---DEQYAWESSAGGSFTVRA 169
HSPC4 175 LNKMTEAQED--------GQS-TSELIGQFGVGFYSAFLVADKVIVTSKHNN--DTQHIWESDSN-EFSVIA 234
HSPC5 184 LDALQNQAE--------A-SSKIIGQFGVGFYSAFMVADRVEVYSRSAAP-GSLGYQWLSDGSGVFEIAE 243
PbHsp90 105 MEAIQA-S--------G-DISMIGQFGVGFYSAYLVADHVVVISKNND---DEQYVWESAAGGSFTVTK 160
PbHsp90_A 228 LKQIEEGK--------A-DSNLIGQFGVGFYSSFLVSNKVEVFTKKE----NRIFRWFSDLNGSFNVSE 283
PbGRP94 170 LETISKSG--------G-DMSLIGQFGVGFYSAFLVADKVIVYTKNNN---DEQYIWESTADAKFSIYK 226
PbTRAP1 181 INALKEKGEL--------NKNSQ-TTDIIGQFGVGFYSTFVVSDFVEVFTKSHEK-GSIGYHWKSDGNGKFTLTE 245
PfHsp90 105 MEAIQA-S--------G-DISMIGQFGVGFYSAYLVADHVVVISKNND---DEQYVWESAAGGSFTVTK 160
PfGRP94 174 LEAISKSG--------G-DMSLIGQFGVGFYSAFLVADKVIVYTKNND---DEQYIWESTADAKFTIYK 230
PfHsp90_A 237 LKQIEEGK--------A-DSNLIGQFGVGFYSSFLVSNRVEVYTKKE----DQIYRWSSDLKGSFSVNE 292
PfTRAP1 172 LKKLKEQKESINRNNNINKNEIDNKINNNNNMDVPIEGNEKSQ-EGDIIGQFGVGFYSSFVVSNKVEVFTRSYDNNSSKGYHWVSYGNGTFTLKE 265
PkHsp90 105 MEAIQA-S--------G-DISMIGQFGVGFYSAYLVADHVVVVSKNND---DEQYVWESAAGGSFTVTK 160
PkHsp90_A 233 LKQIEEGK--------A-DSNLIGQFGVGFYSSFLVSKKVEVFTKKE----NTIFRWFSDLNGSFMVNE 288
PkGRP94 167 LEAISKSG--------G-DMSLIGQFGVGFYSAFLVADKVIVYTKNNN---DEQYIWESTADAKFTIYK 223
PkTRAP1 181 LNALKERSRNAT--------EESKNSSDPSGEKSEISQPGDNIIGQFGVGFYSSFVVSDEVEVFTRSHDA-NSVGYHWKSDGNGTFTLKE 261
PvHsp90 105 MEAIQA-S--------G-DISMIGQFGVGFYSAYLVADHVVVVSKNND---DEQYVWESAAGGSFTVTK 160
PvHsp90_A 231 LKQIEEGK--------A-DSNLIGQFGVGFYSSFLVSKKVEVFTKKE----NTIFRWFSDLNGSFMVNE 286
PvGRP94 167 LEAISKSG--------G-DMSLIGQFGVGFYSAFLVADKVIVYTKNNN---DEQYIWESTADAKFSIYK 223
PvTRAP1 181 LNALKERSSSAS--------EESKKSPEQSGERGEISKPGDNIIGQFGVGFYSSFVVSDQVEVFTRSHDA-NSVGYHWKSDGNGTFTLKE 261
PyHsp90 105 MEAIQA-S--------G-DISMIGQFGVGFYSAYLVADHVVVISKNND---DEQYVWESAAGGSFTVTK 160
PyHsp90_A 228 LKQIEEGK--------A-DSNLIGQFGVGFYSSFLVSNKVEVFTKKE----DRIFRWFSDLNGSFNVSE 283
PyGRP94 170 LETISKSG--------G-DMSLIGQFGVGFYSAFLVADKVIVYTKNNN---DEQYIWESTADAKFSIYK 226
PyTRAP1 181 INALKEKGES--------NQNSQ-TTDIIGQFGVGFYSTFVVSDSVEVFTKSHEE-GSIGYHWKSDGNGKFTITE 245

```
HSPC1      175 D------TGEPMGRGTKVILHLKEDQTEYLEERRIKEIVKKHSQFIGYPITLFVEKERDKEVSD------------------------------- 232
HSPC3      170 D------HGEPIGRGTKVILHLKEDQTEYLEERRVKEVVKKHSQFIGYPITLYLEKEREKEISD------------------------------- 227
HSPC4      235 D-----PRGNTLGRGTTITLVLKEEASDYLELDTIKNLVKKYSQFINFPIYVWSSKTETVEEPM------------------------------- 293
HSPC5      244 A--------SGVRTGTKIIIHLKSDCKEFSSEARVRDVVTKYSNFVSFPLYLNGR---------------------------------------- 290
PbHsp90    161 D-----ETNEKIGRGTKIILHLKEDQLEYLEEKRIKDLVKKHSEFISFPIKLYCERQNEKEITE------------------------------- 219
PbHsp90_A  284 IKKYEQEYEDIKTSGTKIVLHLKEECDEYLEDYKLKELIKKYSEFIKFPIEIWSEKIDYERVPD------------------------------- 347
PbGRP94    227 D-----PRGATLKRGTRISLHLKEDATNLLNDKKLTDLIAKYSQFIQFPIYLLYENVYTEEVLA------------------------------- 285
PbTRAP1    246 D--------NSIERGTKIICHLKEACSEFSNINKVQTIVEKFSSFINFPVYILNKKQTPIEPKS------------------------------- 301
PfHsp90    161 D-----ETNEKLGRGTKIILHLKEDQLEYLEEKRIKDLVKKHSEFISFPIKLYCERQNEKEITA------------------------------- 219
PfGRP94    231 D-----PRGATLKRGTRISLHLKEDATNLLNDKKLMDLISKYSQFIQFPIYLLHENVYTEEVLA------------------------------- 289
PfHsp90_A  293 IKKYDQEYDDIKGSGTKIILHLKEECDEYLEDYKLKELIKKYSEFIKFPIEIWSEKIDYERVPD------------------------------- 356
PfTRAP1    266 V--------DNIPKGTKIICHLKDSCKEFSNIQNVQKIVEKFSSFINFPVYVLKKKKILQTRKDIEQNELNDHTQQNELNHHNDQNDHTQQNELN 352
PkHsp90    161 D-----ESNEKIGRGTKIILHLKDDQLEYLEEKRIKDLVKKHSEFISFPIKLYCERQNEKEITA------------------------------- 219
PkHsp90_A  289 IKKYEQEYEDIKSSGTKIVLHLKEECDEYLEDYKLKELIKKYSEFIKFPIEIWSEKIDYERVPD------------------------------- 352
PkGRP94    224 D-----PRGSTLKRGTRISLHLKEDATNLMNDKKLVDLISKYSQFIQYPIYLLHENVYTEEVLA------------------------------- 282
PkTRAP1    262 V--------EDLPRGTKIVCHLKESCKEFSNIHRVQEIVEKFSSFINFPVYIVNRKKDVKGSS-------------------------------- 316
PvHsp90    161 D-----ETNEKMGRGTKIILHLKDDQLEYLEEKRIKDLVKKHSEFISFPIKLYCERQNEKEITA------------------------------- 219
PvHsp90_A  287 IKKYEQEYEDIQSSGTKIVLHLKEECDEYLEDYKLKELIKKYSEFIKFPIEIWSEKIDYERVPD------------------------------- 350
PvGRP94    224 D-----PRGSTLKRGTRISLHLKDDATNLMNDKKLVDLISKYSQFIQYPIYLLHENVYTEEVLA------------------------------- 282
PvTRAP1    262 V--------EDLPRGTKIVCHLKDSCKEFANIHRVQEIVEKFSSFINFPVYIVNRKKDIKGSS-------------------------------- 316
PyHsp90    161 D-----ETNEKIGRGTKIILHLKEDQLEYLEEKRIKDLVKKHSEFISFPIKLYCERQNEKEITA------------------------------- 219
PyHsp90_A  284 IKKYEQEYEDIKTSGTKIVLHLKEECDEYLEDYKLKELIKKYSEFIKFPIEIWSEKIDYERVPD------------------------------- 347
PyGRP94    227 D-----PRGATLKRGTRISLHLKEDATNLLNDKKLTDLISKYSQFIQFPIYLLYENVYTEEVLA------------------------------- 285
PyTRAP1    246 D--------NSIKRGTKIVCHLKEACSEFSNINKIQTIVEKFSSFINFPVYILNKKQAPLESAS------------------------------- 301

HSPC1      233 -----------------------DE---AEEKE----DK---------------------EEEK------------------------------- 245
HSPC3      228 -----------------------DE---AEEEK----GE------------------------K------------------------------- 237
HSPC4      294 -----------------------EEEE-AA-KE---E---------------------------------------------------------- 302
HSPC5          -----------------------------------------------------------------------------------------------
PbHsp90    220 -----------------------SE---EEAQD----GE------------------------K-K----------------EGED--AE-KKED 240
PbHsp90_A  348 -----------------------DS---V------------------------------------------------------------------ 350
PbGRP94    286 -----------------------DI---AKEME-------------------------------------------------------------- 292
PbTRAP1    302 -----------------------VS---------------------------------------------------------------------- 303
PfHsp90    220 -----------------------SE---EEEGE----GEGEREGEEEEEKKKKTGEDKNADESK-E----------------ENED--EE-KKED 264
PfGRP94    290 -----------------------DI---AKDMV-------------------------------------------------------------- 296
PfHsp90_A  357 -----------------------DS---V------------------------------------------------------------------ 359
PfTRAP1    353 HHNDQNDHTQQNELNHHNDQNDHTQ-QNELNHH--------------------------------------------------------VEHHKN 390
PkHsp90    220 -----------------------SE---DEAEEEDADGE------------------------K-KKEG--------KDELEEGEDADKE-KKED 254
PkHsp90_A  353 -----------------------DS---V------------------------------------------------------------------ 355
PkGRP94    283 -----------------------DI---AKEME-------------------------------------------------------------- 289
PkTRAP1    317 -------------------RLVKGD-DMQNEES--------------------------------------------------------S-SLPQ 334
PvHsp90    220 -----------------------SE---DEAEEEDAEGE------------------------KKKKEGKDQLDDGDKQAQEGEGADNKE-KKEH 263
PvHsp90_A  351 -----------------------DS---V------------------------------------------------------------------ 353
PvGRP94    283 -----------------------DI---AKEME-------------------------------------------------------------- 289
PvTRAP1    317 -------------------RLVKGD-DAAHEEA--------------------------------------------------------SPPQAE 335
PyHsp90    220 -----------------------SE---DEEAQ---DGE------------------------K-K----------------EGED--AE-KKED 241
PyHsp90_A  348 -----------------------DS---V------------------------------------------------------------------ 350
PyGRP94    286 -----------------------DI---AKEME-------------------------------------------------------------- 292
PyTRAP1    302 -----------------------AD---------------------------------------------------------------------- 303
```

```
HSPC1      246 EKEE---K---ESEDKPEIE-------------------DVGSDEEEE-KKD-GDKKKKKKIKEKYIDQEELNKTKPIWTRNPDDITNEEYGEFY 313
HSPC3      238 EEED---K---DDEEKPKIE-------------------DVGSDEEDD-SGK-DKKKKTKKIKEKYIDQEELNKTKPIWTRNPDDITQEEYGEFY 305
HSPC4      303 --------------KEESDD-------------------EA--AVEEEEEE---KKPKTKKVEKTVWDWELMNDIKPIWQRPSKEVEEDEYKAFY 359
HSPC5      291 ----------------------------------------------------------------------RMNTLQAIWMMDPKDVREWQHEEFY 315
PbHsp90    241 DGEQ---K---DGEERPKVE-------------------DVTEELENA-EKK--KEKRKKKIHTVEHEWEELNKQKPLWMRKPEEVTNEEYASFY 307
PbHsp90_A  351 -------------------------------------------SLKDG-D-----KMKMKTITKRYHEWEKINVQLPIWKQDEKQLTENDYYSFY 396
PbGRP94    293 --------------NDPNYD-------------------SV--KVEES-DD---PNKKTRTVEKRVKQWKLMNEQKPIWLRPPKELSEDDYKNFY 348
PbTRAP1    304 ------------ES----------------------------DKVGES-DK---VGESDKVSEIEQVQEILINSQKPLWCKD--EVSEEEHKKFF 352
PfHsp90    265 NEED---D---NKTDHPKVE-------------------DVTEELENA-EKK-KKEKRKKKIHTVEHEWEELNKQKPLWMRKPEEVTNEEYASFY 332
PfGRP94    297 --------------NDPNYD-------------------SV--KVEET-DD---PNKKTRTVEKKVKKWTLMNEQRPIWLRSPKELKDEDYKQFF 352
PfHsp90_A  360 -------------------------------------------SLKDG-D-----KMKMKTITKRYHEWEKINVQLPIWKQDEKSLTENDYYSFY 405
PfTRAP1    391 VVEN---K---NDEYEKNMDNRTSDNIKPESLSKQNDYENMNQCNNEE-TV---LNRTKELNEEHIVEEILVNKQKPLWCKD--NVTEEEHRHFF 473
PkHsp90    255 NEEE---EDKEKGDDHPKVE-------------------DVTEELENA-EKKKKKEKRKKKIHTVEHEWEELNKQKPLWMRKPEEVTNEEYASFY 326
PkHsp90_A  356 -------------------------------------------SLKDG-D-----KMKMKTITKRYHEWEKINVQLPIWKQDEKMLTENDYYSFY 401
PkGRP94    290 --------------NDPNYD-------------------SV--KVEET-DD---PNKKTRTVEKKVKKWKLMNEQKPIWLRPPKELTDADYKKFF 345
PkTRAP1    335 EGLH---K---DVEKEKAMD-------------------G-TTEVEDS-TT---AADTSKGETESLTDEVQINNQKPLWCKE--NVTEEEHNKFF 397
PvHsp90    264 NEEDEDKD---KGEDHPKVE-------------------DVTEELENA-EKKKKKEKKKKKIHTVEHEWEELNKQKPLWMRKPEEVTNEEYASFY 335
PvHsp90_A  354 -------------------------------------------SLKDG-D-----KMKMKTITKRYHEWEKINVQLPIWKQDEKKLTENDYYSFY 399
PvGRP94    290 --------------NDPNYD-------------------SV--KVEES-DD---PNKKTRTVEKKVKKWKLMNEQKPIWLRPPKELTDEDYKKFF 345
PvTRAP1    336 EGLL---N---EAEREKAME-------------------GVADGVTDA-TA---SADAPKGDAESLTDEVQINNQKPLWCKD--HVTEEEHEKFF 399
PyHsp90    242 DGEQ---K---DGEERPKVE-------------------DVTEELENA-EKK--KEKKKKKIHTVEHEWEELNKQKPLWMRKPEEVTNEEYASFY 308
PyHsp90_A  351 -------------------------------------------SLKDG-D-----KMKMKTITKRYHEWEKINVQLPIWKQDEKQLTENDYYSFY 396
PyGRP94    293 --------------NDPNYD-------------------SV--KVEES-DD---PNKKTRTVEKRVKQWKLMNEQKPIWLRSPKELSEDDYKNFY 348
PyTRAP1    304 ------------NAASA--D-----------------NAVNGDNVDNA-AN---TANADNAGESEQVQEILINSQKPLWCKD--EVSEEEHKKFF 361

HSPC1      314 KSLT------NDWEDHLAVKHFSVEGQLEFRALLFVPRRAPFDLFEN--R--KKKNNIKLYVRRVFIMDNCEELIPEYLNFIRGVVDSEDLPLNI 398
HSPC3      306 KSLT------NDWEDHLAVKHFSVEGQLEFRALLFIPRRAPFDLFEN--K--KKKNNIKLYVRRVFIMDSCDELIPEYLNFIRGVVDSEDLPLNI 390
HSPC4      360 KSFS------KESDDPMAYIHFTAEGEVTFKSILFVPTSAPRGLFDEYGS--KKSDYIKLYVRRVFITDDFHDMMPKYLNFVKGVVDSDDLPLNV 446
HSPC5      316 RYV------AQAHDKPRYTLHYKTDAPLNIRSIFYVPDMKPSMFDVS--R--ELGSSVALYSRKVLIQTKATDILPKWLRFIRGVVDSEDIPLNL 400
PbHsp90    308 KSLT------NDWEDHLAVKHFSVEGQLEFKALLFIPKRAPFDMFEN--R--KKRNNIKLYVRRVFIMDDCEEIIPEWLNFVKGVVDSEDLPLNI 392
PbHsp90_A  397 KNTF------KAYDDPLAYVHFNVEGQISFNSILYIPGSLPWELSKN--MFDEESRGIRLYVKRVFINDKFSESIPRWLTFLRGIVDSENLPLNV 483
PbGRP94    349 SVLT------GYNDAPLYQIHFFAEGEIEFKCLIYIPARAPSINEQM--F--SKQNSIKLYVRRVLVADQFVDFMPKYMSYVKGIVDSDDLPLNV 433
PbTRAP1    353 NFLNKNKSYSDDNKSYLYKLMYKTDAPMSIKSVFYIPEEAPSRLFQQ--N---YDIDVSLYCKKVLVKKCADNIIPKWLHFVKGIIDCEDMPLNI 442
PfHsp90    333 KSLT------NDWEDHLAVKHFSVEGQLEFKALLFIPKRAPFDMFEN--R--KKRNNIKLYVRRVFIMDDCEEIIPEWLNFVKGVVDSEDLPLNI 417
PfGRP94    353 SVLS------GYNDQPLYHIHFFAEGEIEFKCLIYIPSKAPSMNDQL--Y--SKQNSLKLYVRRVLVADEFVEFLPRYMSFVKGVVDSDDLPLNV 437
PfHsp90_A  406 KNTF------KAYDDPLAYVHFNVEGQISFNSILYIPGSLPWELSKN--MFDEESRGIRLYVKRVFINDKFSESIPRWLTFLRGIVDSENLPLNV 492
PfTRAP1    474 HFLNKNKSYNEDNKSYLYNMLYKTDAPLSIKSVFYIPEEAPSRLFQQ--S---NDIEISLYCKKVLVKKNADNIIPKWLYFVKGVIDCEDMPLNI 563
PkHsp90    327 KSLT------NDWEDHLAVKHFSVEGQLEFKALLFIPKRAPFDMFEN--R--KKRNNIKLYVRRVFIMDDCEEIIPEWLNFVKGVVDSEDLPLNI 411
PkHsp90_A  402 KNTF------KAYDDPLAYVHFNVEGQISFNSILYIPGSLPWELSKN--MFDEESRGIRLYVKRVFINDKFSESIPRWLTFLRGIVDSENLPLNV 488
PkGRP94    346 SVLS------GFNDEPLYHIHFFAEGEIEFKCLIYIPSRAPSINDHL--F--TKQNSIKLYVRRVLVADEFVEFLPRYMSFVKGVVDSDDLPLNV 430
PkTRAP1    398 KFLTKNKNYG-DDKGYVYKVLYKTDAPMSIKSVFYIPEEAPSRLFQQ--S---NEIEVSLYCKKVLVKKNADNIIPKWLHFVKGVIDCEDMPLNI 486
PvHsp90    336 KSLT------NDWEDHLAVKHFSVEGQLEFKALLFIPKRAPFDMFEN--R--KKRNNIKLYVRRVFIMDDCEEIIPEWLNFVKGVVDSEDLPLNI 420
PvHsp90_A  400 KNTF------KAYDDPLAYVHFNVEGQISFNSILYIPGSLPWELSKN--MFDEESRGIRLYVKRVFINDKFSESIPRWLTFLRGIVDSENLPLNV 486
PvGRP94    346 SVLS------GFNDEPLYHIHFFAEGEIEFKCLIYIPSRAPSINDHL--F--TKQNSIKLYVRRVLVADEFVEFLPRYMSFIKGVVDSDDLPLNV 430
PvTRAP1    400 KFLTKKKSYD-DDKGYVYKVLYKTDAPMSIKSVFYIPEEAPSRLFQQ--S---NEIEVSLYCKKVLVKKSADNIIPKWLHFVKGVIDCEDMPLNI 488
PyHsp90    309 KSLT------NDWEDHLAVKHFSVEGQLEFKALLFIPKRAPFDMFEN--R--KKRNNIKLYVRRVFIMDDCEEIIPEWLNFVKGVVDSEDLPLNI 393
PyHsp90_A  397 KNTF------KAYDDPLAYVHFNVEGQISFNSILYIPGSLPWELSKN--MFDEESRGIRLYVKRVFINDKFSESIPRWLTFLRGIVDSENLPLNV 483
PyGRP94    349 SVLT------GYNDAPLYQIHFFAEGEIEFKCLIYIPARAPSINEQM--F--SKQNSIKLYVRRVLVADQFVDFMPKYMSYVKGIVDSDDLPLNV 433
PyTRAP1    362 NFLNKNKSYSDENKSYLYKLMYKTDAPMSIKSVFYIPEEAPSRLFQQ--N---YDIDVSLYCKKVLVKKCADNIIPKWLHFVKGIIDCEDMPLNI 451
```

```
HSPC1      399 SREMLQQSKILKVIRKNLVKKCLELFTELAED------------------------------KENY-KKFYEQFSKNIKLGIHEDSQN---RKKL 459
HSPC3      391 SREMLQQSKILKVIRKNIVKKCLELFSELAED------------------------------KENY-KKFYEAFSKNLKLGIHEDSTN---RRRL 451
HSPC4      447 SRETLQQHKLLKVIRKKLVRKTLDMIKKIADD--------------------------------KYNDTFWKEFGTNIKLGVIEDHSN---RTRL 506
HSPC5      401 SRELLQESALIRKLRDVLQQRLIKFFIDQSKKD-----------------------------AEKY-AKFFEDYGLFMREGIVTATEQ-EVKEDI 464
PbHsp90    393 SRESLQQNKILKVIKKNLIKKCLDMFAELAEN------------------------------KDNY-KKFYEQFSKNLKLGIHEDNAN---RAKI 453
PbHsp90_A  484 GREILQKSKMLSIINKRIVLKSINMMRGLKETG-----------------------------GEKW-NKFLNTFGKYLKIGVVEDKEN---QEEI 545
PbGRP94    434 SREQLQQNKILKAISKRIVRKILDTFRKLYLDGKKNKDSLREQLEKETDDDKKKEIQKKLNEPSVY-KSIYKEYRKFFKSGCYEDDIN---RSKI 524
PbTRAP1    443 SRESMQNSTLMSKLSRIIVTKILKTLEKEASID-----------------------------EDKY-LKFYKNFSYNLKEGILEDSTKNMYKNVM 507
PfHsp90    418 SRESLQQNKILKVIKKNLIKKCLDMFSELAEN------------------------------KENY-KKFYEQFSKNLKLGIHEDNAN---RTKI 478
PfGRP94    438 SREQLQQNKILKAVSKRIVRKILDTFHKLYKEGKKNKETLRSELENETDEEKKKEITKKLSEPSTY-KLIYKEYRKFLKSGCYEDDIN---RNKI 528
PfHsp90_A  493 GREILQKSKMLSIINKRIVLKSISMMKGLKETG-----------------------------GDKW-TKFLNTFGKYLKIGVVEDKEN---QEEI 554
PfTRAP1    564 SRENMQDSSLINKLSRVVVSKILKTLEREADIN-----------------------------EEKY-LKFYKNYNYNLKEGVLEDSNKNHYKNSL 628
PkHsp90    412 SRESLQQNKILKVIKKNLIKKCLDMFSELAEN------------------------------KDNY-KKFYEQFSKNLKLGIHEDNAN---RAKI 472
PkHsp90_A  489 GREILQKSKMLSIINKRIVLKSISMMKGLKETG-----------------------------GEKW-NKFLNTFGKYLKIGVVEDKEN---QEEI 550
PkGRP94    431 SREQLQQNKILKAVSKRIVRKILDTFRTLYLNGKKNKEELRAELAKETDEEKKKDIQKKINEPSTY-KLIYKEYRKYLKTGCYEDDIN---RNKI 521
PkTRAP1    487 SRENMQDSTLINKISRVVVTKILKTLEKESTND-----------------------------EDKY-RKFYTNYSYYLKEGVLEDSSNNFYKNTL 551
PvHsp90    421 SRESLQQNKILKVIKKNLIKKCLDMFSELAEN------------------------------KDNY-KKFYEQFSKNLKLGIHEDNAN---RAKI 481
PvHsp90_A  487 GREILQKSKMLSIINKRIVLKSISMMKGLKETG-----------------------------GEKW-NKFLNTFGKYLKIGVVEDKEN---QEEI 548
PvGRP94    431 SREQLQQNKILKAVSKRIVRKILDTFRTLYTSGKKNKEDLRAQLAKETDEEKKKEIQKKINEPSTY-KLIYKEYRKYLKTGCYEDDIN---RSKI 521
PvTRAP1    489 SRENMQDSTLISKISRVVVTKILKTLEKEATND-----------------------------EEKY-RKFYANYSYYLKEGVLEDSSNQFYKGML 553
PyHsp90    394 SRESLQQNKILKVIKKNLIKKCLDMFAELAEN------------------------------KDNY-KKFYEQFSKNLKLGIHEDNAN---RAKI 454
PyHsp90_A  484 GREILQKSKMLSIINKRIVLKSINMMRGLKETG-----------------------------GEKW-NKFLNTFGKYLKIGVVEDKEN---QEEI 545
PyGRP94    434 SREQLQQNKILKAISKRIVRKILDTFRKLYLDGKKNKDSLREQLEKETDEDKKKEIQKKLSEPSVY-KSIYKEYRKFFKSGCYEDDIN---RSKI 524
PyTRAP1    452 SRESMQDTALMNKLSRIIVTKIIKTLEKEASIN-----------------------------EDKY-LKFYKNFSYNLKEGILEDSTKNMYKNVM 516

HSPC1      460 SELLRYYTSAS-GD-EMVSLKDYCTRMKENQKHIYYITGETKDQVANSAFVERLRKHGLEVIYMIEPIDEYCVQQL--KEFEGKTLVSVTKEGL- 549
HSPC3      452 SELLRYHTSQS-GD-EMTSLSEYVSRMKETQKSIYYITGESKEQVANSAFVERVRKRGFEVVYMTEPIDEYCVQQL--KEFDGKSLVSVTKEGL- 541
HSPC4      507 AKLLRFQSSHH--PTDITSLDQYVERMKEKQDKIYFMAGSSRKEAESSPFVERLLKKGYEVIYLTEPVDEYCIQAL--PEFDGKRFQNVAKEGV- 596
HSPC5      465 AKLLRYESSALPSG-QLTSLSEYASRMRAGTRNIYYLCAPNRHLAEHSPYYEAMKKKDTEVLFCFEQFDELTLLHL--REFDKKKLISVETDIVV 556
PbHsp90    454 TELLRFQTSKS-GD-EMIGLKDYVDRMKDNQKDIYYITGESINAVSNSPFLEALTKRGYEVIYMVDPIDEYAVQQL--KDFDGKKLKCCTKEGL- 543
PbHsp90_A  546 ASLVEFYSINS-GD-KKIDLDTYIEKMKSDQKCIYYISGENKKTAQNSPSLEKLKALNYDVLFSLEPIDEFCLSSLSVNKYKGYDVLDVNKADL- 637
PbGRP94    525 VKLLLFKTMLH--P-KSISLDTYVENMKPDQKFIYYASGESYEYLSKIPQLQIFKKKNIDVLFLTESVDESCIQRV--EDYDGKKFKSIQKGEI- 613
PbTRAP1    508 MNLLRFYSVNL--K-KYISINEYIKNFKNTQKNIYYFSANDINIALSSPYMESFKNKQVDVLLLFEEIDEFVLMNL--QSYKDSKFISIDSSQKE 597
PfHsp90    479 TELLRFQTSKS-GD-EMIGLKEYVDRMKENQKDIYYITGESINAVSNSPFLEALTKKGFEVIYMVDPIDEYAVQQL--KDFDGKKLKCCTKEGL- 568
PfGRP94    529 AKLLLFKTMQY--P-KSISLDTYIEHMKPDQKFIYYASGDSYEYLAKIPQLQIFKKKNIDVLFLTESVDESCIQRV--QEYEGKKFKSIQKGEI- 617
PfHsp90_A  555 ASLVEFYSINS-GD-KKTDLDSYIENMKEDQKCIYYISGENKKTAQNSPSLEKLKALNYDVLFSLEPIDEFCLSSLTVNKYKGYEVLDVNKADL- 646
PfTRAP1    629 MNLLRFYSINQ--N-KFISLKQYVNNFRNNQKNIYYFSANDKNVALNSPYMEPFKKQNIDVLLLLEEIDEFVLMNL--QTYKDAKFVSIDTSQNE 718
PkHsp90    473 TELLRFQTSKS-GD-EMIGLKEYVDRMKENQKDIYYITGESINAVSNSPFLEALTKKGFEVIYMVDPIDEYAVQQL--KDFEGKKLKCCTKEGL- 562
PkHsp90_A  551 ASLVEFYSINS-GD-KKIDLDTYIENMKTDQKCIYYISGENKKTAQNSPSLEKLKALNYDVLFSLEPIDEFCLSSLTVNKYKGYDVLDVNKADL- 642
PkGRP94    522 VKLLLFKTMLH--P-KSISLDTYIENMKPDQKFIYYASGESYEYLSKIPQLQIFKKKNIDVVFLTESVDESCVQRV--QEYDGKKFKSIQKGEI- 610
PkTRAP1    552 MNLLRFYSIEQ--R-KNISLKDYVANFKNSQKKIYYFCVNELNVALSSPYMEPFKKQGVDVLLLFEEIDEFVLMNL--QEFNGAKFVSIDSSQNE 641
PvHsp90    482 TELLRFQTSKS-GD-EMIGLKEYVDRMKENQKDIYYITGESINAVSNSPFLEALTKKGFEVIYMVDPIDEYAVQQL--KDFEGKKLKCCTKEGL- 571
PvHsp90_A  549 ASLVEFYSINS-GD-KKIDLDTYIENMKPDQKCIYYISGENKKTAQNSPSLEKLKALNYDVLFSLEPIDEFCLSSLTVNKYKGYDVLDVNKADL- 640
PvGRP94    522 VKLLLFKTMLH--P-KSISLDTYVENMKPDQKFIYYASGDSYEYLSKIPQLQIFKKKNIDVVFLTESVDESCVQRV--QEYDGKKFKSIQKGEI- 610
PvTRAP1    554 MNLLRFYSIQQ--R-KNIPLKEYVANFKKAQKKIYYFCVNDLDVALSSPYMEPFKKQGVDVLLLFEEIDEFVLMNL--QEFNGSKFVSIDSSQNE 643
PyHsp90    455 TELLRFQTSKS-GD-EMIGLKDYVDRMKDNQKDIYYITGESINAVSNSPFLEALTKRGFEVIYMVDPIDEYAVQQL--KDFDGKKLKCCTKEGL- 544
PyHsp90_A  546 ASLVEFYSINS-GD-KKIDLDTYIEKMKSDQKCIYYISGENKKTAQNSPSLEKLKALNYDVLFSLEPIDEFCLSSLSVNKYKGYDVLDVNKADL- 637
PyGRP94    525 VKLLLFKTMLH--P-KSISLDTYVENMKPDQKFIYYASGESYEYLSKIPQLQIFKKKNIDVLFLTESVDESCIQRV--EDYDGKKFKSIQKGEI- 613
PyTRAP1    517 LNLLRFYSVNL--K-KYISLNEYVKNFKNTQKNIYYFSANDINIALSSPYMESFKNKQVDVLLLFEEIDEFVLMNL--QSYNDSKFVSIDSSQKE 606
```

```
HSPC1      550 ELPE-----------------------DE-EEKK--KQEEKKTKFENLCKIMKD-ILEKKVEKVVVSNRLVTSPCCIVTSTYGWTANMERIMKAQ 617
HSPC3      542 ELPE-----------------------DE-EEKK--KMEESKAKFENLCKLMKE-ILDKKVEKVTISNRLVSSPCCIVTSTYGWTANMERIMKAQ 609
HSPC4      597 KFDE-----------------------SE-KTKE--SREAVEKEFEPLLNWMKDKALKDKIEKAVVSQRLTESPCALVASQYGWSGNMERIMKAQ 665
HSPC5      557 DHYKE-------------------EKFED-RSPA--AECLSEKETEELMAWMRN-VLGSRVTNVKVTLRLDTHPAMVTVL---EMGAARHFLRMQ 625
PbHsp90    544 DIED-----------------------SE-EAKK--SFETLKAEYEGLCKVIKD-VLHEKVEKVVVGQRITDSPCVLVTSEFGWSANMERIMKAQ 611
PbHsp90_A  638 KLKT-----------------------EN-DQNKSDNINKLKIKYEILCRWLHN-KFSHKIHEVRISDRLIDSPSLLVQGEMGISPSMQKYMKQQ 707
PbGRP94    614 TFDL-----------------------TE-EEKK--REEKVKKMYTALIDVISD-TLRNKIFKVEISRRLVDAPCAVVSTEWGLSGQMEKLMKIN 681
PbTRAP1    598 DLDEIILNNENTDMN---TANTNNNNNSD-SNNN--KIPFTQDQKLELEKYIKQ-VLGPKCSDVKFSERLSLSPAVVTGF---LSPTLRRVMKAT 682
PfHsp90    569 DIDD-----------------------SE-EAKK--DFETLKAEYEGLCKVIKD-VLHEKVEKVVVGQRITDSPCVLVTSEFGWSANMERIMKAQ 636
PfGRP94    618 SFEL-----------------------TE-EEKK--KEQQMQKMYKALIDVISD-TLKNQIFKVEISRRLVDAPCAVVSTEWGLSGQMEKLMKMN 685
PfHsp90_A  647 KLKK-----------------------EN-DQNKSDSLDKQKMEYEILCRWLHN-KFSHKVHEVRISDRLINSPALLVQGEMGMSPSMQKYMKQQ 716
PfTRAP1    719 DFDEAVLNTNKN---------------DN-EKKQ--SIFFNDEQKKELQAYFKQ-VLGSKCSDVKFSERLTTSPAVVTGF---LSPTLRKVMKAT 791
PkHsp90    563 DIDD-----------------------SE-EAKK--TFETMKAEYEGLCKVIKD-VLHEKVEKVVVGQRITDSPCVLVTSEFGWSANMERIMKAQ 630
PkHsp90_A  643 KLKK-----------------------EN-DKNQTDSLDKLKMKYEILCRWLHN-KFSHKVHEVRISDRLINSPALLVQGEMGISPSMQKYMKQQ 712
PkGRP94    611 TFDL-----------------------TE-DEKK--KEEKVKKMYKALIDVISD-TLRNQIFKVEISRRLVDAPCAVVSTEWGLSGQMEKLMKIN 678
PkTRAP1    642 DFDQELLNSNRADGA----KAGDKDEASD-EARK--NGIFNDSQKSELAKYFKD-VLGSKCSDVKFSDRLTESPAVVTGF---LSPTLRKVMKAT 725
PvHsp90    572 DIDD-----------------------SE-EAKK--TFETMKAEYEGLCKVIKD-VLHEKVEKVVVGQRITDSPCVLVTSEFGWSANMERIMKAQ 639
PvHsp90_A  641 KLKK-----------------------EN-DQNQTDSLDKLKMKYEILCRWLHN-KFSHKVHEVRISDRLINSPALLVQGEMGISPSMQKYMKQQ 710
PvGRP94    611 TFEL-----------------------TD-DEKK--KEEKVKKMYKALIDVISD-TLRNQIFKVEISRRLVDAPCAVVSTEWGLSGQMEKLMKIN 678
PvTRAP1    644 DFDPELLNANRGDDG---AKGGDKEEASN-GGEK--NRIFNDSQKSELAKYFKD-VLGSKCSDVKFSDRLTESPAVVTGF---LSPTLRKVMKAT 728
PyHsp90    545 DIED-----------------------SE-EEKK--SFETLKAEYEGLCKVIKD-VLHEKVEKVVVGQRITDSPCVLVTSEFGWSANMERIMKAQ 612
PyHsp90_A  638 KLKT-----------------------EN-DQNKSDNINKLKIKYEILCRWLHN-KFSHKIHEVRISDRLIDSPSLLVQGEMGISPSMQKYMKQQ 707
PyGRP94    614 TFDL-----------------------TE-EEKK--REEKVKKMYTALIDVISD-TLRNKIFKVEISRRLVDAPCAVVSTEWGLSGQMEKLMKIN 681
PyTRAP1    607 DLDEIILSNENSDGKNSDGKNGDDQNSDGKNNMN--KIPFTQDQKIELEKYIKQ-VLGPKCSDVKFSERLSLSPAVVTGF---LSPTLRRVMKAT 695

HSPC1      618 A----LRDN----STMG----------YM-AAKKHLEINPDHSIIETLRQKAEADKNDKSVKDLVILLYETALLSSGFSLEDPQTHANRIYRMIK 693
HSPC3      610 A----LRDN----STMG----------YM-MAKKHLEINPDHPIVETLRQKAEADKNDKAVKDLVVLLFETALLSSGFSLEDPQTHSNRIYRMIK 685
HSPC4      666 A-----YQTGKDISTNY----------YA-SQKKTFEINPRHPLIRDMLRRIKEDEDDKTVLDLAVVLFETATLRSGYLLPDTKAYGDRIERMLR 744
HSPC5      626 Q----LAKT----QEER----------AQ-LLQPTLEINPRHALIKKLNQLRASEPG--LAQLLVDQIYENAMIAAGL-VDDPRAMVGRLNELLV 698
PbHsp90    612 A----LRDN----SMTS----------YM-LSKKIMEINARHPIITALKQKADADKSDKTVKDLIWLLFDTSLLTSGFALEEPTTFSKRIHRMIK 687
PbHsp90_A  708 ATAQGMSEN----EMFG----------GQSMNQPVLEINPNHYIIKQLNHLIQIDKMNPKNDEIAEQIFDVASMQGGYTIDDTGRFAKRVIGMME 788
PbGRP94    682 I-----NNN----DQVK----------AM-SGQKILEINPDHPIMIDLLKRSVENPKDPELVESIKVIYQSAKLASGFDLEDTSDLAQIVYDHIN 756
PbTRAP1    683 M-----KNS----DMNEN--------MLQ-NLPVTLEINPTHTIITSIYHLKNANEE--VAKLLIEQLYDNACIAAGI-LEDPRSLLTKLNELLL 756
PfHsp90    637 A----LRDN----SMTS----------YM-LSKKIMEINARHPIISALKQKADADKSDKTVKDLIWLLFDTSLLTSGFALEEPTTFSKRIHRMIK 712
PfGRP94    686 V-----SNS----DQIK----------AM-SGQKILEINPNHPIMIDLLKRSVTNPKDLELTNSIKIMYQSAKLASGFDLEDTADLAQIVYDHIN 760
PfHsp90_A  717 ATAQGISEN----EMFG----------GQSANQPVLEINPNHFIIKQLNHLIQIDKMNLQNSEIAEQIFDVASMQGGYTIDDTGLFAKRVIGMME 797
PfTRAP1    792 M-----KNS----DFNDNTNNSNNMNMFQ-NLPATLELNPSHTIVTSIYHLKNTNQE--VAKLLVQQLYDNACIAAGI-LEDPRSLLSKLNELLL 873
PkHsp90    631 A----LRDN----SMTS----------YM-LSKKIMEINARHPIITALKQKADADKSDKTVKDLIWLLFDTSLLTSGFALEEPTTFSKRIHRMIK 706
PkHsp90_A  713 ATAQGMSEN----EMFG----------GQSANQPVLEINPNHYIIKQLNHLIQIDKMNSQNSEIAEQIFDIASMQGGYTIDDTGLFAKRVIGMME 793
PkGRP94    679 V-----NNS----DQIR----------AM-SGQKILEINPDHPIMIDLLKRSVSNPKDSQLTESIKIIYQSAKLASGFDLEDTADLAQIVYDHIN 753
PkTRAP1    726 M-----KNS----SFQDNN-------MLN-NLPATLELNPSHTIVTSIFHLKNTNQD--VAKLLVQQLYDNACIAAGI-LEDPRSLLTKLNELLL 800
PvHsp90    640 A----LRDN----SMTS----------YM-LSKKIMEINARHPIITALKQKADADKSDKTVKDLIWLLFDTSLLTSGFALEEPTTFSKRIHRMIK 715
PvHsp90_A  711 ATAQGMSEN----EMFG----------GQSANQPVLEINPNHYIIKQLNHLIQIDKMNSQNSEIAEQIFDIASMQGGYTIDDTGLFAKRVIGMME 791
PvGRP94    679 V-----NNA----DQIK----------AM-SGQKILEINPDHPIMIDLLKRSVSNPKDSQLTESIKIIYQSAKLASGFDLEDTADLAQIVYDHIN 753
PvTRAP1    729 M-----KNA----SFQDNS-------MLH-NLPATLELNPSHTIVTSIFHLKNTNQE--VAKLLVQQLYDNACIAAGI-LEDPRSLLTKLNELLL 803
PyHsp90    613 A----LRDN----SMTS----------YM-LSKKIMEINARHPIITALKQKADADKSDKTVKDLIWLLFDTSLLTSGFALEEPTTFSKRIHRMIK 688
PyHsp90_A  708 ATAQGMSEN----EMFG----------GQSMNQPVLEINPNHYIIKQLNHLIQIDKMNPKNDEIAEQIFDVASMQGGYTIDDTGRFAKRVIGMME 788
PyGRP94    682 I-----NNT----DQVK----------AM-SGQKILEINPDHPIMIDLLKRSVENPKDPELVESIKVIYQSAKLASGFDLEDTSDLAQIVYDHIN 756
PyTRAP1    696 M-----KNS----DMNDN--------MLQ-NLPATLEINPTHTIIISIYHLKNTNEE--VAKLLVEQLYDNACIAAGI-LEDPRSLLTKLNELLL 769
```

```
HSPC1      694 LGLGI-DED-DPTADDTSAAVTEEMP-PL--EG------DDD----------------------------------------------------- 724
HSPC3      686 LGLGI-DED-EVAAEEPNAAVPDEIP-PL--EG------DED----------------------------------------------------- 716
HSPC4      745 LSLNI-DPD-AKVE--------EE-P-EEEPEE------TAE----------------------------------------------------- 768
HSPC5      699 KAL-------------------------------------------------------------------------------------------- 701
PbHsp90    688 LGLSI-DED-DNND--------IELP-PL--EE------TIEG---------------------------------------------------- 711
PbHsp90_A  789 KNAQAYLKD-VQDD--------IDIT-PS--NN------YEDT---------------------------------------------------- 813
PbGRP94    757 QTLGV-DNT-LKID--------DLDP-AI--FE------TKKI---------------------------------------------------- 780
PbTRAP1    757 LTARY-AYHYEKKN--------DACSESV--DKT------------------------------------------------------------- 779
PfHsp90    713 LGLSI-DEE-ENND--------IDLP-PL--EE------TVDA---------------------------------------------------- 736
PfGRP94    761 QKLGV-DNN-LKID--------DLDP-SI--FE------TKKI---------------------------------------------------- 784
PfHsp90_A  798 KNAEQYLMN-VQSN--------ISNN-TL--NN------N------------------------------------------------------- 819
PfTRAP1    874 LTARY-AYHYEKKD--------IQEN-TK--DQ------PIDV---------------------------------------------------- 898
PkHsp90    707 LGLSI-DED-ENND--------IELP-PL--EE------TIDA---------------------------------------------------- 730
PkHsp90_A  794 KNAQTYLMN-VQGN--------LDDK-PS--ES------SSSSSSEQASNSAQGNSTLDSTGEKSPPLDNPNSSSESLGLSEPNADASSEGGPGE 870
PkGRP94    754 QKLGV-DNN-LKID--------DLDP-AI--FE------TKKL---------------------------------------------------- 777
PkTRAP1    801 LTARY-AYHYEKNA--------DGVS-GV--QE-----DPSPS---------------------------------------------------- 826
PvHsp90    716 LGLSI-DEE-ENND--------IELP-PL--EE------TIDA---------------------------------------------------- 739
PvHsp90_A  792 KNAQTYLMN-VQGN--------LDST-PS--ES------SPSSSSEPAQNSAQGDSTQGNAGGSNPALD-------------NPSSSSESAGLS 854
PvGRP94    754 QKLGV-DNN-LKID--------DLDP-AI--FE------TKKM---------------------------------------------------- 777
PvTRAP1    804 LTARY-AYHYEKNT--------EGGP-AL--QE-----EQSPS---------------------------------------------------- 829
PyHsp90    689 LGLSI-DED-DNND--------IELP-PL--EE------TIEG---------------------------------------------------- 712
PyHsp90_A  789 RNAQAYLKD-VQDD--------IDIT-PS--NN------SEDT---------------------------------------------------- 813
PyGRP94    757 QKLGV-DNT-LKID--------DLDP-AI--FE------TKKI---------------------------------------------------- 780
PyTRAP1    770 LTARY-AYHYEKKT--------DAHSESV--DKADAYPESVDK---------------------------------------------------- 801

HSPC1          -----------------------------------------------------------------------------------------------
HSPC3          -----------------------------------------------------------------------------------------------
HSPC4          -----------------------------------------------------------------------------------------------
HSPC5          -----------------------------------------------------------------------------------------------
PbHsp90        -----------------------------------------------------------------------------------------------
PbHsp90_A  814 --------------------ALNNTTPNDKSQLNSEQGGINQNDSISNNTLQES---------------------------------NEN-NS 852
PbGRP94        -----------------------------------------------------------------------------------------------
PbTRAP1        -----------------------------------------------------------------------------------------------
PfHsp90        -----------------------------------------------------------------------------------------------
PfGRP94        -----------------------------------------------------------------------------------------------
PfHsp90_A  820 ---------------TSGSEMPQNNSPNELQSEMKSTNGIDDNSNISENKINESSSNQNNIGENSIAEENNIKNIAESDVNKINLGENDVSQNT 898
PfTRAP1        -----------------------------------------------------------------------------------------------
PkHsp90        -----------------------------------------------------------------------------------------------
PkHsp90_A  871 ERLTASNLDGLDGDNANANANANANASASTSASTSASTSASANASNISADALRDS---------------------------------TLNGNN 931
PkGRP94        -----------------------------------------------------------------------------------------------
PkTRAP1        -----------------------------------------------------------------------------------------------
PvHsp90        -----------------------------------------------------------------------------------------------
PvHsp90_A  855 EPSADASSTGGFGEERLSASNLDGLGGDNASANANANESSNASTNSIGANGLSDS---------------------------------ALNGSN 915
PvGRP94        -----------------------------------------------------------------------------------------------
PvTRAP1        -----------------------------------------------------------------------------------------------
PyHsp90        -----------------------------------------------------------------------------------------------
PyHsp90_A  814 --------------------TLDNTTPNDKSQLNSEQSDINQNDSISNNTLQES---------------------------------NEN-NS 852
PyGRP94        -----------------------------------------------------------------------------------------------
PyTRAP1        -----------------------------------------------------------------------------------------------
```

```
HSPC1      725 -------------------------------------------------------------------------SRME-------EVD 732
HSPC3      717 ----A-----------------------------------------------------------------SRME-------EVD 724
HSPC4      769 ----DTTEDTEQDE-------D----------------E-EMDVGTD-EEEE-----------TA--KESTAEK-------DEL 803
HSPC5      702 ---------------------------------------------------------------------------------ERH 704
PbHsp90    712 ---VD-----------------------------------------------------------------SKME-------EVD 720
PbHsp90_A  853 ENYIN----------------G----------------N-PQEAGSEVSNDK-----------QF----D-DSS-------TIE 880
PbGRP94    781 ---DD-------QN-------V----------------E-EINIDDAIKKGD-----------SN----SESKN-------DEL 808
PbTRAP1    780 --------------------------------------D-GNNINDSVNANV-----------EK----VEEHD-------DRF 802
PfHsp90    737 ---TD-----------------------------------------------------------------SKME-------EVD 745
PfGRP94    785 ---ED-------EN--DSSKFE----------------E-EINIDDEIQKKD-----------NNVDNESNDKS-------DEL 821
PfHsp90_A  899 MHKQD----------------S----------------G-LFNLDPSILNSN-----------ML----SGSDK-------TLL 927
PfTRAP1    899 ---K----------------------------------DNTMAIDNIVNTKE----NNTNDDSVM----KEAQS-------SNL 930
PkHsp90    731 ---TD-----------------------------------------------------------------SKME-------EVD 739
PkHsp90_A  932 MNSLD----------------S----------------N-LYNIDRSIFNDK-----------MF----SGSDK-------TIL 960
PkGRP94    778 ---EQ-------EDSGDGQKFH----------------E-EINIDDEIQKQD-----------SA--TESSSKN-------DEL 814
PkTRAP1    827 ---E----------------------------------PSAGNVDRTIPATEASPAQADDEENSA----KEAHA-------SKL 862
PvHsp90    740 ---TD-----------------------------------------------------------------SKME-------EVD 748
PvHsp90_A  916 MNGLD----------------P----------------N-LYNIDRNIFNDK-----------MF----SGSDK-------TVL 944
PvGRP94    778 ---EQ-------EDSPDGQKFH----------------E-EINIDDEIQKQD-----------AA--PEAAPKN-------DEL 814
PvTRAP1    830 ---E----------------------------------V------------SQVSANGAAEEGSA----KEAQA-------SKL 853
PyHsp90    713 ---AD-----------------------------------------------------------------SKME-------EVD 721
PyHsp90_A  853 ENYIS----------------G----------------N-PQEAGSEVSDDK-----------QF----D-DSN-------ANE 880
PyGRP94    781 ---DD-------QN-------V----------------E-EINIDDAIKKGD-----------SN----SESKN-------DEL 808
PyTRAP1    802 ---AD-------AH-------PESVDKADAHSESVNKAE-ANNINDSASANA-----------EN----VEEHDDSTLKEAHKI 852
```

CYTOSOLIC HSP90 SEQUENCE ALIGNMENT

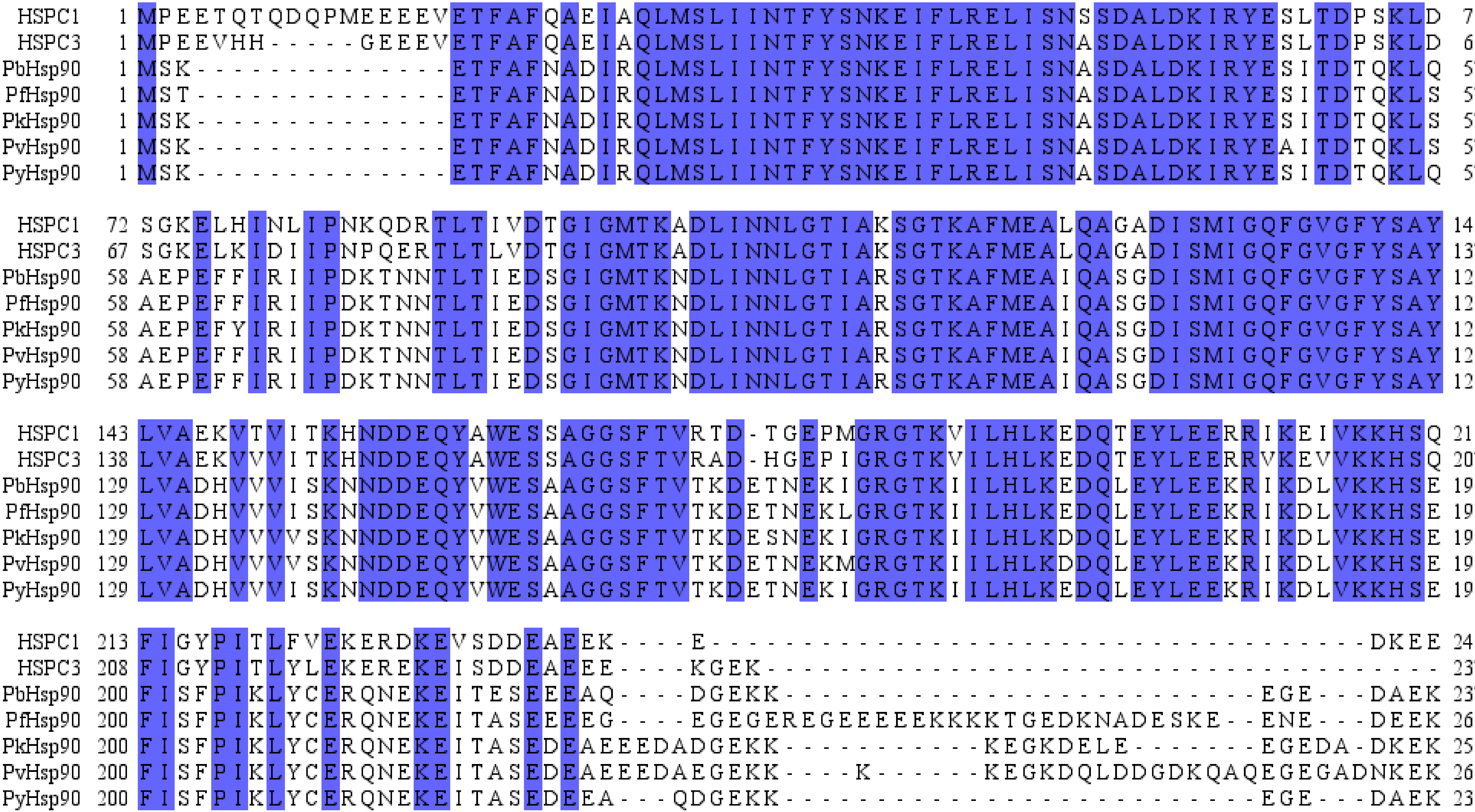
HSPC1 1 MPEETQTQDQPMEEEEVETFAFQAEIAQLMSLIINTFYSNKEIFLRELISNSSDALDKIRYESLTDPSKLD 7
HSPC3 1 MPEEVHH-----GEEEVETFAFQAEIAQLMSLIINTFYSNKEIFLRELISNASDALDKIRYESLTDPSKLD 6
PbHsp90 1 MSK--------------ETFAFNADIRQLMSLIINTFYSNKEIFLRELISNASDALDKIRYESITDTQKLQ 5
PfHsp90 1 MST--------------ETFAFNADIRQLMSLIINTFYSNKEIFLRELISNASDALDKIRYESITDTQKLS 5
PkHsp90 1 MSK--------------ETFAFNADIRQLMSLIINTFYSNKEIFLRELISNASDALDKIRYESITDTQKLS 5
PvHsp90 1 MSK--------------ETFAFNADIRQLMSLIINTFYSNKEIFLRELISNASDALDKIRYEAITDTQKLS 5
PyHsp90 1 MSK--------------ETFAFNADIRQLMSLIINTFYSNKEIFLRELISNASDALDKIRYESITDTQKLQ 5
HSPC1 72 SGKELHINLIPNKQDRTLTIVDTGIGMTKADLINNLGTIAKSGTKAFMEALQAGADISMIGQFGVGFYSAY 14
HSPC3 67 SGKELKIDIIPNPQERTLTLVDTGIGMTKADLINNLGTIAKSGTKAFMEALQAGADISMIGQFGVGFYSAY 13
PbHsp90 58 AEPEFFIRIIPDKTNNTLTIEDSGIGMTKNDLINNLGTIARSGTKAFMEAIQASGDISMIGQFGVGFYSAY 12
PfHsp90 58 AEPEFFIRIIPDKTNNTLTIEDSGIGMTKNDLINNLGTIARSGTKAFMEAIQASGDISMIGQFGVGFYSAY 12
PkHsp90 58 AEPEFYIRIIPDKTNNTLTIEDSGIGMTKNDLINNLGTIARSGTKAFMEAIQASGDISMIGQFGVGFYSAY 12
PvHsp90 58 AEPEFFIRIIPDKTNNTLTIEDSGIGMTKNDLINNLGTIARSGTKAFMEAIQASGDISMIGQFGVGFYSAY 12
PyHsp90 58 AEPEFFIRIIPDKTNNTLTIEDSGIGMTKNDLINNLGTIARSGTKAFMEAIQASGDISMIGQFGVGFYSAY 12
HSPC1 143 LVAEKVTVITKHNDDEQYAWESSAGGSFTVRTD-TGEPMGRGTKVILHLKEDQTEYLEERRIKEIVKKHSQ 21
HSPC3 138 LVAEKVVVITKHNDDEQYAWESSAGGSFTVRAD-HGEPIGRGTKVILHLKEDQTEYLEERRVKEVVKKHSQ 20
PbHsp90 129 LVADHVVVISKNNDDEQYVWESAAGGSFTVTKDETNEKIGRGTKIILHLKEDQLEYLEEKRIKDLVKKHSE 19
PfHsp90 129 LVADHVVVISKNNDDEQYVWESAAGGSFTVTKDETNEKLGRGTKIILHLKEDQLEYLEEKRIKDLVKKHSE 19
PkHsp90 129 LVADHVVVVSKNNDDEQYVWESAAGGSFTVTKDESNEKIGRGTKIILHLKDDQLEYLEEKRIKDLVKKHSE 19
PvHsp90 129 LVADHVVVVSKNNDDEQYVWESAAGGSFTVTKDETNEKMGRGTKIILHLKDDQLEYLEEKRIKDLVKKHSE 19
PyHsp90 129 LVADHVVVISKNNDDEQYVWESAAGGSFTVTKDETNEKIGRGTKIILHLKEDQLEYLEEKRIKDLVKKHSE 19
HSPC1 213 FIGYPITLFVEKERDKEVSDDEAEEK----E------------------------------------DKEE 24
HSPC3 208 FIGYPITLYLEKEREKEISDDEAEEE----KGEK------------------------------------- 23
PbHsp90 200 FISFPIKLYCERQNEKEITESEEEAQ----DGEKK--------------------------EGE---DAEK 23
PfHsp90 200 FISFPIKLYCERQNEKEITASEEEEG----EGEGEREGEEEEEKKKKTGEDKNADESKE--ENE---DEEK 26
PkHsp90 200 FISFPIKLYCERQNEKEITASEDEAEEEDADGEKK-----------KEGKDELE-------EGEDA-DKEK 25
PvHsp90 200 FISFPIKLYCERQNEKEITASEDEAEEEDAEGEKK----K------KEGKDQLDDGDKQAQEGEGADNKEK 26
PyHsp90 200 FISFPIKLYCERQNEKEITASEDEEA---QDGEKK--------------------------EGE---DAEK 23

```
HSPC1   244 -EKEKEE---KESEDKPEIEDVGSDEEEEKK-DGDKKKKKKIKEKYIDQEELNKTKPIWTRNPDDITNEEY 309
HSPC3   238 ---EEED---KDDEEKPKIEDVGSDEEDDSG-KDKKKKTKKIKEKYIDQEELNKTKPIWTRNPDDITQEEY 301
PbHsp90 238 KEDDGEQ---KDGEERPKVEDVTEELENAEK--KKEKRKKKIHTVEHEWEELNKQKPLWMRKPEEVTNEEY 303
PfHsp90 262 KEDNEED---DNKTDHPKVEDVTEELENAEK-KKKEKRKKKIHTVEHEWEELNKQKPLWMRKPEEVTNEEY 328
PkHsp90 252 KEDNEEEEDKEKGDDHPKVEDVTEELENAEKKKKKEKRKKKIHTVEHEWEELNKQKPLWMRKPEEVTNEEY 322
PvHsp90 261 KEHNEEDEDKDKGEDHPKVEDVTEELENAEKKKKKEKKKKKIHTVEHEWEELNKQKPLWMRKPEEVTNEEY 331
PyHsp90 239 KEDDGEQ---KDGEERPKVEDVTEELENAEK--KKEKKKKKIHTVEHEWEELNKQKPLWMRKPEEVTNEEY 304

HSPC1   310 GEFYKSLTNDWEDHLAVKHFSVEGQLEFRALLFVPRRAPFDLFENRKKKNNIKLYVRRVFIMDNCEELIPE 380
HSPC3   302 GEFYKSLTNDWEDHLAVKHFSVEGQLEFRALLFIPRRAPFDLFENKKKKNNIKLYVRRVFIMDSCDELIPE 372
PbHsp90 304 ASFYKSLTNDWEDHLAVKHFSVEGQLEFKALLFIPKRAPFDMFENRKKRNNIKLYVRRVFIMDDCEEIIPE 374
PfHsp90 329 ASFYKSLTNDWEDHLAVKHFSVEGQLEFKALLFIPKRAPFDMFENRKKRNNIKLYVRRVFIMDDCEEIIPE 399
PkHsp90 323 ASFYKSLTNDWEDHLAVKHFSVEGQLEFKALLFIPKRAPFDMFENRKKRNNIKLYVRRVFIMDDCEEIIPE 393
PvHsp90 332 ASFYKSLTNDWEDHLAVKHFSVEGQLEFKALLFIPKRAPFDMFENRKKRNNIKLYVRRVFIMDDCEEIIPE 402
PyHsp90 305 ASFYKSLTNDWEDHLAVKHFSVEGQLEFKALLFIPKRAPFDMFENRKKRNNIKLYVRRVFIMDDCEEIIPE 375

HSPC1   381 YLNFIRGVVDSEDLPLNISREMLQQSKILKVIRKNLVKKCLELFTELAEDKENYKKFYEQFSKNIKLGIHE 451
HSPC3   373 YLNFIRGVVDSEDLPLNISREMLQQSKILKVIRKNIVKKCLELFSELAEDKENYKKFYEAFSKNLKLGIHE 443
PbHsp90 375 WLNFVKGVVDSEDLPLNISRESLQQNKILKVIKKNLIKKCLDMFAELAENKDNYKKFYEQFSKNLKLGIHE 445
PfHsp90 400 WLNFVKGVVDSEDLPLNISRESLQQNKILKVIKKNLIKKCLDMFSELAENKENYKKFYEQFSKNLKLGIHE 470
PkHsp90 394 WLNFVKGVVDSEDLPLNISRESLQQNKILKVIKKNLIKKCLDMFSELAENKDNYKKFYEQFSKNLKLGIHE 464
PvHsp90 403 WLNFVKGVVDSEDLPLNISRESLQQNKILKVIKKNLIKKCLDMFSELAENKDNYKKFYEQFSKNLKLGIHE 473
PyHsp90 376 WLNFVKGVVDSEDLPLNISRESLQQNKILKVIKKNLIKKCLDMFAELAENKDNYKKFYEQFSKNLKLGIHE 446

HSPC1   452 DSQNRKKLSELLRYYTSASGDEMVSLKDYCTRMKENQKHIYYITGETKDQVANSAFVERLRKHGLEVIYMI 522
HSPC3   444 DSTNRRRLSELLRYHTSQSGDEMTSLSEYVSRMKETQKSIYYITGESKEQVANSAFVERVRKRGFEVVYMT 514
PbHsp90 446 DNANRAKITELLRFQTSKSGDEMIGLKDYVDRMKDNQKDIYYITGESINAVSNSPFLEALTKRGYEVIYMV 516
PfHsp90 471 DNANRTKITELLRFQTSKSGDEMIGLKEYVDRMKENQKDIYYITGESINAVSNSPFLEALTKKGFEVIYMV 541
PkHsp90 465 DNANRAKITELLRFQTSKSGDEMIGLKEYVDRMKENQKDIYYITGESINAVSNSPFLEALTKKGFEVIYMV 535
PvHsp90 474 DNANRAKITELLRFQTSKSGDEMIGLKEYVDRMKENQKDIYYITGESINAVSNSPFLEALTKKGFEVIYMV 544
PyHsp90 447 DNANRAKITELLRFQTSKSGDEMIGLKDYVDRMKDNQKDIYYITGESINAVSNSPFLEALTKRGFEVIYMV 517
```

```
HSPC1   523 EPIDEYCVQQLKEFEGKTLVSVTKEGLELPEDEEEKKKQEEKKTKFENLCKIMKDILEKKVEKVVVSNRLV 593
HSPC3   515 EPIDEYCVQQLKEFDGKSLVSVTKEGLELPEDEEEKKKMEESKAKFENLCKLMKEILDKKVEKVTISNRLV 585
PbHsp90 517 DPIDEYAVQQLKDFDGKKLKCCTKEGLDIEDSEEAKKSFETLKAEYEGLCKVIKDVLHEKVEKVVVGQRIT 587
PfHsp90 542 DPIDEYAVQQLKDFDGKKLKCCTKEGLDIDDSEEAKKDFETLKAEYEGLCKVIKDVLHEKVEKVVVGQRIT 612
PkHsp90 536 DPIDEYAVQQLKDFEGKKLKCCTKEGLDIDDSEEAKKTFETMKAEYEGLCKVIKDVLHEKVEKVVVGQRIT 606
PvHsp90 545 DPIDEYAVQQLKDFEGKKLKCCTKEGLDIDDSEEAKKTFETMKAEYEGLCKVIKDVLHEKVEKVVVGQRIT 615
PyHsp90 518 DPIDEYAVQQLKDFDGKKLKCCTKEGLDIEDSEEEKKSFETLKAEYEGLCKVIKDVLHEKVEKVVVGQRIT 588

HSPC1   594 TSPCCIVTSTYGWTANMERIMKAQALRDNSTMGYMAAKKHLEINPDHSIIETLRQKAEADKNDKSVKDLVI 664
HSPC3   586 SSPCCIVTSTYGWTANMERIMKAQALRDNSTMGYMMAKKHLEINPDHPIVETLRQKAEADKNDKAVKDLVV 656
PbHsp90 588 DSPCVLVTSEFGWSANMERIMKAQALRDNSMTSYMLSKKIMEINARHPIITALKQKADADKSDKTVKDLIW 658
PfHsp90 613 DSPCVLVTSEFGWSANMERIMKAQALRDNSMTSYMLSKKIMEINARHPIISALKQKADADKSDKTVKDLIW 683
PkHsp90 607 DSPCVLVTSEFGWSANMERIMKAQALRDNSMTSYMLSKKIMEINARHPIITALKQKADADKSDKTVKDLIW 677
PvHsp90 616 DSPCVLVTSEFGWSANMERIMKAQALRDNSMTSYMLSKKIMEINARHPIITALKQKADADKSDKTVKDLIW 686
PyHsp90 589 DSPCVLVTSEFGWSANMERIMKAQALRDNSMTSYMLSKKIMEINARHPIITALKQKADADKSDKTVKDLIW 659

HSPC1   665 LLYETALLSSGFSLEDPQTHANRIYRMIKLGLGIDEDDPTADDTSAAVTEEMPPLEGDDD--TSRMEEVD 732
HSPC3   657 LLFETALLSSGFSLEDPQTHSNRIYRMIKLGLGIDEDEVAAEEPNAAVPDEIPPLEGDED--ASRMEEVD 724
PbHsp90 659 LLFDTSLLTSGFALEEPTTFSKRIHRMIKLGLSIDEDDNN--------DIELPPLEETIEGVDSKMEEVD 720
PfHsp90 684 LLFDTSLLTSGFALEEPTTFSKRIHRMIKLGLSIDEEENN--------DIDLPPLEETVDATDSKMEEVD 745
PkHsp90 678 LLFDTSLLTSGFALEEPTTFSKRIHRMIKLGLSIDEDENN--------DIELPPLEETIDATDSKMEEVD 739
PvHsp90 687 LLFDTSLLTSGFALEEPTTFSKRIHRMIKLGLSIDEEENN--------DIELPPLEETIDATDSKMEEVD 748
PyHsp90 660 LLFDTSLLTSGFALEEPTTFSKRIHRMIKLGLSIDEDDNN--------DIELPPLEETIEGADSKMEEVD 721
```

APICOPLAST PLASMODIAL HSP90 SEQUENCE ALIGNMENT

```
PbHsp90_A    1 MQNAYISHKTKLILLF-FVVVFLKCNDIIIEAFNFSRSVEKLNYVLNYKNSNIYRIYHNINKSFLKK  66
PfHsp90_A    1 MQNVYVGNKIKFIILYFFCVLFLKDYER-SEAFNLARTTEKLNYILNYKTPNRYDLNNNVNKLFFEK  66
PkHsp90_A    1 MQNARVANKIKLMLCLLFVVALLKPNDV-TEAYNTARNAEKLNYILNYKNASRYNIDNRINKTFFKK  66
PvHsp90_A    1 MQNARVANKIKLILCL-LFAALLKPNDV-TEAYNTARNAEKLNYILNYKNASRYHIDNRINKTFLKK  65
PyHsp90_A    1 MQNAYISHKTKLMLLF-FIVVFLKCNDIIIEAFNFSRSVEKLNYVLNYKNSNIYNIDQNINKSFLKK  66

PbHsp90_A   67 RQ----FKRNSTVSFNNNVKTIDGDVQSDDTPVEKYNFKAEVNKVMDIIVNSLYTDKDVFLRELISN 129
PfHsp90_A   67 QKKKIEFSRKPLNSFNEDVKTIREDISSDSSPVEKYNFKAEVNKVMDIIVNSLYTDKDVFLRELISN 133
PkHsp90_A   67 QK----LKGNSLNSFNDDVKTIREDMSSESSPVEKYNFKAEVNKVMDIIVNSLYTDKDVFLRELISN 129
PvHsp90_A   66 KK----LKGNTLNSFNDDVKTIREDMSADSSPVEKYNFKAEVNKVMDIIVNSLYTDKDVFLRELISN 128
PyHsp90_A   67 RQ----FKRNSTVGFNNDVKISDGDVQSDDTPVEKYNFKAEVNKVMDIIVNSLYTDKDVFLRELISN 129

PbHsp90_A  130 ASDACDKKRIVLENEKRAKEAQNIVNDPTSSELSSEQKTPEEG-K-----EG-DSPDDNIKKLIIKI 189
PfHsp90_A  134 ASDACDKKRIILENNKLIKDAEVVTNEEIKNE--TEKEKTENVNESTDKKENVEEEKNDIKKLIIKI 198
PkHsp90_A  130 ASDACDKKRIILQNEKQMKEAQNIANSPGVAKDDMEKSDPEGVKKEGE-VEN-KEQVDEVKKLIIKI 194
PvHsp90_A  129 ASDACDKKRIILQNEKQMKEAQDIANSSV-AKSDVEKSTPEGANNNGE-VEN-KEQVDEIKKLIIKI 192
PyHsp90_A  130 ASDACDKKRIILENEKRAMEAQNIVNDPTSSELASEQKTTEEG-K-----EG-DTPADNIKKLIIKI 189

PbHsp90_A  190 KPDKEKKTLTITDNGIGMDKNELINNLGTIAQSGTAKFLKQIEEGKADSNLIGQFGVGFYSSFLVSN 256
PfHsp90_A  199 KPDKEKKTLTITDNGIGMDKSELINNLGTIAQSGTAKFLKQIEEGKADSNLIGQFGVGFYSSFLVSN 265
PkHsp90_A  195 KPDKETKTLTITDNGIGMDKNELINNLGTIAQSGTAKFLKQIEEGKADSNLIGQFGVGFYSSFLVSK 261
PvHsp90_A  193 KPDKETKTLTITDNGIGMDKNELINNLGTIAQSGTAKFLKQIEEGKADSNLIGQFGVGFYSSFLVSK 259
PyHsp90_A  190 KPDKEKKTLTITDNGIGMDKNELINNLGTIAQSGTAKFLKQIEEGKADSNLIGQFGVGFYSSFLVSN 256

PbHsp90_A  257 KVEVFTKKENRIFRWFSDLNGSFNVSEIKKYEQEYEDIKTSGTKIVLHLKEECDEYLEDYKLKELIK 323
PfHsp90_A  266 RVEVYTKKEDQIYRWSSDLKGSFSVNEIKKYDQEYDDIKGSGTKIILHLKEECDEYLEDYKLKELIK 332
PkHsp90_A  262 KVEVFTKKENTIFRWFSDLNGSFMVNEIKKYEQEYEDIKSSGTKIVLHLKEECDEYLEDYKLKELIK 328
PvHsp90_A  260 KVEVFTKKENTIFRWFSDLNGSFMVNEIKKYEQEYEDIQSSGTKIVLHLKEECDEYLEDYKLKELIK 326
PyHsp90_A  257 KVEVFTKKEDRIFRWFSDLNGSFNVSEIKKYEQEYEDIKTSGTKIVLHLKEECDEYLEDYKLKELIK 323
```

```
PbHsp90_A  324  KYSEFIKFPIEIWSEKIDYERVPDDSVSLKDGDKMKMKTITKRYHEWEKINVQLPIWKQDEKQLTEN  390
PfHsp90_A  333  KYSEFIKFPIEIWSEKIDYERVPDDSVSLKDGDKMKMKTITKRYHEWEKINVQLPIWKQDEKSLTEN  399
PkHsp90_A  329  KYSEFIKFPIEIWSEKIDYERVPDDSVSLKDGDKMKMKTITKRYHEWEKINVQLPIWKQDEKMLTEN  395
PvHsp90_A  327  KYSEFIKFPIEIWSEKIDYERVPDDSVSLKDGDKMKMKTITKRYHEWEKINVQLPIWKQDEKKLTEN  393
PyHsp90_A  324  KYSEFIKFPIEIWSEKIDYERVPDDSVSLKDGDKMKMKTITKRYHEWEKINVQLPIWKQDEKQLTEN  390

PbHsp90_A  391  DYYSFYKNTFKAYDDPLAYVHFNVEGQISFNSILYIPGSLPWELSKNMFDEESRGIRLYVKRVFIND  457
PfHsp90_A  400  DYYSFYKNTFKAYDDPLAYVHFNVEGQISFNSILYIPGSLPWELSKNMFDEESRGIRLYVKRVFIND  466
PkHsp90_A  396  DYYSFYKNTFKAYDDPLAYVHFNVEGQISFNSILYIPGSLPWELSKNMFDEESRGIRLYVKRVFIND  462
PvHsp90_A  394  DYYSFYKNTFKAYDDPLAYVHFNVEGQISFNSILYIPGSLPWELSKNMFDEESRGIRLYVKRVFIND  460
PyHsp90_A  391  DYYSFYKNTFKAYDDPLAYVHFNVEGQISFNSILYIPGSLPWELSKNMFDEESRGIRLYVKRVFIND  457

PbHsp90_A  458  KFSESIPRWLTFLRGIVDSENLPLNVGREILQKSKMLSIINKRIVLKSINMMRGLKETGGEKWNKFL  524
PfHsp90_A  467  KFSESIPRWLTFLRGIVDSENLPLNVGREILQKSKMLSIINKRIVLKSISMMKGLKETGGDKWTKFL  533
PkHsp90_A  463  KFSESIPRWLTFLRGIVDSENLPLNVGREILQKSKMLSIINKRIVLKSISMMKGLKETGGEKWNKFL  529
PvHsp90_A  461  KFSESIPRWLTFLRGIVDSENLPLNVGREILQKSKMLSIINKRIVLKSISMMKGLKETGGEKWNKFL  527
PyHsp90_A  458  KFSESIPRWLTFLRGIVDSENLPLNVGREILQKSKMLSIINKRIVLKSINMMRGLKETGGEKWNKFL  524

PbHsp90_A  525  NTFGKYLKIGVVEDKENQEEIASLVEFYSINSGDKKIDLDTYIEKMKSDQKCIYYISGENKKTAQNS  591
PfHsp90_A  534  NTFGKYLKIGVVEDKENQEEIASLVEFYSINSGDKKTDLDSYIENMKEDQKCIYYISGENKKTAQNS  600
PkHsp90_A  530  NTFGKYLKIGVVEDKENQEEIASLVEFYSINSGDKKIDLDTYIENMKTDQKCIYYISGENKKTAQNS  596
PvHsp90_A  528  NTFGKYLKIGVVEDKENQEEIASLVEFYSINSGDKKIDLDTYIENMKPDQKCIYYISGENKKTAQNS  594
PyHsp90_A  525  NTFGKYLKIGVVEDKENQEEIASLVEFYSINSGDKKIDLDTYIEKMKSDQKCIYYISGENKKTAQNS  591

PbHsp90_A  592  PSLEKLKALNYDVLFSLEPIDEFCLSSLSVNKYKGYDVLDVNKADLKLKTENDQNKSDNINKLKIKY  658
PfHsp90_A  601  PSLEKLKALNYDVLFSLEPIDEFCLSSLTVNKYKGYEVLDVNKADLKLKKENDQNKSDSLDKQKMEY  667
PkHsp90_A  597  PSLEKLKALNYDVLFSLEPIDEFCLSSLTVNKYKGYDVLDVNKADLKLKKENDKNQTDSLDKLKMKY  663
PvHsp90_A  595  PSLEKLKALNYDVLFSLEPIDEFCLSSLTVNKYKGYDVLDVNKADLKLKKENDQNQTDSLDKLKMKY  661
PyHsp90_A  592  PSLEKLKALNYDVLFSLEPIDEFCLSSLSVNKYKGYDVLDVNKADLKLKTENDQNKSDNINKLKIKY  658
```

```
PbHsp90_A 659 EILCRWLHNKFSHKIHEVRISDRLIDSPSLLVQGEMGISPSMQKYMKQQATAQGMSENEMFGGQSMN 725
PfHsp90_A 668 EILCRWLHNKFSHKVHEVRISDRLINSPALLVQGEMGMSPSMQKYMKQQATAQGISENEMFGGQSAN 734
PkHsp90_A 664 EILCRWLHNKFSHKVHEVRISDRLINSPALLVQGEMGISPSMQKYMKQQATAQGMSENEMFGGQSAN 730
PvHsp90_A 662 EILCRWLHNKFSHKVHEVRISDRLINSPALLVQGEMGISPSMQKYMKQQATAQGMSENEMFGGQSAN 728
PyHsp90_A 659 EILCRWLHNKFSHKIHEVRISDRLIDSPSLLVQGEMGISPSMQKYMKQQATAQGMSENEMFGGQSMN 725

PbHsp90_A 726 QPVLEINPNHYIIKQLNHLIQIDKMNPKNDEIAEQIFDVASMQGGYTIDDTGRFAKRVIGMMEKNAQ 792
PfHsp90_A 735 QPVLEINPNHFIIKQLNHLIQIDKMNLQNSEIAEQIFDVASMQGGYTIDDTGLFAKRVIGMMEKNAE 801
PkHsp90_A 731 QPVLEINPNHYIIKQLNHLIQIDKMNSQNSEIAEQIFDIASMQGGYTIDDTGLFAKRVIGMMEKNAQ 797
PvHsp90_A 729 QPVLEINPNHYIIKQLNHLIQIDKMNSQNSEIAEQIFDIASMQGGYTIDDTGLFAKRVIGMMEKNAQ 795
PyHsp90_A 726 QPVLEINPNHYIIKQLNHLIQIDKMNPKNDEIAEQIFDVASMQGGYTIDDTGRFAKRVIGMMERNAQ 792

PbHsp90_A 793 AYLKDVQDDIDITPSNNYEDTAL---NNTTPND--------------KSQLNSEQGGIN-------- 834
PfHsp90_A 802 QYLMNVQSNISNNTLNNNTSGSEMP-QNNSPNE--------------LQSEMKSTNGID-------- 845
PkHsp90_A 798 TYLMNVQGNLDDKPSESSSSSSSEQASNSAQGNSTLDSTGEKSPPLDNPNSSSESLGLSEPNADASS 864
PvHsp90_A 796 TYLMNVQGNLDSTPSESSPSSSSEPAQNSAQGDSTQGNAGGSNPALDNPSSSSESAGLSEPSADASS 862
PyHsp90_A 793 AYLKDVQDDIDITPSNNSEDTTL---DNTTPND--------------KSQLNSEQSDIN-------- 834

PbHsp90_A 835 ------------------------------------------------QNDSISNNTLQES------ 847
PfHsp90_A 846 ------------------------------------------------DNSNISENKINESSSNQNN 864
PkHsp90_A 865 EGGPGEERLTASNLDGLDGDNANANANANANASASTSASTSASTSASANASNISADALRDS------ 925
PvHsp90_A 863 TGGFGEERLSASNLDGLGGDNASANANANESSNA--------------STNSIGANGLSDS------ 909
PyHsp90_A 835 ------------------------------------------------QNDSISNNTLQES------ 847

PbHsp90_A 848 ----------------------------NENN-SENYINGNPQEAGSEVSNDKQFDDS-STIE 880
PfHsp90_A 865 IGENSIAEENNIKNIAESDVNKINLGENDVSQNTMHKQDSGLFNLDPSILNSNMLSGSDKTLL 927
PkHsp90_A 926 ----------------------------TLNGNNMNSLDSNLYNIDRSIFNDKMFSGSDKTIL 960
PvHsp90_A 910 ----------------------------ALNGSNMNGLDPNLYNIDRNIFNDKMFSGSDKTVL 944
PyHsp90_A 848 ----------------------------NENN-SENYISGNPQEAGSEVSDDKQFDDS-NANE 880
```

ENDOPLASMIC RETICULUM HSP90

SEQUENCE ALIGNMENT

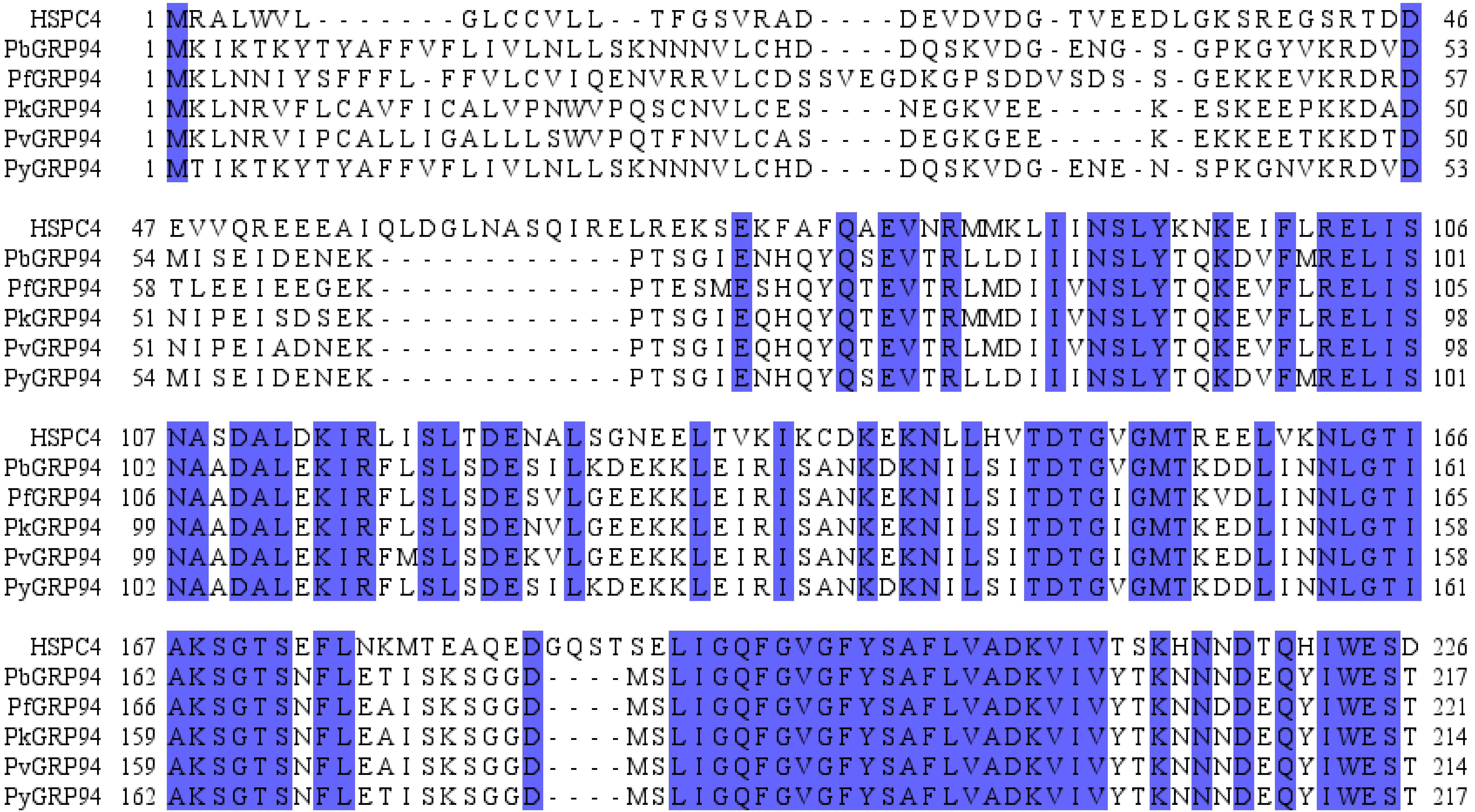
HSPC4 1 MRALWVL-------GLCCVLL--TFGSVRAD----DEVDVDG-TVEEDLGKSREGSRTDD 46
PbGRP94 1 MKIKTKYTYAFFVFLIVLNLLSKNNNVLCHD----DQSKVDG-ENG-S-GPKGYVKRDVD 53
PfGRP94 1 MKLNNIYSFFFL-FFVLCVIQENVRRVLCDSSVEGDKGPSDDVSDS-S-GEKKEVKRDRD 57
PkGRP94 1 MKLNRVFLCAVFICALVPNWVPQSCNVLCES----NEGKVEE-----K-ESKEEPKKDAD 50
PvGRP94 1 MKLNRVIPCALLIGALLLSWVPQTFNVLCAS----DEGKGEE-----K-EKKEETKKDTD 50
PyGRP94 1 MTIKTKYTYAFFVFLIVLNLLSKNNNVLCHD----DQSKVDG-ENE-N-SPKGNVKRDVD 53
HSPC4 47 EVVQREEEAIQLDGLNASQIRELREKSEKFAFQAEVNRMMKLIINSLYKNKEIFLRELIS 106
PbGRP94 54 MISEIDENEK------------PTSGIENHQYQSEVTRLLDIIINSLYTQKDVFMRELIS 101
PfGRP94 58 TLEEIEEGEK------------PTESMESHQYQTEVTRLMDIIVNSLYTQKEVFLRELIS 105
PkGRP94 51 NIPEISDSEK------------PTSGIEQHQYQTEVTRMMDIIVNSLYTQKEVFLRELIS 98
PvGRP94 51 NIPEIADNEK------------PTSGIEQHQYQTEVTRLMDIIVNSLYTQKEVFLRELIS 98
PyGRP94 54 MISEIDENEK------------PTSGIENHQYQSEVTRLLDIIINSLYTQKDVFMRELIS 101
HSPC4 107 NASDALDKIRLISLTDENALSGNEELTVKIKCDKEKNLLHVTDTGVGMTREELVKNLGTI 166
PbGRP94 102 NAADALEKIRFLSLSDESILKDEKKLEIRISANKDKNILSITDTGVGMTKDDLINNLGTI 161
PfGRP94 106 NAADALEKIRFLSLSDESVLGEEKKLEIRISANKEKNILSITDTGIGMTKVDLINNLGTI 165
PkGRP94 99 NAADALEKIRFLSLSDENVLGEEKKLEIRISANKEKNILSITDTGIGMTKEDLINNLGTI 158
PvGRP94 99 NAADALEKIRFMSLSDEKVLGEEKKLEIRISANKEKNILSITDTGIGMTKEDLINNLGTI 158
PyGRP94 102 NAADALEKIRFLSLSDESILKDEKKLEIRISANKDKNILSITDTGVGMTKDDLINNLGTI 161
HSPC4 167 AKSGTSEFLNKMTEAQEDGQSTSELIGQFGVGFYSAFLVADKVIVTSKHNNDTQHIWESD 226
PbGRP94 162 AKSGTSNFLETISKSGGD----MSLIGQFGVGFYSAFLVADKVIVYTKNNNDEQYIWEST 217
PfGRP94 166 AKSGTSNFLEAISKSGGD----MSLIGQFGVGFYSAFLVADKVIVYTKNNDDEQYIWEST 221
PkGRP94 159 AKSGTSNFLEAISKSGGD----MSLIGQFGVGFYSAFLVADKVIVYTKNNNDEQYIWEST 214
PvGRP94 159 AKSGTSNFLEAISKSGGD----MSLIGQFGVGFYSAFLVADKVIVYTKNNNDEQYIWEST 214
PyGRP94 162 AKSGTSNFLETISKSGGD----MSLIGQFGVGFYSAFLVADKVIVYTKNNNDEQYIWEST 217

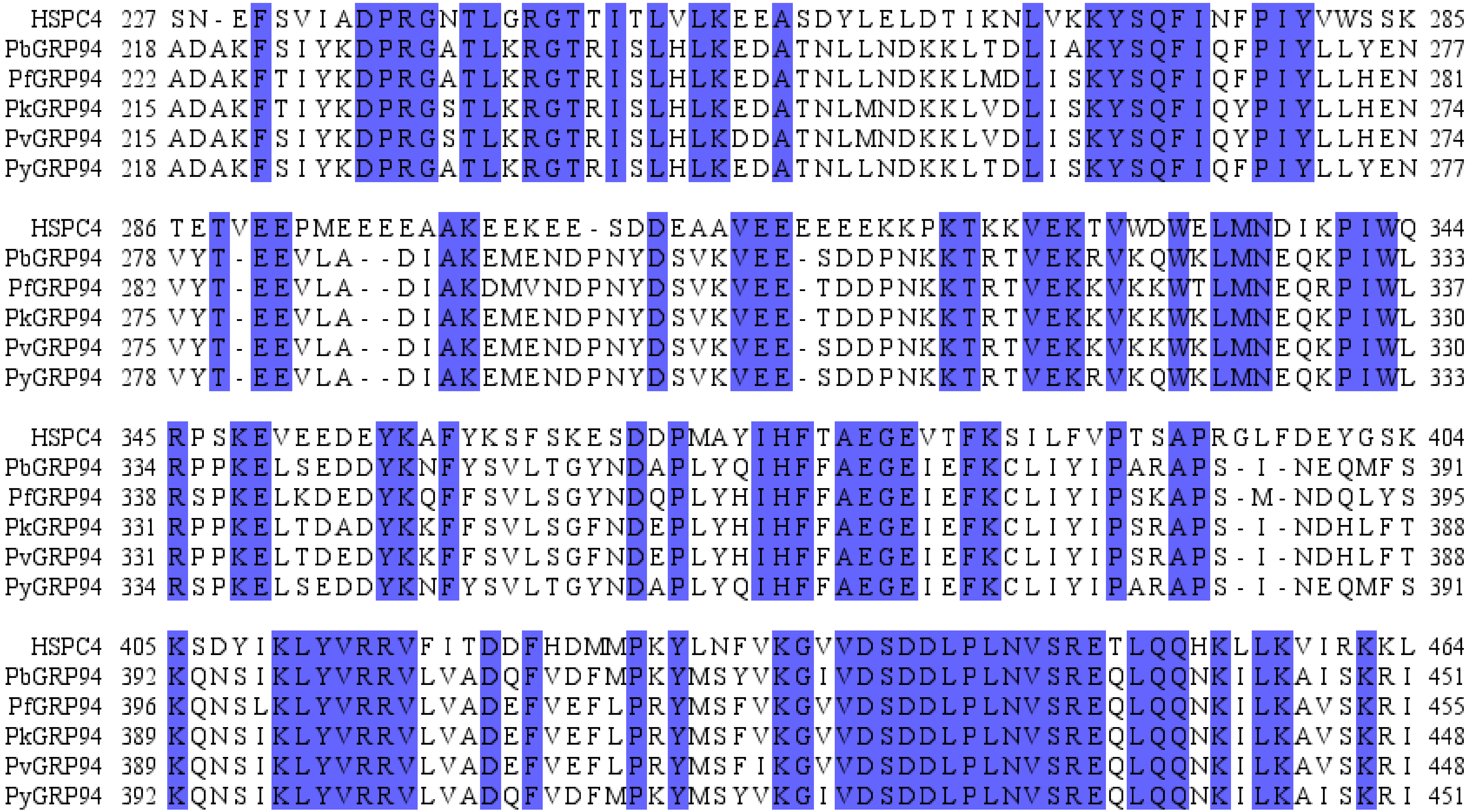
HSPC4 227 SN-EFSVIADPRGNTLGRGTTITLVLKEEASDYLELDTIKNLVKKYSQFINFPIYVWSSK 285
PbGRP94 218 ADAKFSIYKDPRGATLKRGTRISLHLKEDATNLLNDKKLTDLIAKYSQFIQFPIYLLYEN 277
PfGRP94 222 ADAKFTIYKDPRGATLKRGTRISLHLKEDATNLLNDKKLMDLISKYSQFIQFPIYLLHEN 281
PkGRP94 215 ADAKFTIYKDPRGSTLKRGTRISLHLKEDATNLMNDKKLVDLISKYSQFIQYPIYLLHEN 274
PvGRP94 215 ADAKFSIYKDPRGSTLKRGTRISLHLKDDATNLMNDKKLVDLISKYSQFIQYPIYLLHEN 274
PyGRP94 218 ADAKFSIYKDPRGATLKRGTRISLHLKEDATNLLNDKKLTDLISKYSQFIQFPIYLLYEN 277
HSPC4 286 TETVEEPMEEEEAAKEEKEE-SDDEAAVEEEEEEKKPKTKKVEKTVWDWELMNDIKPIWQ 344
PbGRP94 278 VYT-EEVLA--DIAKEMENDPNYDSVKVEE-SDDPNKKTRTVEKRVKQWKLMNEQKPIWL 333
PfGRP94 282 VYT-EEVLA--DIAKDMVNDPNYDSVKVEE-TDDPNKKTRTVEKKVKKWTLMNEQRPIWL 337
PkGRP94 275 VYT-EEVLA--DIAKEMENDPNYDSVKVEE-TDDPNKKTRTVEKKVKKWKLMNEQKPIWL 330
PvGRP94 275 VYT-EEVLA--DIAKEMENDPNYDSVKVEE-SDDPNKKTRTVEKKVKKWKLMNEQKPIWL 330
PyGRP94 278 VYT-EEVLA--DIAKEMENDPNYDSVKVEE-SDDPNKKTRTVEKRVKQWKLMNEQKPIWL 333
HSPC4 345 RPSKEVEEDEYKAFYKSFSKESDDPMAYIHFTAEGEVTFKSILFVPTSAPRGLFDEYGSK 404
PbGRP94 334 RPPKELSEDDYKNFYSVLTGYNDAPLYQIHFFAEGEIEFKCLIYIPARAPS-I-NEQMFS 391
PfGRP94 338 RSPKELKDEDYKQFFSVLSGYNDQPLYHIHFFAEGEIEFKCLIYIPSKAPS-M-NDQLYS 395
PkGRP94 331 RPPKELTDADYKKFFSVLSGFNDEPLYHIHFFAEGEIEFKCLIYIPSRAPS-I-NDHLFT 388
PvGRP94 331 RPPKELTDEDYKKFFSVLSGFNDEPLYHIHFFAEGEIEFKCLIYIPSRAPS-I-NDHLFT 388
PyGRP94 334 RSPKELSEDDYKNFYSVLTGYNDAPLYQIHFFAEGEIEFKCLIYIPARAPS-I-NEQMFS 391
HSPC4 405 KSDYIKLYVRRVFITDDFHDMMPKYLNFVKGVVDSDDLPLNVSRETLQQHKLLKVIRKKL 464
PbGRP94 392 KQNSIKLYVRRVLVADQFVDFMPKYMSYVKGIVDSDDLPLNVSREQLQQNKILKAISKRI 451
PfGRP94 396 KQNSLKLYVRRVLVADEFVEFLPRYMSFVKGVVDSDDLPLNVSREQLQQNKILKAVSKRI 455
PkGRP94 389 KQNSIKLYVRRVLVADEFVEFLPRYMSFVKGVVDSDDLPLNVSREQLQQNKILKAVSKRI 448
PvGRP94 389 KQNSIKLYVRRVLVADEFVEFLPRYMSFIKGVVDSDDLPLNVSREQLQQNKILKAVSKRI 448
PyGRP94 392 KQNSIKLYVRRVLVADQFVDFMPKYMSYVKGIVDSDDLPLNVSREQLQQNKILKAISKRI 451

```
  HSPC4 465 VRKTLDMIKK-------------------------------IADDKYNDTFWKEFGTNIK 493
PbGRP94 452 VRKILDTFRKLYLDGKKNKDSLREQLEKETDDDKKKEIQKKLNEPSVYKSIYKEYRKFFK 511
PfGRP94 456 VRKILDTFHKLYKEGKKNKETLRSELENETDEEKKKEITKKLSEPSTYKLIYKEYRKFLK 515
PkGRP94 449 VRKILDTFRTLYLNGKKNKEELRAELAKETDEEKKKDIQKKINEPSTYKLIYKEYRKYLK 508
PvGRP94 449 VRKILDTFRTLYTSGKKNKEDLRAQLAKETDEEKKKEIQKKINEPSTYKLIYKEYRKYLK 508
PyGRP94 452 VRKILDTFRKLYLDGKKNKDSLREQLEKETDEDKKKEIQKKLSEPSVYKSIYKEYRKFFK 511

  HSPC4 494 LGVIEDHSNRTRLAKLLRFQSSHHPTDITSLDQYVERMKEKQDKIYFMAGSSRKEAESSP 553
PbGRP94 512 SGCYEDDINRSKIVKLLLFKTMLHPKSI-SLDTYVENMKPDQKFIYYASGESYEYLSKIP 570
PfGRP94 516 SGCYEDDINRNKIAKLLLFKTMQYPKSI-SLDTYIEHMKPDQKFIYYASGDSYEYLAKIP 574
PkGRP94 509 TGCYEDDINRNKIVKLLLFKTMLHPKSI-SLDTYIENMKPDQKFIYYASGESYEYLSKIP 567
PvGRP94 509 TGCYEDDINRSKIVKLLLFKTMLHPKSI-SLDTYVENMKPDQKFIYYASGDSYEYLSKIP 567
PyGRP94 512 SGCYEDDINRSKIVKLLLFKTMLHPKSI-SLDTYVENMKPDQKFIYYASGESYEYLSKIP 570

  HSPC4 554 FVERLLKKGYEVIYLTEPVDEYCIQALPEFDGKRFQNVAKEGVKFDESEKTKESREAVEK 613
PbGRP94 571 QLQIFKKKNIDVLFLTESVDESCIQRVEDYDGKKFKSIQKGEITFDLTEEEKKREEKVKK 630
PfGRP94 575 QLQIFKKKNIDVLFLTESVDESCIQRVQEYEGKKFKSIQKGEISFELTEEEKKKEQQMQK 634
PkGRP94 568 QLQIFKKKNIDVVFLTESVDESCVQRVQEYDGKKFKSIQKGEITFDLTEDEKKKEEKVKK 627
PvGRP94 568 QLQIFKKKNIDVVFLTESVDESCVQRVQEYDGKKFKSIQKGEITFELTDDEKKKEEKVKK 627
PyGRP94 571 QLQIFKKKNIDVLFLTESVDESCIQRVEDYDGKKFKSIQKGEITFDLTEEEKKREEKVKK 630

  HSPC4 614 EFEPLLNWMKDKALKDKIEKAVVSQRLTESPCALVASQYGWSGNMERIMKAQAYQTGKDI 673
PbGRP94 631 MYTALIDVISD-TLRNKIFKVEISRRLVDAPCAVVSTEWGLSGQMEKLMKININNNDQ-V 688
PfGRP94 635 MYKALIDVISD-TLKNQIFKVEISRRLVDAPCAVVSTEWGLSGQMEKLMKMNVSNSDQ-I 692
PkGRP94 628 MYKALIDVISD-TLRNQIFKVEISRRLVDAPCAVVSTEWGLSGQMEKLMKINVNNSDQ-I 685
PvGRP94 628 MYKALIDVISD-TLRNQIFKVEISRRLVDAPCAVVSTEWGLSGQMEKLMKINVNNADQ-I 685
PyGRP94 631 MYTALIDVISD-TLRNKIFKVEISRRLVDAPCAVVSTEWGLSGQMEKLMKININNTDQ-V 688
```

```
HSPC4   674 STNYYASQKKTFEINPRHPLIRDMLRRIKEDEDDKTVLDLAVVLFETATLRSGYLLPDTK 733
PbGRP94 689 ---KAMSGQKILEINPDHPIMIDLLKRSVENPKDPELVESIKVIYQSAKLASGFDLEDTS 745
PfGRP94 693 ---KAMSGQKILEINPNHPIMIDLLKRSVTNPKDLELTNSIKIMYQSAKLASGFDLEDTA 749
PkGRP94 686 ---RAMSGQKILEINPDHPIMIDLLKRSVSNPKDSQLTESIKIIYQSAKLASGFDLEDTA 742
PvGRP94 686 ---KAMSGQKILEINPDHPIMIDLLKRSVSNPKDSQLTESIKIIYQSAKLASGFDLEDTA 742
PyGRP94 689 ---KAMSGQKILEINPDHPIMIDLLKRSVENPKDPELVESIKVIYQSAKLASGFDLEDTS 745

HSPC4   734 AYGDRIERMLRLSLNIDPDAKVEEEPEEEPEETAEDTTEDT--EQDEDEEMDVGTD-EEE 790
PbGRP94 746 DLAQIVYDHINQTLGVDNTLKIDDLDPA-IFETKKIDDQN-------VEEINIDDAIKKG 797
PfGRP94 750 DLAQIVYDHINQKLGVDNNLKIDDLDPS-IFETKKIEDEN--DSSKFEEEINIDDEIQKK 806
PkGRP94 743 DLAQIVYDHINQKLGVDNNLKIDDLDPA-IFETKKLEQEDSGDGQKFHEEINIDDEIQKQ 801
PvGRP94 743 DLAQIVYDHINQKLGVDNNLKIDDLDPA-IFETKKMEQEDSPDGQKFHEEINIDDEIQKQ 801
PyGRP94 746 DLAQIVYDHINQTLGVDNTLKIDDLDPA-IFETKKIDDQN-------VEEINIDDAIKKG 797

HSPC4   791 ETA--KESTAEKDEL 803
PbGRP94 798 DSN----SESKNDEL 808
PfGRP94 807 DNNVDNESNDKSDEL 821
PkGRP94 802 DSA--TESSSKNDEL 814
PvGRP94 802 DAA--PEAAPKNDEL 814
PyGRP94 798 DSN----SESKNDEL 808
```

MITOCHONDRIA HSP90 SEQUENCE ALIGNMENT

```
HSPC5     1 MARE--LRALLLWGRRLRPLLRAPALAAVPGG-KP------ILCP-----------R  37
PbTRAP1   1 MSLSKLSRASLQLIKG-------SSVLENNGR-NKIGKFQFTRCMNTK--C----VL  43
PfTRAP1   1 MSFSKFMKCSTQLSRR-------LSNFEGKGTFNKSAF---YNCTREK--CSIVCLR  45
PkTRAP1   1 MSLSKFARSTLQINKT-------CGVVEAQTK-NKAAA---ATCRGLRKISTGSHVY  46
PvTRAP1   1 MSLSKFARSTLQINKA-------CGVVEAQAK-SKTAA---ATCRGLRKISSGNNVQ  46
PyTRAP1   1 MSLSKLSKKSLQLIKG-------SSVLENNGR-NKIGNLQFVRCMNTK--C----VL  43

HSPC5    38 RTTAQLG-PRRNPAWSLQAGRLFSTQTAEDKEEPLHSIISSTESVQGSTSKHEFQAE  93
PbTRAP1  44 NKNIWNG--KKKNEYNLEYKRLFS-----------------------TSENYEFKAE  75
PfTRAP1  46 KKMNVEL--KKICEISKMNKRNYS----------------------SECENYEFKAE  78
PkTRAP1  47 NKWYAQLIAKELSRR-GGLVRSFS----------------------SNGESYEFKAE  80
PvTRAP1  47 NKWYAQLIAKELSHR-GGLVKHFS----------------------TAGESYEFKAE  80
PyTRAP1  44 NKNIWNG--KKKDEYNLEYKRLFS-----------------------TCENYEFKAE  75

HSPC5    94 TKKLLDIVARSLYSEKEVFIRELISNASDALEKLRHKL-VSDGQA------------ 137
PbTRAP1  76 TKKLLQIVAHSLYTDKEVFIRELISNSSDAIEKLRFMQ-TASIKDVDPNNKAEGNII 131
PfTRAP1  79 TKKLLQIVAHSLYTDKEVFIRELISNSSDAIEKLRFLLQSGNIKA------------ 123
PkTRAP1  81 TKKLLQIVAHSLYTDKEVFIRELISNSSDALEKRRFTQ-TASIRSVDDTVAN----- 131
PvTRAP1  81 TKKLLQIVAHSLYTDKEVFIRELISNSSDALEKRRFTQ-TASIKRVDDTTAS----- 131
PyTRAP1  76 TKKLLQIVAHSLYTDKEVFIRELISNSSDAIEKLRFTQ-TASIKDVDPNNKTEGNII 131

HSPC5   138 -LPE--MEIHLQTNAEKGTITIQDTGIGMTQEELVSNLGTIARSGSKAFLDALQNQA 191
PbTRAP1 132 ENTEQPFYIKVSTNDKDKLFIIEDNGIGMNKTEVIENLGTIAKSGSQNFINALKEKG 188
PfTRAP1 124 -SENITFHIKVSTDENNNLFIIEDSGVGMNKEEIIDNLGTIAKSGSLNFLKKLKEQK 179
PkTRAP1 132 EAGEIPLHIKVSADAKKNLFIIEDSGIGMNKEEVIENLGTIAKSGSLNFLNALKERS 188
PvTRAP1 132 ETAEIPLHIKVSADAKKNLFIIEDSGIGMNKEEVIENLGTIAKSGSLNFLNALKERS 188
PyTRAP1 132 EDKEQPFYIKISTNDKDKLFIIEDNGIGMNKTEVIENLGTIAKSGSQNFINALKEKG 188
```

```
  HSPC5  192 EA---------------------------------SS-KIIGQFGVGFYSAFMVADR 214
PbTRAP1  189 EL----------------------------NKNSQTT-DIIGQFGVGFYSTFVVSDF 216
PfTRAP1  180 ESINRNNNINKNEIDNKINNNNNMDVPIEGNEKSQEG-DIIGQFGVGFYSSFVVSNK 235
PkTRAP1  189 RNATEES-------------KNSSDPSGEKSEISQPGDNIIGQFGVGFYSSFVVSDE 232
PvTRAP1  189 SSASEES-------------KKSPEQSGERGEISKPGDNIIGQFGVGFYSSFVVSDQ 232
PyTRAP1  189 ES----------------------------NQNSQTT-DIIGQFGVGFYSTFVVSDS 216

  HSPC5  215 VEVYSRSAAPG-SLGYQWLSDGSGVFEIAEASGVRTGTKIIIHLKSDCKEFSSEARV 270
PbTRAP1  217 VEVFTKSHEKG-SIGYHWKSDGNGKFTLTEDNSIERGTKIICHLKEACSEFSNINKV 272
PfTRAP1  236 VEVFTRSYDNNSSKGYHWVSYGNGTFTLKEVDNIPKGTKIICHLKDSCKEFSNIQNV 292
PkTRAP1  233 VEVFTRSHDAN-SVGYHWKSDGNGTFTLKEVEDLPRGTKIVCHLKESCKEFSNIHRV 288
PvTRAP1  233 VEVFTRSHDAN-SVGYHWKSDGNGTFTLKEVEDLPRGTKIVCHLKDSCKEFANIHRV 288
PyTRAP1  217 VEVFTKSHEEG-SIGYHWKSDGNGKFTITEDNSIKRGTKIVCHLKEACSEFSNINKI 272

  HSPC5  271 RDVVTKYSNFVSFPLYLNGRR------------------------------------ 291
PbTRAP1  273 QTIVEKFSSFINFPVYILNKKQTPIEPKSVS-------------------------- 303
PfTRAP1  293 QKIVEKFSSFINFPVYVLKKKKILQTRKDIEQNELNDHTQQNELNHHNDQNDHTQQN 349
PkTRAP1  289 QEIVEKFSSFINFPVYIVNRKKDVKGSSRLVKGDDMQNEESSS-------------- 331
PvTRAP1  289 QEIVEKFSSFINFPVYIVNRKKDIKGSSRLVKGDDAAHEEASPP------------- 332
PyTRAP1  273 QTIVEKFSSFINFPVYILNKKQAPLESASAD-------------------------- 303

  HSPC5      ---------------------------------------------------------
PbTRAP1  304 ------------------------------------------------------ES- 305
PfTRAP1  350 ELNHHNDQNDHTQQNELNHHNDQNDHTQQNELNHHVEHHKNVVENKNDEYEKNMDNR 406
PkTRAP1  332 --------------------------LPQEGL------------HKDVEKEKAMDGT 350
PvTRAP1  333 --------------------------QAEEGL------------LNEAEREKAMEGV 351
PyTRAP1  304 ---------------------------------------------NAASADNAVNG- 314
```

```
  HSPC5  292 ---------------------------------------------MNTLQAIWMMDP 303
PbTRAP1  306 --DKV------------------GESDKVGESDKVSEIEQVQEILINSQKPLWCKD- 341
PfTRAP1  407 TSDNIKPESLSKQNDYENMNQCNNEETVLNRTKELNEEHIVEEILVNKQKPLWCKD- 462
PkTRAP1  351 --TEV------------------EDSTTAADTSKGETESLTDEVQINNQKPLWCKE- 386
PvTRAP1  352 A-DGV------------------TDATASADAPKGDAESLTDEVQINNQKPLWCKD- 388
PyTRAP1  315 --DNV------------------DNAANTANADNAGESEQVQEILINSQKPLWCKD- 350

  HSPC5  304 KDVREWQHEEFYRYVAQAH------DKPRYTLHYKTDAPLNIRSIFYVPDMKPS-MF 353
PbTRAP1  342 -EVSEEEHKKFFNFLNKNKSYSDDNKSYLYKLMYKTDAPMSIKSVFYIPEEAPSRLF 397
PfTRAP1  463 -NVTEEEHRHFFHFLNKNKSYNEDNKSYLYNMLYKTDAPLSIKSVFYIPEEAPSRLF 518
PkTRAP1  387 -NVTEEEHNKFFKFLTKNKNYGD-DKGYVVYKVLYKTDAPMSIKSVFYIPEEAPSRLF 441
PvTRAP1  389 -HVTEEEHEKFFKFLTKKKSYDD-DKGYVVYKVLYKTDAPMSIKSVFYIPEEAPSRLF 443
PyTRAP1  351 -EVSEEEHKKFFNFLNKNKSYSDENKSYLYKLMYKTDAPMSIKSVFYIPEEAPSRLF 406

  HSPC5  354 DVSRELGSSVALYSRKVLIQTKATDILPKWLRFIRGVVDSEDIPLNLSRELLQESAL 410
PbTRAP1  398 QQNYDI--DVSLYCKKVLVKKCADNIIPKWLHFVKGIIDCEDMPLNISRESMQNSTL 452
PfTRAP1  519 QQSNDI--EISLYCKKVLVKKNADNIIPKWLYFVKGVIDCEDMPLNISRENMQDSSL 573
PkTRAP1  442 QQSNEI--EVSLYCKKVLVKKNADNIIPKWLHFVKGVIDCEDMPLNISRENMQDSTL 496
PvTRAP1  444 QQSNEI--EVSLYCKKVLVKKSADNIIPKWLHFVKGVIDCEDMPLNISRENMQDSTL 498
PyTRAP1  407 QQNYDI--DVSLYCKKVLVKKCADNIIPKWLHFVKGIIDCEDMPLNISRESMQDTAL 461

  HSPC5  411 IRKLRDVLQQRLIKFFIDQSKKDAEKYAKFFEDYGLFMREGIVTAT-EQEVKEDIAK 466
PbTRAP1  453 MSKLSRIIVTKILKTLEKEASIDEDKYLKFYKNFSYNLKEGILEDSTKNMYKNVMMN 509
PfTRAP1  574 INKLSRVVVSKILKTLEREADINEEKYLKFYKNYNYNLKEGVLEDSNKNHYKNSLMN 630
PkTRAP1  497 INKISRVVVTKILKTLEKESTNDEDKYRKFYTNYSYYLKEGVLEDSSNNFYKNTLMN 553
PvTRAP1  499 ISKISRVVVTKILKTLEKEATNDEEKYRKFYANYSYYLKEGVLEDSSNQFYKGMLMN 555
PyTRAP1  462 MNKLSRIIVTKIIKTLEKEASINEDKYLKFYKNFSYNLKEGILEDSTKNMYKNVMLN 518
```

```
  HSPC5 467 LLRYESSALPSGQLTSLSEYASRMRAGTRNIYYLCAPNRHLAEHSPYYEAMKKKDTE 523
PbTRAP1 510 LLRFYSVN--LKKYISINEYIKNFKNTQKNIYYFSANDINIALSSPYMESFKNKQVD 564
PfTRAP1 631 LLRFYSIN--QNKFISLKQYVNNFRNNQKNIYYFSANDKNVALNSPYMEPFKKQNID 685
PkTRAP1 554 LLRFYSIE--QRKNISLKDYVANFKNSQKKIYYFCVNELNVALSSPYMEPFKKQGVD 608
PvTRAP1 556 LLRFYSIQ--QRKNIPLKEYVANFKKAQKKIYYFCVNDLDVALSSPYMEPFKKQGVD 610
PyTRAP1 519 LLRFYSVN--LKKYISLNEYVKNFKNTQKNIYYFSANDINIALSSPYMESFKNKQVD 573

  HSPC5 524 VLFCFEQFDELTLLHLREFDKKKLISVETDIVVDHYKEEKFE--------------- 565
PbTRAP1 565 VLLLFEEIDEFVLMNLQSYKDSKFISIDSSQKEDLDEIILNNENTD-MNTAN---TN 617
PfTRAP1 686 VLLLLEEIDEFVLMNLQTYKDAKFVSIDTSQNEDFDEAVLNTNKND----------- 731
PkTRAP1 609 VLLLFEEIDEFVLMNLQEFNGAKFVSIDSSQNEDFDQELLNSNRAD-GAKA----GD 660
PvTRAP1 611 VLLLFEEIDEFVLMNLQEFNGSKFVSIDSSQNEDFDPELLNANRGDDGAKG----GD 663
PyTRAP1 574 VLLLFEEIDEFVLMNLQSYNDSKFVSIDSSQKEDLDEIILSNENSD-GKNSDGKNGD 629

  HSPC5 566 -----DRSP-AAECLSEKETEELMAWMRNVLGSRVTNVKVTLRLDTHPAMVTVLEMG 616
PbTRAP1 618 NNNNSDSNN-NKIPFTQDQKLELEKYIKQVLGPKCSDVKFSERLSLSPAVVTGFLSP 673
PfTRAP1 732 -----NEKK-QSIFFNDEQKKELQAYFKQVLGSKCSDVKFSERLTTSPAVVTGFLSP 782
PkTRAP1 661 KDEASDEAR-KNGIFNDSQKSELAKYFKDVLGSKCSDVKFSDRLTESPAVVTGFLSP 716
PvTRAP1 664 KEEASNGGE-KNRIFNDSQKSELAKYFKDVLGSKCSDVKFSDRLTESPAVVTGFLSP 719
PyTRAP1 630 DQNSDGKNNMNKIPFTQDQKIELEKYIKQVLGPKCSDVKFSERLSLSPAVVTGFLSP 686

  HSPC5 617 AARHFLRMQQLAK-TQE--------ERAQLLQPTLEINPRHALIKKLNQLRASEPGL 664
PbTRAP1 674 TLRRVMKATMKNSDMNE--------NMLQNLPVTLEINPTHTIITSIYHLKNANEEV 722
PfTRAP1 783 TLRKVMKATMKNSDFNDNTNNSNNMNMFQNLPATLELNPSHTIVTSIYHLKNTNQEV 839
PkTRAP1 717 TLRKVMKATMKNSSFQDN-------NMLNNLPATLELNPSHTIVTSIFHLKNTNQDV 766
PvTRAP1 720 TLRKVMKATMKNASFQDN-------SMLHNLPATLELNPSHTIVTSIFHLKNTNQEV 769
PyTRAP1 687 TLRRVMKATMKNSDMND--------NMLQNLPATLEINPTHTIIISIYHLKNTNEEV 735
```

```
HSPC5    665 AQLLVDQIYENAMIAAGLVDDPRAMVGRLNELLVKAL-------------------- 701
PbTRAP1  723 AKLLIEQLYDNACIAAGILEDPRSLLTKLNELLLLTARYAYHYEKKNDACSESVDKT 779
PfTRAP1  840 AKLLVQQLYDNACIAAGILEDPRSLLSKLNELLLLTARYAYHYEKKDI-QENTKDQP 895
PkTRAP1  767 AKLLVQQLYDNACIAAGILEDPRSLLTKLNELLLLTARYAYHYEKNADGVSGVQEDP 823
PvTRAP1  770 AKLLVQQLYDNACIAAGILEDPRSLLTKLNELLLLTARYAYHYEKNTEGGPALQEEQ 826
PyTRAP1  736 AKLLVEQLYDNACIAAGILEDPRSLLTKLNELLLLTARYAYHYEKKTDAHSESVDKA 792

HSPC5        ---------------------------------------------------------
PbTRAP1  780 D------------------------------GNNINDSVNAN----V---------E 793
PfTRAP1  896 I--DVK-----------------------DNTMAIDNIVNTK----ENN--TNDDSV 921
PkTRAP1  824 S--PSE-----------------------PSAGNVDRTIPATEASPAQA--DDEENS 853
PvTRAP1  827 S--PSE----------------------------V---SQVS----ANG--AAEEGS 844
PyTRAP1  793 DAYPESVDKADAHPESVDKADAHSESVNKAEANNINDSASAN----AENVEEHDDST 845

HSPC5    702 ------ERH 704
PbTRAP1  794 KVEEHDDRF 802
PfTRAP1  922 MKEAQSSNL 930
PkTRAP1  854 AKEAHASKL 862
PvTRAP1  845 AKEAQASKL 853
PyTRAP1  846 LKEA--HKI 852
```

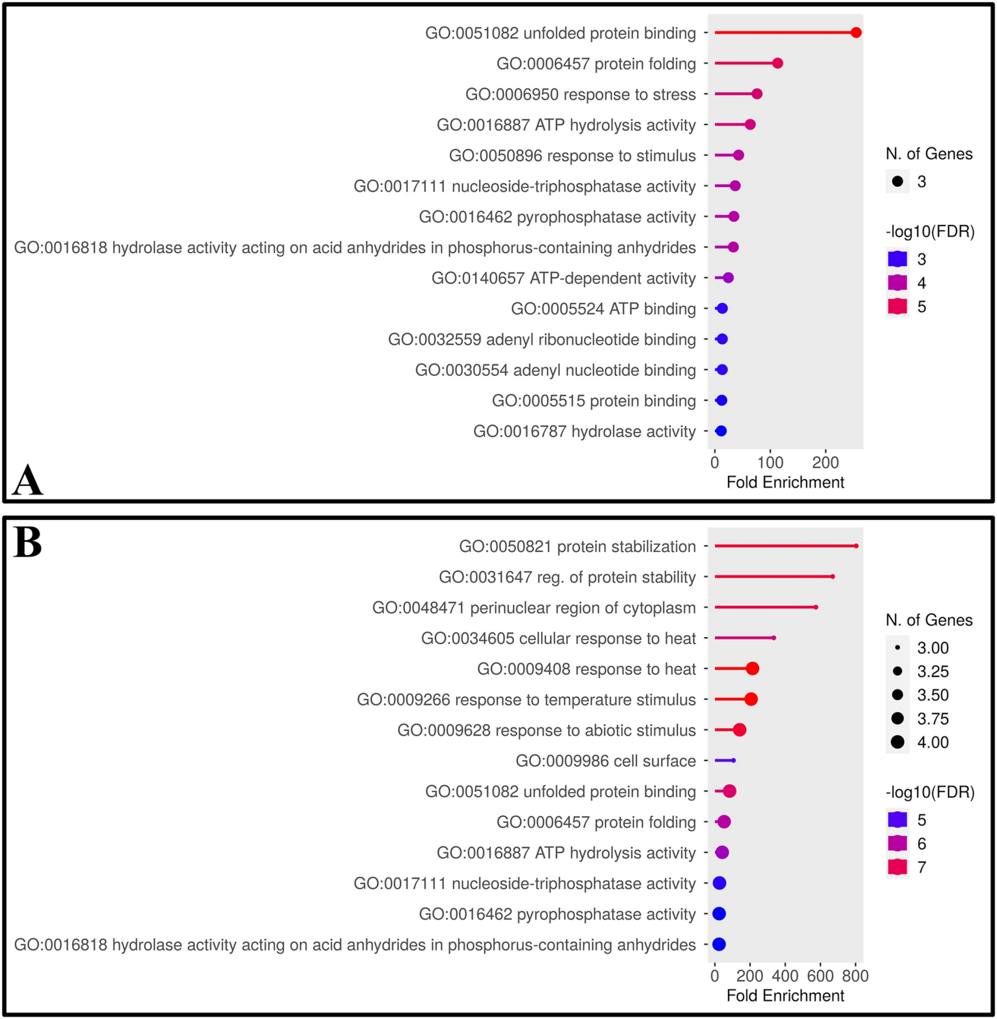
A
GO:0051082 unfolded protein binding
GO:0006457 protein folding
GO:0006950 response to stress
GO:0016887 ATP hydrolysis activity
GO:0050896 response to stimulus
GO:0017111 nucleoside-triphosphatase activity
GO:0016462 pyrophosphatase activity
GO:0016818 hydrolase activity acting on acid anhydrides in phosphorus-containing anhydrides
GO:0140657 ATP-dependent activity
GO:0005524 ATP binding
GO:0032559 adenyl ribonucleotide binding
GO:0030554 adenyl nucleotide binding
GO:0005515 protein binding
GO:0016787 hydrolase activity
0
100
200
Fold Enrichment
N. of Genes
3
-log10(FDR)
3
4
5
B
GO:0050821 protein stabilization
GO:0031647 reg. of protein stability
GO:0048471 perinuclear region of cytoplasm
GO:0034605 cellular response to heat
GO:0009408 response to heat
GO:0009266 response to temperature stimulus
GO:0009628 response to abiotic stimulus
GO:0009986 cell surface
GO:0051082 unfolded protein binding
GO:0006457 protein folding
GO:0016887 ATP hydrolysis activity
GO:0017111 nucleoside-triphosphatase activity
GO:0016462 pyrophosphatase activity
GO:0016818 hydrolase activity acting on acid anhydrides in phosphorus-containing anhydrides
0
200
400
600
800
Fold Enrichment
N. of Genes
3.00
3.25
3.50
3.75
4.00
-log10(FDR)
5
6
7

A

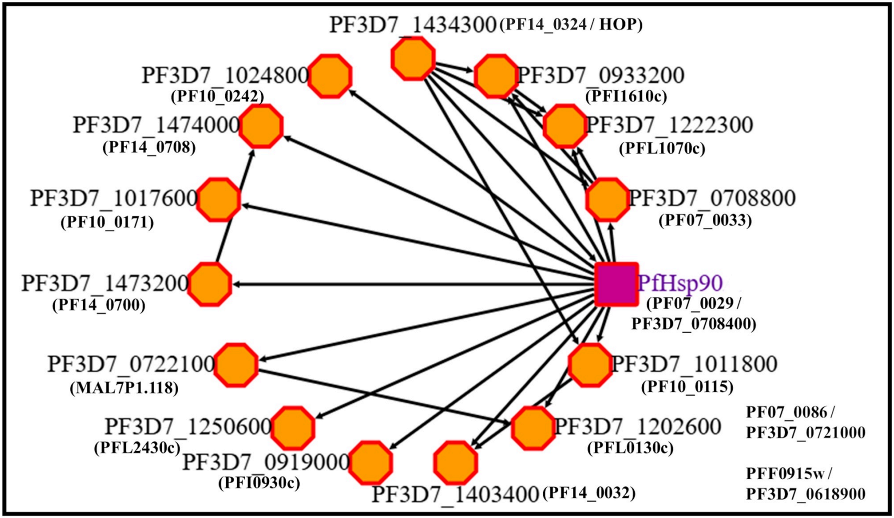

B

P. falciparum Acc. No. / Common Name	Putative Function
PF3D7_1202600	Unknown
PF3D7_1403400	Unknown
PF3D7_0919000/PfNAPS	Nucleosome assembly protein
PF3D7_1250600	Translation initiation factor eIF-2B subunit beta
PF3D7_0722100/PfPelo	Pelota-like protein; translation factor
PF3D7_0708800/PfHs70-z	Cytosolic heat shock protein 110 (Hsp110)
PF3D7_1473200	J-domain protein (JDP); essential type III JDP
PF3D7_1011800/PfPREBP	Pre-binding protein (PREBP); transcription factor
PF3D7_1024800/PfEXP3	Exported protein 3 (EXP3); involved in protein export
PK3D7_1222300/PkGRP94	Endoplasmic reticulum heat shock protein 90 (Hsp90)
PF3D7_1434300/PfHOP	Cytosolic Hsp70/Hsp90 organizing protein (HOP)
PF3D7_0933200	Calcyclin-binding protein
PF3D7_0618900.1	Phosphatidylinositol N-acetylglucosaminyltransferase subunit GPI1
PF3D7_0721000	Unknown

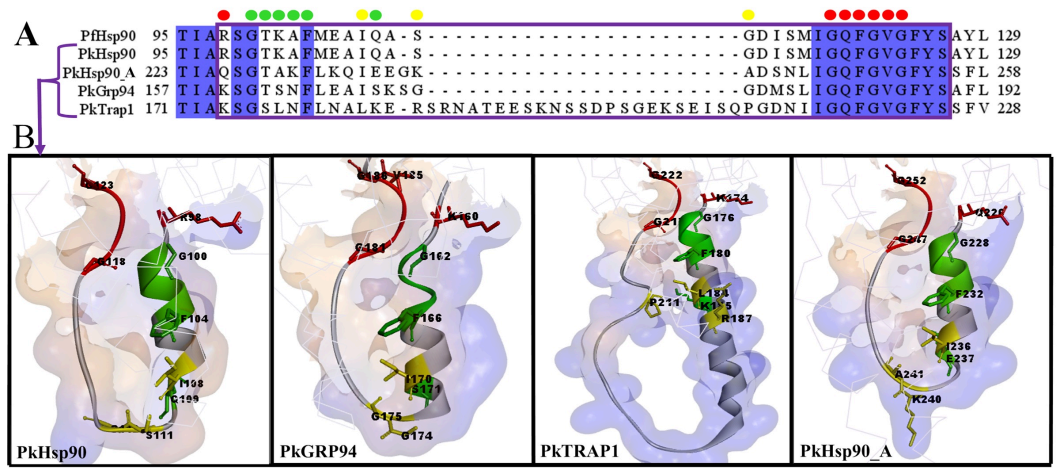
A
PfHsp90 95 TIARSGTKAFMEAIQA-S---------------------------GDISMIGQFGVGFYSAYL 129
PkHsp90 95 TIARSGTKAFMEAIQA-S---------------------------GDISMIGQFGVGFYSAYL 129
PkHsp90_A 223 TIAQSGTAKFLKQIEEGK---------------------------ADSNLIGQFGVGFYSSFL 258
PkGrp94 157 TIAKSGTSNFLEAISKSG---------------------------GDMSLIGQFGVGFYSAFL 192
PkTrap1 171 TIAKSGSLNFLNALKE-RSRNATEESKNSSDPSGEKSEISQPGDNIIGQFGVGFYSSFV 228
B
G123
R98
G118
G100
F104
I108
G109
S111
PkHsp90
V185
K160
G181
G162
F166
I170
S171
G175
G174
PkGRP94
G222
K174
G176
F180
P211
R187
PkTRAP1
G252
G228
F232
I236
E237
A241
K240
PkHsp90_A

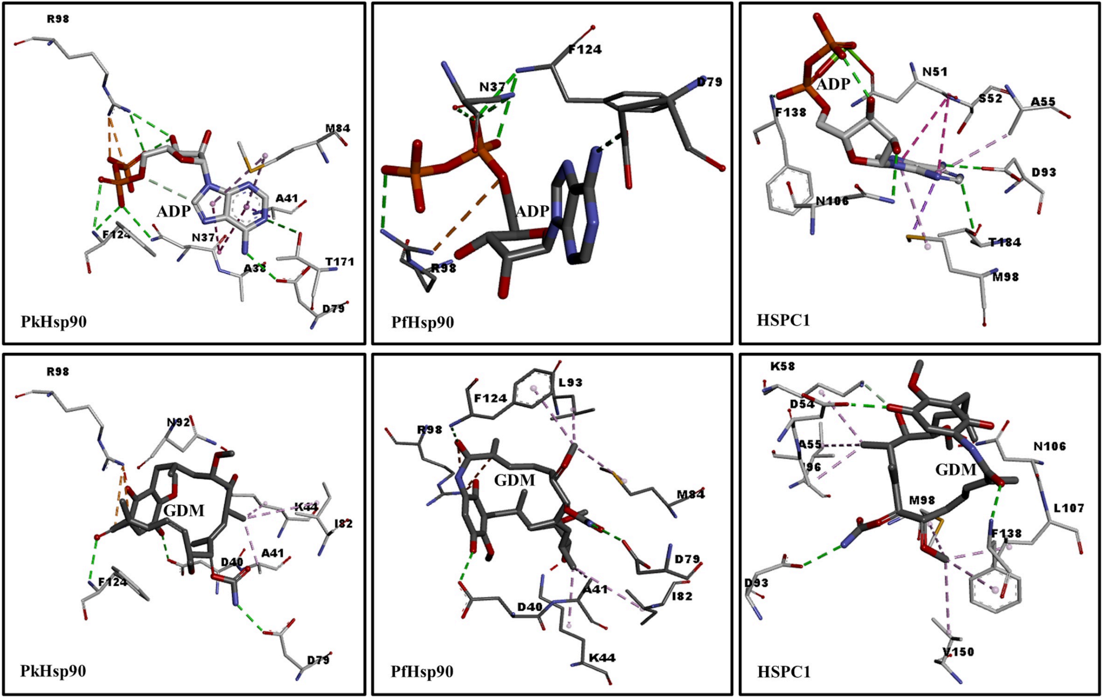

R98
M84
ADP
A41
F124
N37
T171
A38
D79
PkHsp90
F124
N37
D79
ADP
R98
PfHsp90
ADP
N51
S52
A55
F138
D93
N106
T184
M98
HSPC1
R98
N92
GDM
K44
I82
A41
D40
F124
D79
PkHsp90
L93
F124
R98
GDM
M84
D79
I82
A41
D40
K44
PfHsp90
K58
D54
A55
I96
N106
GDM
M98
L107
F138
D93
V150
HSPC1

Table S1: Pairwise Percentage Identity Matrix

	Localization	Mitochondrion					Apicoplast					Endoplasmic Reticulum					Cytosol					Control
**Localization**	**Hsp90s**	**PKNH_0915900**	**PF3D7_1118200**	**PBANKA_0929900**	**PVX_091545**	**PY17X_0931900**	**PKNH_1238400**	**PF3D7_1443900**	**PBANKA_1307800**	**PVX_118295**	**PY17X_1311600**	**PKNH_1441400**	**PF3D7_1222300**	**PBANKA_1437300**	**PVX_123745**	**PY17X_1439800**	**PKNH_0107000**	**PF3D7_0708400**	**PBANKA_0805700**	**PVX_087950**	**PY17X_0808800**	**HSPC1 (Hsp90α)**
Mitochondrion	**PKNH_0915900**	100	63.69	64.28	86.47	62.56	29.65	30.01	30.04	29.82	29.78	30.43	30.68	29.89	30.71	30.03	30.79	31.15	31.81	31.25	31.76	30.95
	**PF3D7_1118200**	63.69	100	65.84	62.94	63.94	30.34	30.49	31	30.65	31	29.63	30.15	29.77	29.36	29.63	29	28.89	29.51	28.63	29.75	29.51
	**PBANKA_0929900**	64.28	65.84	100	63.92	88.15	30.37	30.42	30.43	30.52	30.56	31.46	31.33	32.07	31.32	32.07	31.61	31.35	31.4	31.31	31.4	29.53
	**PVX_091545**	86.47	62.94	63.92	100	62.98	30.34	30.36	30.91	30.52	30.91	29.7	30.08	29.28	29.97	29.42	31.14	31.06	31.57	31.45	31.52	31
	**PY17X_0931900**	62.56	63.94	88.15	62.98	100	29.84	29.11	29.77	29.82	30.03	30.67	31.23	31.27	30.67	31.27	31.18	30.78	30.87	30.88	30.98	29.28
Apicoplast	**PKNH_1238400**	29.65	30.34	30.37	30.34	29.84	100	78.31	80.55	93.01	80.77	34.69	33.94	35.19	34.29	35.19	36.91	37.41	37.46	37.06	37.46	37.48
	**PF3D7_1443900**	30.01	30.49	30.42	30.36	29.11	78.31	100	80.21	79.36	79.74	35.52	34.38	34.82	34.99	34.69	37.91	37.96	38.31	38.05	38.31	37.88
	**PBANKA_1307800**	30.04	31	30.43	30.91	29.77	80.55	80.21	100	80.39	96.93	35.22	35.12	35.76	34.69	35.76	37.28	37.78	37.98	37.43	37.98	37.84
	**PVX_118295**	29.82	30.65	30.52	30.52	29.82	93.01	79.36	80.39	100	80.05	34.86	33.98	34.97	34.73	34.97	36.91	37.41	37.32	37.06	37.32	37.48
	**PY17X_1311600**	29.78	31	30.56	30.91	30.03	80.77	79.74	96.93	80.05	100	35.35	34.99	35.76	34.82	35.76	37.43	37.93	38.13	37.57	38.13	37.7
Endoplasmic Reticulum	**PKNH_1441400**	30.43	29.63	31.46	29.7	30.67	34.69	35.52	35.22	34.86	35.35	100	83.62	82.42	94.59	82.67	41.41	41.91	41.3	41.41	41.82	40.28
	**PF3D7_1222300**	30.68	30.15	31.33	30.08	31.23	33.94	34.38	35.12	33.98	34.99	83.62	100	79.83	82.51	80.07	41.24	41.16	41.13	41.24	41.51	39.72
	**PBANKA_1437300**	29.89	29.77	32.07	29.28	31.27	35.19	34.82	35.76	34.97	35.76	82.42	79.83	100	82.54	98.64	41.11	41.61	40.85	41.11	41.37	39.29
	**PVX_123745**	30.71	29.36	31.32	29.97	30.67	34.29	34.99	34.69	34.73	34.82	94.59	82.51	82.54	100	82.79	40.98	41.18	40.72	40.98	41.24	40
	**PY17X_1439800**	30.03	29.63	32.07	29.42	31.27	35.19	34.69	35.76	34.97	35.76	82.67	80.07	98.64	82.79	100	41.11	41.61	40.85	41.11	41.37	39.29
Cytosol	**PKNH_0107000**	30.79	29	31.61	31.14	31.18	36.91	37.91	37.28	36.91	37.43	41.41	41.24	41.11	40.98	41.11	100	94.25	94.42	97.7	94.29	67.18
	**PF3D7_0708400**	31.15	28.89	31.35	31.06	30.78	37.41	37.96	37.78	37.41	37.93	41.91	41.16	41.61	41.18	41.61	94.25	100	94.72	93.78	94.45	67.98
	**PBANKA_0805700**	31.81	29.51	31.4	31.57	30.87	37.46	38.31	37.98	37.32	38.13	41.3	41.13	40.85	40.72	40.85	94.42	94.72	100	94.29	98.61	67.75
	**PVX_087950**	31.25	28.63	31.31	31.45	30.88	37.06	38.05	37.43	37.06	37.57	41.41	41.24	41.11	40.98	41.11	97.7	93.78	94.29	100	94.44	67.61
	**PY17X_0808800**	31.76	29.75	31.4	31.52	30.98	37.46	38.31	37.98	37.32	38.13	41.82	41.51	41.37	41.24	41.37	94.29	94.45	98.61	94.44	100	68.22
**Control**	**HSPC1 (Hsp90α)**	30.95	29.51	29.53	31	29.28	37.48	37.88	37.84	37.48	37.7	40.28	39.72	39.29	40	39.29	67.18	67.98	67.75	67.61	68.22	100

All cytosolic isoforms are Hsp90 (e.g. PfHsp90/PF3D7_0708400)

All mitochondira isoforms are TRAP1 (e.g. PfTRAP1/PF3D7_1118200)

All endoplasmic reticulum isoforms are GRP94 (e.g. PfGRP94/PF3D7_1222300)

All apicoplast isoforms are Hsp90_A (e.g. PfHsp90_A/PF3D7_1443900)

Table S2: The Gene Ontology Enrichment of *P. falciparum* Hsp90 isoforms

Enrichment FDR	nGenes	Fold Enrichment	GO TERM	DESCRIPTION	GENES			
4.01E-08	3	803.7	GO:0050821	protein stabilization	PF3D7_0708400	PF3D7_1222300	PF3D7_1443900	
4.81E-08	3	669.75	GO:0031647	regulation of protein stability	PF3D7_0708400	PF3D7_1222300	PF3D7_1443900	
7.01E-08	3	574.07	GO:0048471	perinuclear region of cytoplasm	PF3D7_0708400	PF3D7_1222300	PF3D7_1443900	
3.30E-07	3	334.88	GO:0034605	cellular response to heat	PF3D7_0708400	PF3D7_1222300	PF3D7_1443900	
1.68E-08	4	214.32	GO:0009408	response to heat	PF3D7_0708400	PF3D7_1222300	PF3D7_1443900	PF3D7_1118200
1.68E-08	4	206.08	GO:0009266	response to temperature stimulus	PF3D7_0708400	PF3D7_1222300	PF3D7_1443900	PF3D7_1118200
4.14E-08	4	141	GO:0009628	response to abiotic stimulus	PF3D7_0708400	PF3D7_1222300	PF3D7_1443900	PF3D7_1118200
9.17E-06	3	105.75	GO:0009986	cell surface	PF3D7_0708400	PF3D7_1222300	PF3D7_1443900	
2.04E-07	4	83.72	GO:0051082	unfolded protein binding	PF3D7_0708400	PF3D7_1118200	PF3D7_1222300	PF3D7_1443900
1.06E-06	4	52.53	GO:0006457	protein folding	PF3D7_0708400	PF3D7_1118200	PF3D7_1222300	PF3D7_1443900
2.39E-06	4	41.86	GO:0016887	ATP hydrolysis activity	PF3D7_0708400	PF3D7_1118200	PF3D7_1222300	PF3D7_1443900
1.31E-05	4	26.26	GO:0017111	nucleoside-triphosphatase activity	PF3D7_0708400	PF3D7_1118200	PF3D7_1222300	PF3D7_1443900
1.48E-05	4	24.69	GO:0006950	response to stress	PF3D7_0708400	PF3D7_1222300	PF3D7_1443900	PF3D7_1118200
1.48E-05	4	24.24	GO:0016462	pyrophosphatase activity	PF3D7_0708400	PF3D7_1118200	PF3D7_1222300	PF3D7_1443900
1.48E-05	4	23.81	GO:0016818	hydrolase activity acting on acid anhydrides in phosphorus-containing anhydrides	PF3D7_0708400	PF3D7_1118200	PF3D7_1222300	PF3D7_1443900
1.48E-05	4	23.71	GO:0016817	hydrolase activity acting on acid anhydrides	PF3D7_0708400	PF3D7_1118200	PF3D7_1222300	PF3D7_1443900
3.71E-05	4	18.6	GO:0140657	ATP-dependent activity	PF3D7_0708400	PF3D7_1118200	PF3D7_1222300	PF3D7_1443900
0.00018	4	12.32	GO:0005524	ATP binding	PF3D7_0708400	PF3D7_1118200	PF3D7_1222300	PF3D7_1443900
0.00021	4	11.67	GO:0032559	adenyl ribonucleotide binding	PF3D7_0708400	PF3D7_1118200	PF3D7_1222300	PF3D7_1443900
0.00021	4	11.62	GO:0030554	adenyl nucleotide binding	PF3D7_0708400	PF3D7_1118200	PF3D7_1222300	PF3D7_1443900
0.00029	4	10.26	GO:0035639	purine ribonucleoside triphosphate binding	PF3D7_0708400	PF3D7_1118200	PF3D7_1222300	PF3D7_1443900
0.00032	4	9.8	GO:0032555	purine ribonucleotide binding	PF3D7_0708400	PF3D7_1118200	PF3D7_1222300	PF3D7_1443900
0.00032	4	9.74	GO:0017076	purine nucleotide binding	PF3D7_0708400	PF3D7_1118200	PF3D7_1222300	PF3D7_1443900
0.00032	4	9.71	GO:0032553	ribonucleotide binding	PF3D7_0708400	PF3D7_1118200	PF3D7_1222300	PF3D7_1443900
0.00032	4	9.64	GO:0050896	response to stimulus	PF3D7_0708400	PF3D7_1222300	PF3D7_1443900	PF3D7_1118200
0.00033	4	9.43	GO:0097367	carbohydrate derivative binding	PF3D7_0708400	PF3D7_1118200	PF3D7_1222300	PF3D7_1443900
0.00033	4	9.4	GO:0043168	anion binding	PF3D7_0708400	PF3D7_1118200	PF3D7_1222300	PF3D7_1443900
0.00039	4	8.8	GO:1901265	nucleoside phosphate binding	PF3D7_0708400	PF3D7_1118200	PF3D7_1222300	PF3D7_1443900
0.00039	4	8.8	GO:0000166	nucleotide binding	PF3D7_0708400	PF3D7_1118200	PF3D7_1222300	PF3D7_1443900
0.00043	4	8.52	GO:0036094	small molecule binding	PF3D7_0708400	PF3D7_1118200	PF3D7_1222300	PF3D7_1443900
0.00062	4	7.71	GO:0016787	hydrolase activity	PF3D7_0708400	PF3D7_1118200	PF3D7_1222300	PF3D7_1443900
0.00107	4	6.63	GO:0005515	protein binding	PF3D7_0708400	PF3D7_1118200	PF3D7_1222300	PF3D7_1443900
0.00153	4	6.02	GO:0043167	ion binding	PF3D7_0708400	PF3D7_1118200	PF3D7_1222300	PF3D7_1443900
0.00519	4	4.34	GO:1901363	heterocyclic compound binding	PF3D7_0708400	PF3D7_1118200	PF3D7_1222300	PF3D7_1443900
0.00519	4	4.33	GO:0097159	organic cyclic compound binding	PF3D7_0708400	PF3D7_1118200	PF3D7_1222300	PF3D7_1443900
0.01267	4	3.43	GO:0003824	catalytic activity	PF3D7_0708400	PF3D7_1118200	PF3D7_1222300	PF3D7_1443900
0.02202	4	2.92	GO:0016020	membrane	PF3D7_0708400	PF3D7_1118200	PF3D7_1222300	PF3D7_1443900
0.02369	4	2.84	GO:0005737	cytoplasm	PF3D7_0708400	PF3D7_1118200	PF3D7_1222300	PF3D7_1443900
0.03449	4	2.55	GO:0005488	binding	PF3D7_0708400	PF3D7_1118200	PF3D7_1222300	PF3D7_1443900
0.07206	4	2.07	GO:0005622	intracellular anatomical structure	PF3D7_0708400	PF3D7_1118200	PF3D7_1222300	PF3D7_1443900
0.07697	4	2.01	GO:0009987	cellular process	PF3D7_0708400	PF3D7_1118200	PF3D7_1222300	PF3D7_1443900
0.10566	4	1.82	GO:0003674	molecular function	PF3D7_0708400	PF3D7_1118200	PF3D7_1222300	PF3D7_1443900
0.10637	4	1.81	GO:0008150	biological process	PF3D7_0708400	PF3D7_1118200	PF3D7_1222300	PF3D7_1443900
0.01067	1	167.44	GO:0019901	protein kinase binding	PF3D7_1118200			
0.01271	1	133.95	GO:0019900	kinase binding	PF3D7_1118200			
0.02218	1	70.5	GO:0006986	response to unfolded protein	PF3D7_1118200			
0.00088	2	59.53	Path:pfa04	protein processing in endoplasmic reticulum	PF3D7_0708400	PF3D7_1222300		
0.0291	1	51.52	GO:0035966	response to topologically incorrect protein	PF3D7_1118200			
0.00022	3	30.21	GO:0065008	regulation of biological quality	PF3D7_0708400	PF3D7_1222300	PF3D7_1443900	
0.04646	1	29.77	GO:0010033	response to organic substance	PF3D7_1118200			
0.00025	3	28.5	GO:0005886	plasma membrane	PF3D7_0708400	PF3D7_1222300	PF3D7_1443900	
0.00028	3	26.79	GO:0071944	cell periphery	PF3D7_0708400	PF3D7_1222300	PF3D7_1443900	
0.06058	1	22.33	GO:0019899	enzyme binding	PF3D7_1118200			
0.00039	3	21.26	GO:0033554	cellular response to stress	PF3D7_0708400	PF3D7_1222300	PF3D7_1443900	
0.07118	1	18.6	GO:0042221	response to chemical	PF3D7_1118200			
0.07531	1	16.96	GO:0005743	mitochondrial inner membrane	PF3D7_1118200			
0.07697	1	16.14	GO:0019866	organelle inner membrane	PF3D7_1118200			
0.07697	1	15.76	GO:0031966	mitochondrial membrane	PF3D7_1118200			
0.08183	1	14.56	GO:0005740	mitochondrial envelope	PF3D7_1118200			
0.01348	2	13.13	GO:0005783	endoplasmic reticulum	PF3D7_1222300	PF3D7_1443900		
0.00189	3	11.85	GO:0051716	cellular response to stimulus	PF3D7_0708400	PF3D7_1222300	PF3D7_1443900	
0.10099	1	11.45	GO:0031967	organelle envelope	PF3D7_1118200			
0.10099	1	11.35	GO:0031975	envelope	PF3D7_1118200			
0.00211	3	11.32	GO:0005829	cytosol	PF3D7_0708400	PF3D7_1222300	PF3D7_1443900	
0.03367	2	7.77	GO:0012505	endomembrane system	PF3D7_1222300	PF3D7_1443900		
0.01271	3	5.85	GO:0065007	biological regulation	PF3D7_0708400	PF3D7_1222300	PF3D7_1443900	
0.04441	3	3.57	GO:0032991	protein-containing complex	PF3D7_0708400	PF3D7_1222300	PF3D7_1443900	
0.16975	3	2.01	GO:0043231	intracellular membrane-bounded organelle	PF3D7_1118200	PF3D7_1222300	PF3D7_1443900	
0.18548	3	1.93	GO:0043227	membrane-bounded organelle	PF3D7_1118200	PF3D7_1222300	PF3D7_1443900	

Table S3: The Gene Ontology Enrichment of *P. knowlesi* Hsp90 isoforms

Enrichment FDR	nGenes	Fold Enrichment	GO TERM	DESCRIPTION	GENES		
1.65E-06	3	255.1	GO:0051082	unfolded protein binding	PKNH_0915900	PKNH_1238400	PKNH_1441400
1.03E-05	3	113.38	GO:0006457	protein folding	PKNH_0915900	PKNH_1238400	PKNH_1441400
2.31E-05	3	76.15	GO:0006950	response to stress	PKNH_0915900	PKNH_1238400	PKNH_1441400
2.97E-05	3	63.78	GO:0016887	ATP hydrolysis activity	PKNH_0915900	PKNH_1238400	PKNH_1441400
7.92E-05	3	42.87	GO:0050896	response to stimulus	PKNH_0915900	PKNH_1238400	PKNH_1441400
9.59E-05	3	34.24	GO:0016462	pyrophosphatase activity	PKNH_0915900	PKNH_1238400	PKNH_1441400
9.59E-05	3	33.13	GO:0016817	hydrolase activity acting on acid anhydrides	PKNH_0915900	PKNH_1238400	PKNH_1441400
9.59E-05	3	33.35	GO:0016818	hydrolase activity acting on acid anhydrides in phosphorus-containing anhydrides	PKNH_0915900	PKNH_1238400	PKNH_1441400
9.59E-05	3	36.71	GO:0017111	nucleoside-triphosphatase activity	PKNH_0915900	PKNH_1238400	PKNH_1441400
0.00022	3	24.3	GO:0140657	ATP-dependent activity	PKNH_0915900	PKNH_1238400	PKNH_1441400
0.00103	3	13.43	GO:0005524	ATP binding	PKNH_0915900	PKNH_1238400	PKNH_1441400
0.00103	3	13.32	GO:0030554	adenyl nucleotide binding	PKNH_0915900	PKNH_1238400	PKNH_1441400
0.00103	3	13.36	GO:0032559	adenyl ribonucleotide binding	PKNH_0915900	PKNH_1238400	PKNH_1441400
0.00118	3	12.44	GO:0005515	protein binding	PKNH_0915900	PKNH_1238400	PKNH_1441400
0.00122	3	11.52	GO:0016787	hydrolase activity	PKNH_0915900	PKNH_1238400	PKNH_1441400
0.00122	3	11.12	GO:0017076	purine nucleotide binding	PKNH_0915900	PKNH_1238400	PKNH_1441400
0.00122	3	11.04	GO:0032553	ribonucleotide binding	PKNH_0915900	PKNH_1238400	PKNH_1441400
0.00122	3	11.14	GO:0032555	purine ribonucleotide binding	PKNH_0915900	PKNH_1238400	PKNH_1441400
0.00122	3	11.19	GO:0035639	purine ribonucleoside triphosphate binding	PKNH_0915900	PKNH_1238400	PKNH_1441400
0.00122	3	10.93	GO:0097367	carbohydrate derivative binding	PKNH_0915900	PKNH_1238400	PKNH_1441400
0.00131	3	10.5	GO:0043168	anion binding	PKNH_0915900	PKNH_1238400	PKNH_1441400
0.00206	3	8.77	GO:0000166	nucleotide binding	PKNH_0915900	PKNH_1238400	PKNH_1441400
0.00206	3	8.77	GO:1901265	nucleoside phosphate binding	PKNH_0915900	PKNH_1238400	PKNH_1441400
0.00213	3	8.55	GO:0036094	small molecule binding	PKNH_0915900	PKNH_1238400	PKNH_1441400
0.00396	3	6.86	GO:0043167	ion binding	PKNH_0915900	PKNH_1238400	PKNH_1441400
0.00671	3	5.61	GO:0097159	organic cyclic compound binding	PKNH_0915900	PKNH_1238400	PKNH_1441400
0.00671	3	5.61	GO:1901363	heterocyclic compound binding	PKNH_0915900	PKNH_1238400	PKNH_1441400
0.01484	3	4.25	GO:0003824	catalytic activity	PKNH_0915900	PKNH_1238400	PKNH_1441400
0.02542	3	3.49	GO:0005488	binding	PKNH_0915900	PKNH_1238400	PKNH_1441400
0.02542	3	3.47	GO:0009987	cellular process	PKNH_0915900	PKNH_1238400	PKNH_1441400
0.03437	3	3.11	GO:0008150	biological process	PKNH_0915900	PKNH_1238400	PKNH_1441400
0.08176	3	2.3	GO:0003674	molecular function	PKNH_0915900	PKNH_1238400	PKNH_1441400

Table S4: Virtual screening of small molecule inhibitors against ADP-binding sites and associated lid domains in plasmodial and human Hsp90s

MolID	Name	RAW DATA						RANKED SCORE					
		PfHsp90α		PkHsp90α		HSPC1		PfHsp90α		PkHsp90α		HSPC1	
		ADP	GHL	ADP	GHL	ADP	GHL	ADP	GHL	ADP	GHL	ADP	GHL
1	17AAG	-5.9	-5.6	-5.7	-6.3	-7.5	-4.4	3	5	4	2	1	6
2	17A-Harmine3D	-6.5	-6	-6.3	-6	-7	-5.8	2	4	3	4	1	6
3	17DMAG	-6.7	-5.7	-7.1	-7.1	-5.8	-4.4	3	5	1	1	4	6
4	21A-Harmine3D	-6.4	-5.9	-6.3	-6.1	-6.6	-5.9	2	5	3	4	1	5
5	3D_ATP	-7.4	-6.8	-7.2	-6.4	-8.3	-6.1	2	4	3	5	1	6
6	ADP	-6.9	-6.8	-6.8	-6.7	-8.1	-5.8	2	3	3	5	1	6
7	AT13387	-8.9	-8.2	-8.8	-8	-8.5	-6.4	1	4	2	5	3	6
8	ATP	-7.3	-6.6	-7.7	-6.5	-7.8	-5.6	3	4	2	5	1	6
9	BX-2819	-8	-7	-7.7	-6.9	-9	-5.5	2	4	3	5	1	6
10	Dihydroartemisinin	-7.7	-7.3	-7.4	-7.4	-7.7	-6	1	5	3	3	1	6
11	Geldanamycin	-7.5	-5.7	-7.5	-7.6	-7	-4.3	2	5	2	1	4	6
12	Harmine	-6.8	-6.3	-6.6	-6.2	-6.7	-5.5	1	4	3	5	2	6
13	IMA-1	-6.5	-6.1	-6.5	-6.4	-7.1	-6.1	2	5	2	4	1	5
14	IMA-2	-6.5	-6.4	-6.4	-6.3	-7.4	-5.3	2	3	3	5	1	6
15	IMA-3	-6.5	-5.6	-6.6	-5.5	-6.5	-5.7	2	5	1	6	2	4
16	MMV019066	-7.4	-7.6	-7.4	-7.2	-7.4	-5.7	2	1	2	5	2	6
17	N-CBZ-1A	-8.6	-8.7	-8.9	-8.6	-8.5	-5.9	3	2	1	3	5	6
18	N-CBZ-1B	-7.9	-8.5	-8.8	-8.2	-8.5	-5.8	5	2	1	4	2	6
19	N-CBZ-1C	-6.3	-6.3	-6.9	-6.5	-7	-5.2	4	4	2	3	1	6
20	N-CBZ-1D	-6.4	-6.8	-7	-6.4	-6.9	-5.6	4	3	1	4	2	6
21	N-CBZ-1E	-7.5	-7	-7.6	-7.2	-7.7	-6.4	3	5	2	4	1	6
22	N-CBZ-1F	-7.4	-6.6	-7.5	-7	-7.1	-6.2	2	5	1	4	3	6
23	N-CBZ-1G	-7.8	-6.9	-7.7	-7	-7.9	-5.8	2	5	3	4	1	6
24	N-CBZ-1H	-7.6	-7.4	-8.6	-7.3	-8	-6.1	3	4	1	5	2	6
25	N-CBZ-2A	-7.7	-7.5	-8	-7.3	-8.2	-6.7	3	4	2	5	1	6
26	N-CBZ-2B	-8.5	-8.1	-8.5	-8.1	-7.6	-5.8	1	3	1	3	5	6
27	N-CBZ-2C	-7.5	-6.5	-7.4	-6.7	-7.7	-5.9	2	5	3	4	1	6
28	N-CBZ-2D	-8.6	-7.9	-8.5	-8.1	-7.7	-5.4	1	4	2	3	5	6
29	N-CBZ-2E	-8.1	-7	-8	-7.1	-7.8	-5.8	1	5	2	4	3	6
30	N-CBZ-2F	-7	-6.2	-6.4	-6.2	-6.6	-5.7	1	4	3	4	2	6
31	N-CBZ-2G	-6.9	-6.3	-6.6	-6.4	-7.3	-5.5	2	5	3	4	1	6
32	N-CBZ-2H	-8.9	-8.4	-8.8	-8.4	-8.2	-5.2	1	3	2	3	5	6
33	N-CBZ-3A	-8.9	-8.4	-9.3	-8.5	-7.9	-6.2	2	4	1	3	5	6
34	N-CBZ-3B	-8.7	-9	-9.5	-9.1	-8.2	-5.2	4	3	1	2	5	6
35	N-CBZ-3C	-9	-8.4	-8.9	-8.2	-7.9	-5.8	1	3	2	4	5	6
36	N-CBZ-3D	-7	-5.9	-6.6	-6.3	-7.4	-5.2	2	5	3	4	1	6
37	N-CBZ-3E	-9.2	-8.5	-9.3	-8.4	-8.2	-5.6	2	3	1	4	5	6
38	N-CBZ-3F	-7.8	-6.9	-7.7	-7.1	-8.1	-6.1	2	5	3	4	1	6

39	N-CBZ-3G	-9.1	-8.5	-9.1	-8.5	-7.8	-5.9	1	3	1	3	5	6
40	N-CBZ-3H	-7.4	-7	-7.4	-7.3	-8.5	-6	2	5	2	4	1	6
41	N-CBZ-4A	-6.6	-6.5	-6.5	-6.2	-7.2	-5.9	2	3	3	5	1	6
42	N-CBZ-4B	-7.1	-6.9	-7	-6.6	-7.8	-6.2	2	4	3	5	1	6
43	N-CBZ-5A	-8	-7	-7.9	-7.1	-8	-6.2	1	5	3	4	1	6
44	N-CBZ-5B	-8.9	-8.8	-9	-8.7	-8.3	-6.2	2	3	1	4	5	6
45	N-CBZ-5E	-6.9	-6.6	-7	-6.8	-7.2	-5.4	3	5	2	4	1	6
46	N-CBZ-5G	-6.5	-6.6	-6.6	-6.7	-7.1	-5.5	5	3	3	2	1	6
47	NVP-AUY922	-8.4	-7.3	-7.9	-7.4	-8.2	-5.5	1	5	3	4	2	6
48	PUH71	-7.6	-7	-7.3	-6.7	-7.4	-5.1	1	4	3	5	2	6
49	Radicicol	-8.7	-6.5	-8.3	-7	-9.2	-6	2	5	3	4	1	6
50	SNX-0723	-8.7	-7.5	-8.7	-7.6	-8.8	-6.2	2	5	2	4	1	6
51	SNX-2112	-9.7	-8.6	-9.7	-8.2	-9.2	-7.4	1	4	1	5	3	6
52	SNX-5422	-9.9	-8.1	-9.3	-7.7	-9.3	-6.1	1	4	2	5	2	6
53	STA-9090	-8.4	-7.4	-7.9	-7.3	-9	-5.3	2	4	3	5	1	6
54	Tropane	-4.5	-4.2	-4.4	-4.4	-4.2	-3.8	1	4	2	2	4	6
55	UAA	-6.9	-7.5	-7.4	-7.7	-7.6	-6.5	5	3	4	1	2	6
56	XL888	-8.3	-7.6	-7.6	-7.7	-8.9	-6	2	4	4	3	1	6
57	ZINC01325114	-10	-7.9	-9.8	-7.7	-9.5	-6.5	1	4	2	5	3	6
58	ZINC01334804	-10.6	-9	-10.3	-8.5	-9.4	-7.4	1	4	2	5	3	6
59	ZINC01787663	-10	-9.3	-10	-9.1	-10.5	-7.1	2	4	2	5	1	6
60	ZINC02101693	-11.1	-8.3	-10.6	-8.5	-10.2	-7.9	1	5	2	4	3	6
61	ZINC02118360	-10.9	-9	-10.5	-9	-10.6	-6.6	1	4	3	4	2	6
62	ZINC02256691	-10.9	-9	-10.6	-8.9	-10	-7.5	1	4	2	5	3	6
63	ZINC02634823	-10.4	-9.1	-10.5	-9.2	-9.4	-5.9	2	5	1	4	3	6
64	ZINC04482555	-10.7	-9.5	-10.6	-9.4	-8.8	-7.5	1	3	2	4	5	6
65	ZINC06161828	-10.4	-8	-10.1	-8	-8.9	-5.7	1	4	2	4	3	6
66	ZINC08324881	-10.3	-8.3	-10	-8.1	-10.4	-6.4	2	4	3	5	1	6
67	ZINC08725823	-8.8	-9.4	-8.8	-8.2	-8.9	-6.4	3	1	3	5	2	6
68	ZINC08846332	-10.9	-8.1	-11	-8.7	-8.9	-6.7	2	5	1	4	3	6
69	ZINC08855704	-10.4	-9.7	-10.4	-9.1	-9	-6.1	1	3	1	4	5	6
70	ZINC08860816	-11	-8.8	-10.5	-7.4	-8.6	-6	1	3	2	5	4	6
71	ZINC09406004	-9	-8	-9	-7.7	-8.4	-6.3	1	4	1	5	3	6
72	ZINC09428796	-10.4	-9	-10.3	-8.9	-9.2	-5.9	1	4	2	5	3	6
73	ZINC09477638	-10.6	-8.4	-10.3	-7.8	-10.6	-6.5	1	4	3	5	1	6
74	ZINC09528925	-10.5	-8.4	-10.1	-8.2	-10	-6	1	4	2	5	3	6
75	ZINC09579126	-9.8	-8.2	-9.4	-8.4	-9	-7	1	5	2	4	3	6
76	ZINC09781842	-10.1	-9	-9.8	-8.8	-9.8	-7.1	1	4	2	5	2	6
77	ZINC09782620	-10.8	-9.2	-10.6	-9.1	-9	-7.1	1	3	2	4	5	6
78	ZINC09845364	-11.6	-8.5	-11.1	-8.2	-10.2	-6.1	1	4	2	5	3	6
79	ZINC09845480	-10.8	-9.2	-11	-9	-10.7	-5.9	2	4	1	5	3	6
80	ZINC09845582	-11.6	-9.8	-10.5	-9.1	-11	-6.1	1	4	3	5	2	6
81	ZINC09849301	-10	-9.6	-10.8	-9.3	-8.7	-6.7	2	3	1	4	5	6

82	ZINC09849905	-10.5	-9.3	-10.1	-9.1	-9.1	-6.6	1	3	2	4	4	6
83	ZINC09851834	-10.6	-9.4	-11	-9.6	-8.5	-7.8	2	4	1	3	5	6
84	ZINC09858888	-10.5	-8.7	-9.8	-8.9	-10.6	-7	2	5	3	4	1	6
85	ZINC09860237	-10.3	-10	-10.1	-10.1	-10.3	-5.6	1	5	3	3	1	6
86	ZINC09882065	-9.9	-9	-9.4	-8.9	-10	-6.7	2	4	3	5	1	6
87	ZINC11085391	-9.4	-7.5	-9.1	-7.8	-9.4	-6.7	1	5	3	4	1	6
88	ZINC11666009	-11	-10.1	-10.3	-10	-9.3	-8.2	1	3	2	4	5	6
89	ZINC11865353	-8.5	-8.3	-8.6	-8.9	-9.2	-7.3	4	5	3	2	1	6
90	ZINC11865356	-9.2	-8.8	-9.1	-8.8	-9.4	-6.2	2	4	3	4	1	6
91	ZINC11865361	-9.9	-8.2	-9.2	-8.3	-8.2	-7	1	4	2	3	4	6
92	ZINC11867632	-10.3	-9.2	-10.1	-9.7	-10	-6.6	1	5	2	4	3	6
93	ZINC11881900	-9.3	-9	-8.9	-9.8	-8.8	-7.9	2	3	4	1	5	6
94	ZINC11935121	-9.5	-8.2	-9.5	-8.9	-9.4	-6.5	1	5	1	4	3	6
95	ZINC12009300	-9.9	-8.9	-10.3	-9.3	-9.6	-6.7	2	5	1	4	3	6
96	ZINC12026560	-9.2	-10	-9.6	-9.4	-9.5	-6.6	5	1	2	4	3	6
97	ZINC12086059	-10.6	-9.6	-10.6	-9.7	-9.8	-7.1	1	5	1	4	3	6
98	ZINC12274455	-11.4	-9.1	-11.3	-8.9	-9.5	-7.4	1	4	2	5	3	6
99	ZINC12310222	-10.6	-9.6	-10.4	-9.6	-8.9	-6.4	1	3	2	3	5	6
100	ZINC12323081	-11.1	-8.8	-10.8	-8.9	-10.2	-6.7	1	5	2	4	3	6
101	ZINC12323082	-11.2	-8.7	-11	-8.7	-10.3	-7	1	4	2	4	3	6
102	ZINC12444335	-10.7	-8.7	-10.9	-7.8	-9.3	-5.4	2	4	1	5	3	6
103	ZINC12527935	-9.4	-7.9	-9.4	-7.9	-9	-6.6	1	4	1	4	3	6
104	ZINC12566950	-9.6	-8.8	-9.1	-8.9	-9.7	-6.9	2	5	3	4	1	6
105	ZINC12568263	-8.9	-7.6	-8.6	-7.6	-8.9	-5.7	1	4	3	4	1	6
106	ZINC12597225	-9.2	-9.5	-9.3	-9.2	-9.8	-6.9	4	2	3	4	1	6
107	ZINC12676980	-8.9	-8.8	-9.2	-9	-9.1	-6.4	4	5	1	3	2	6
108	ZINC12785678	-11.9	-8.9	-11.8	-8.7	-9.7	-6.8	1	4	2	5	3	6
109	ZINC13114217	-10.6	-8.4	-10.8	-8.4	-10.3	-6.7	2	4	1	4	3	6
110	ZINC13164823	-11.1	-8.6	-10.9	-8.8	-9.7	-7.1	1	5	2	4	3	6
111	ZINC13644481	-10.6	-9.5	-9.9	-8	-9.1	-8.5	1	3	2	6	4	5
112	ZINC14058181	-11.1	-10.5	-11	-10.5	-9.5	-6.5	1	3	2	3	5	6
113	ZINC14141808	-10.2	-8.4	-10	-8.2	-7.8	-6.6	1	3	2	4	5	6
114	ZINC14475202	-9.7	-8.7	-9.8	-8.9	-9.4	-6.6	2	5	1	4	3	6
115	ZINC14544860	-10.2	-9.4	-10.2	-9.3	-9.8	-6.8	1	4	1	5	3	6
116	ZINC14545108	-10.9	-9.4	-9.9	-9	-10.3	-6.7	1	4	3	5	2	6
117	ZINC14883049	-9.8	-7.6	-9.4	-7.8	-9.2	-7.1	1	5	2	4	3	6
118	ZINC14994602	-10.3	-8	-9.3	-9	-8.9	-6.2	1	5	2	3	4	6
119	ZINC15003445	-10.6	-8.5	-10.6	-8.7	-9.7	-6.6	1	5	1	4	3	6
120	ZINC15225981	-11.1	-9.3	-9.7	-9.2	-9.2	-7.5	1	3	2	4	4	6
121	ZINC15864991	-9.6	-7.6	-9.5	-8.2	-9.4	-6.3	1	5	2	4	3	6
122	ZINC16524774	-10.4	-9.9	-10.7	-9.6	-9.8	-8.6	2	3	1	5	4	6
123	ZINC18948369	-9.7	-8.1	-9.2	-8.5	-8.5	-6.2	1	5	2	3	3	6
124	ZINC19399617	-11.2	-11.1	-11.4	-10.5	-9.2	-6.6	2	3	1	4	5	6

125	ZINC19698473	-9.1	-8.2	-9.8	-8.4	-8.6	-6.3	2	5	1	4	3	6
126	ZINC20152897	-10.9	-8.5	-9.7	-8.6	-9.4	-6	1	5	2	4	3	6
127	ZINC20152899	-10.4	-9	-9.6	-9.1	-9.3	-6.7	1	5	2	4	3	6
128	ZINC20152905	-10.3	-9.4	-9.8	-9.1	-9.3	-7	1	3	2	5	4	6
129	ZINC20355806	-10.9	-8.9	-10.7	-9.1	-10.2	-7.1	1	5	2	4	3	6
130	ZINC20564114	-10.1	-9	-9.5	-9.5	-9	-6.7	1	4	2	2	4	6
131	ZINC20574318	-10.9	-8.6	-10.5	-8.9	-9.8	-7	1	5	2	4	3	6
132	ZINC20760873	-10.1	-9.3	-9.9	-9.6	-9	-7	1	4	2	3	5	6
133	ZINC20761095	-9.5	-7.9	-9.2	-8.8	-9.4	-6.3	1	5	3	4	2	6
134	ZINC20854729	-10.5	-8.9	-10.4	-9.1	-8.9	-8.9	1	4	2	3	4	4
135	ZINC21089537	-11.1	-8.6	-10.8	-8.9	-9.4	-6.5	1	5	2	4	3	6
136	ZINC21171169	-11.3	-9.2	-11.2	-9.2	-9.4	-7.2	1	4	2	4	3	6
137	ZINC22007970	-11.4	-10.5	-11.3	-10.4	-10.3	-6.9	1	3	2	4	5	6
138	ZINC23968403	-11.3	-8.6	-10.6	-8.8	-9.9	-7.2	1	5	2	4	3	6
139	ZINC24707629	-9.8	-9.2	-10	-9.2	-9	-6.6	2	3	1	3	5	6
140	ZINC24895178	-11.2	-8.7	-10.5	-9.3	-10.7	-7.4	1	5	3	4	2	6
141	ZINC24895663	-10.3	-8.4	-10.1	-8.9	-8.9	-6.5	1	5	2	3	3	6
142	ZINC24895904	-10.5	-8.7	-10.3	-8.5	-9.7	-6.5	1	4	2	5	3	6
143	ZINC25150850	-9.7	-8	-10.5	-8	-8.4	-7.4	2	4	1	4	3	6
144	ZINC25150853	-8.1	-8.1	-9.9	-8.1	-8.3	-5.9	3	3	1	3	2	6
145	ZINC29990352	-9.6	-9.1	-10.3	-9.2	-9.8	-6.4	3	5	1	4	2	6
146	ZINC33028239	-9.4	-8.4	-8.9	-8.3	-8.8	-6	1	4	2	5	3	6
147	ZINC33084784	-9.9	-7.7	-9.7	-8.2	-9.8	-6.4	1	5	3	4	2	6
148	ZINC33085016	-10.7	-8.7	-10.7	-8.6	-9.7	-7	1	4	1	5	3	6
149	ZINC33140622	-9.2	-9.1	-8.9	-9.3	-7.7	-7.3	2	3	4	1	5	6
150	ZINC33261888	-11.4	-8.9	-11	-8.7	-9.7	-7.5	1	4	2	5	3	6
151	ZINC33265740	-8.8	-8.5	-8.5	-8.6	-8.9	-6.4	2	4	4	3	1	6
152	ZINC33281832	-10.6	-8.8	-10.2	-8	-8.5	-6.5	1	3	2	5	4	6
153	ZINC33284113	-10.3	-8.6	-10.2	-9.1	-10.5	-6.5	2	5	3	4	1	6
154	ZINC33284118	-9.9	-10	-10.4	-9.9	-10.7	-7	4	3	2	4	1	6
155	ZINC33297589	-10.7	-9.2	-10.5	-9.4	-8.4	-6	1	4	2	3	5	6
156	ZINC34792088	-10.7	-8.2	-10.4	-8.6	-9.1	-6.4	1	5	2	4	3	6
157	ZINC34983862	-10.2	-9.1	-11	-8.4	-9.2	-6.6	2	4	1	5	3	6
158	ZINC34988270	-10.7	-9.1	-10.9	-9	-9	-7.3	2	3	1	4	4	6
159	ZINC35399602	-9.1	-8.5	-9.1	-8.5	-9.7	-6.7	2	4	2	4	1	6
160	ZINC35515855	-11.1	-8.7	-10.9	-9	-9.6	-6.7	1	5	2	4	3	6
161	ZINC35515875	-11.1	-10	-11	-9.9	-9.2	-7.3	1	3	2	4	5	6
162	ZINC35945586	-8.5	-8.2	-8.1	-8.4	-8.2	-6.4	1	3	5	2	3	6
163	ZINC38167083	-11.1	-10.6	-11.2	-10.6	-10.2	-7.2	2	3	1	3	5	6
164	ZINC38714313	-10.2	-9.8	-9.9	-10	-8.8	-6.9	1	4	3	2	5	6
165	ZINC38942570	-10.9	-9.9	-10.7	-9.8	-8.9	-6.9	1	3	2	4	5	6
166	ZINC38943289	-10.3	-9.7	-10.2	-9.6	-8.7	-6.6	1	3	2	4	5	6
167	ZINC38944925	-9.5	-8.1	-9.3	-8	-9.2	-7.4	1	4	2	5	3	6

168	ZINC38944954	-10.1	-8.5	-9.8	-8.6	-9.2	-7.2	1	5	2	4	3	6
169	ZINC39344506	-9.9	-8.1	-9.5	-8.1	-8	-6.6	1	3	2	3	5	6
170	ZINC39500733	-9.5	-8.6	-9.6	-8.5	-9.6	-6.8	3	4	1	5	1	6
171	ZINC39737537	-10.2	-9.7	-10.2	-10	-10.1	-7.2	1	5	1	4	3	6
172	ZINC39852148	-10.6	-9.8	-10.9	-9.8	-9.6	-7.6	2	3	1	3	5	6
173	ZINC39890691	-8.8	-9	-10.2	-8.9	-10.8	-5.8	5	3	2	4	1	6
174	ZINC39921210	-9.5	-10	-10.2	-9.8	-10.8	-7.1	5	3	2	4	1	6
175	ZINC49006508	-10.5	-8	-10	-8.1	-9.1	-6.8	1	5	2	4	3	6
176	ZINC49714008	-9.8	-8.7	-9.8	-8.8	-8.4	-7.2	1	4	1	3	5	6
177	ZINC64818203	-10.8	-9.9	-10.1	-9.8	-8.8	-6.6	1	3	2	4	5	6
178	ZINC64818257	-10.2	-9.5	-10	-9.4	-8.4	-6.2	1	3	2	4	5	6
179	ZINC67263318	-11.6	-9	-11.2	-8.3	-9.1	-6.7	1	4	2	5	3	6
180	ZINC71895161	-10.2	-9.6	-9.8	-9.8	-9.2	-6.8	1	4	2	2	5	6
181	ZINC72048808	-10.9	-9.9	-12	-9.5	-10.2	-6.9	2	4	1	5	3	6
182	ZINC72266014	-10.2	-9	-10	-8.6	-8.9	-6.8	1	3	2	5	4	6
183	ZINC72280435	-10.9	-8.5	-10.5	-8.7	-10.4	-7.3	1	5	2	4	3	6
184	ZINC72341267	-9.1	-8.6	-9.1	-8.7	-8.2	-6.8	1	4	1	3	5	6
185	ZINC72358966	-8.9	-8.9	-8.9	-8.8	-9.8	-6.2	2	2	2	5	1	6
186	ZINC72444392	-9.6	-8.2	-9.6	-8.3	-8.9	-7.4	1	5	1	4	3	6
187	ZINC72461072	-11.6	-10.7	-11.4	-10.4	-10.3	-7.5	1	3	2	4	5	6
188	ZINC72461078	-11.6	-10.2	-11.4	-10.3	-11	-7.3	1	5	2	4	3	6
189	ZINC72472309	-11.3	-10.3	-11.1	-10.3	-9.7	-7.4	1	3	2	3	5	6
190	ZINC73686164	-11.8	-10	-11.2	-9.9	-9.9	-8.1	1	3	2	4	4	6
191	ZINC77972673	-9.7	-9	-9.5	-8.9	-8.8	-6.6	1	3	2	4	5	6
192	ZINC77973062	-9	-8	-9	-7.9	-8.7	-5.8	1	4	1	5	3	6
193	ZINC77973620	-9.3	-8.7	-10.3	-8.9	-8.4	-6.7	2	4	1	3	5	6
194	ZINC95474733	-10.7	-9	-10.3	-8.9	-9.5	-7	1	4	2	5	3	6
195	ZINC95476565	-8.6	-8.8	-8.5	-8	-10	-6.2	3	2	4	5	1	6

Table S5: Plasmodial and human Hsp90s used in this study

Localization	Hsp90 Isoforms	Formal Name	Accession Number		Other Names
			UniProt ID	NCBI ID	
Cytosol	Hsp90α	HSPC1	P07900	3320	HSP90AA1; HSP90A; HSPCA
Cytosol	HSP90AA2	HSPC2	Q14568	3324	HSP90AA2P; Hsp90α-2
Cytosol	Hsp90β	HSPC3	P08238	3326	HSP90AB1
Endoplasmic Reticulum	GRP94	HSPC4	P14625	7184	HSP90B1
Mitochondrion	TRAP1	HSPC5	Q12931	10131	HSP75, HSP90L
Cytosol	PBANKA_0805700	PbHsp90	A0A509ANB9	55149125	
Mitochondrion	PBANKA_0929900	PbTRAP1	A0A509AK21	55149754	
Endoplasmic Reticulum	PBANKA_1437300	PbGRP94	A0A509B0M9	55152412	
Apicoplast	PBANKA_1307800	PbHsp90_A	A0A509ARE3	55151419	
Cytosol	PF3D7_0708400	PfHsp90	Q8IC05	2655065	
Mitochondrion	PF3D7_1118200	PfTRAP1	Q8III6	810735	
Endoplasmic Reticulum	PF3D7_1222300	PfGRP94	Q8I0V4	811266	
Apicoplast	PF3D7_1443900	PfHsp90_A	Q8IL32	811999	
Cytosol	PKNH_0107000	PkHsp90	A0A679KRE8	7318567	
Mitochondrion	PKNH_0915900	PkTRAP1	A0A384K8H3	7320745	
Endoplasmic Reticulum	PKNH_1441400	PkGRP94	A0A384LP92	7323311	
Apicoplast	PKNH_1238400	PkHsp90_A	A0A384K893	7321969	
Cytosol	PVX_087950	PvHsp90	A0A1G4GQX1	5472715	PVP01_0108700
Mitochondrion	PVX_091545	PvTRAP1	A0A564ZUN9	5474596	PVP01_0918800
Endoplasmic Reticulum	PVX_123745	PvGRP94	A0A1G4H539	5476620	PVP01_1440500
Apicoplast	PVX_118295	PvHsp90_A	A0A1G4H2J6	5475250	PVP01_1263100
Cytosol	PY17X_0808800	PyHsp90	A0A078K356	3801711	
Mitochondrion	PY17X_0931900	PyTRAP1	A0A078K7S5	3790988	
Endoplasmic Reticulum	PY17X_1439800	PyGRP94	A0A077YF64	3790807	
Apicoplast	PY17X_1311600	PyHsp90_A	A0A078KJX6	3807045	
Cytosol	TA12105	TaHsp90	Q4UDU8	3862045	XP_954026.1
Mitochondrion	TA06845	TaTRAP1	Q4UHU0	3863760	XP_954026.1
Endoplasmic Reticulum	TA06470	TaGRP94	Q4UIC5	3864533	XP_952473.1
Apicoplast	TA10720	TaHsp90_A	Q4U942	3862650	XP_953286.1
Cytosol	BOVATA_007200	BoHsp90	A0A2H6K8A2	39872997	
Mitochondrion	BOVATA_017520	BoTRAP1	A0A2H6KBC0	39874029	XP_028866502.1
Endoplasmic Reticulum	BOVATA_020650	BoGRP94	A0A2H6KC60	39874342	XP_028866815.1
Apicoplast	BOVATA_003160	BoHsp90_A	A0A2H6K745	39872593	XP_028865066.1
Chloroplast	LOC107797756	NtHsp90_A	A0A1S4AHN5	107797756	BAX37185.1

Interacting Partners of PfHsp90		Orthologs in *P. knowlesi*
Former Accession Number	New Accession Number	
MAL7P1.118	PF3D7_0722100	PKNH_0317500
PFL1070c	PF3D7_1222300	PKNH_1441400
PF07_0033	PF3D7_0708800	PKNH_0107400
PF07_0086	PF3D7_0721000	PKNH_0316400
PF10_0115	PF3D7_1011800	PKNH_0811600
PF10_0171	PF3D7_1017600	
PF10_0242	PF3D7_1024800	PKNH_0609300
PF14_0032	PF3D7_1403400	PKNH_1354700
PF14_0700	PF3D7_1473200	PKNH_1207800
PF14_0708	PF3D7_1474000	
PFF0915w	PF3D7_0618900	PKNH_1131500
PFI0930c	PF3D7_0919000	PKNH_0717000
PFI1610c	PF3D7_0933200	PKNH_0731900
PFL0130c	PF3D7_1202600	PKNH_1302500
PFL2430c	PF3D7_1250600	PKNH_1470600
PF07_0029	PF3D7_0708400	PKNH_0107000
PF14_0324/HOP	PF3D7_1434300	PKNH_0420900
